# Supplementary material for: Non‐apoptotic caspase activation ensures the homeostasis of ovarian somatic stem cells
Source: EMBO Rep. 2023 Apr 11;24(6):e51716. doi: 10.15252/embr.202051716 (PMC10240206; doi:10.15252/embr.202051716)
Supplement: Supplementary file 1 — Appendix [file EMBR-24-e51716-s007.pdf]

Table of contents

Appendix Figure S1. Figure showing that *Dronc* deficiency promotes the Ptc-dependent induction of autophagy that ultimately alters the cellular properties ovarian somatic cells. Pages 2-3.

Appendix Table S1. Description of fly lines and genotypes used in the experiments of Main Figures. Pages 4-7

Appendix Table S2. Description of fly lines and genotypes used in the experiments of Expanded View and Appendix Figures. Pages 8-9

Appendix Figure S2. Figure describing the counting methodology used for follicular cells throughout the manuscript. Pages 10-11

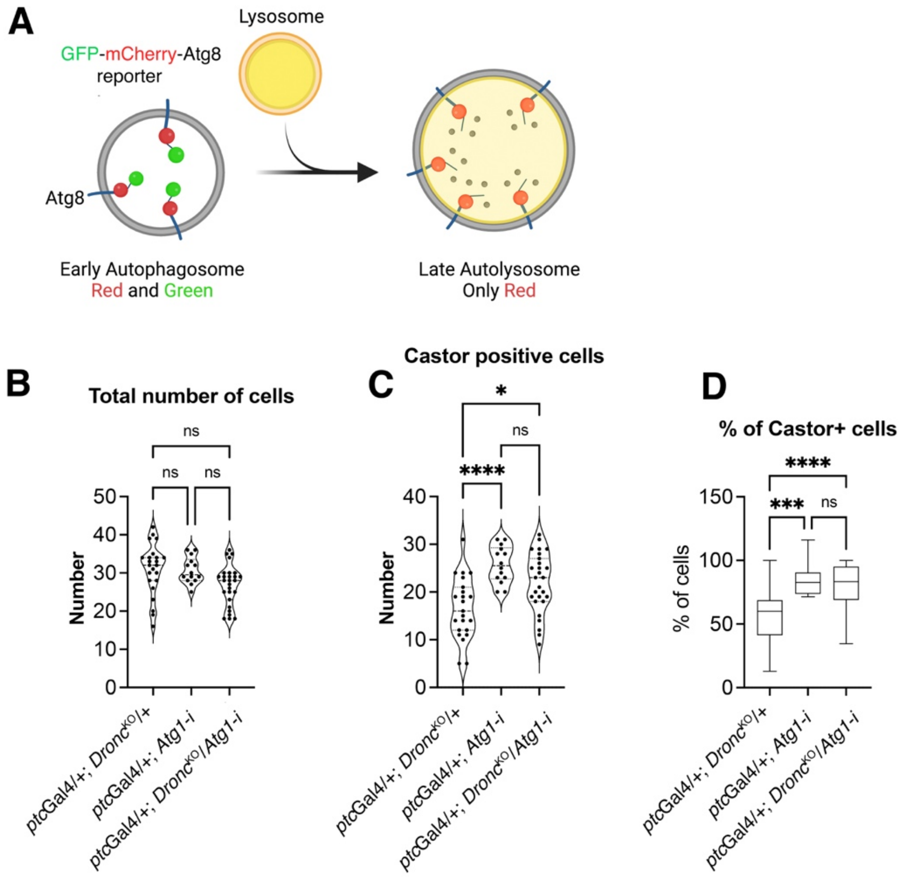

Appendix Figure S1.

A. Schematic depicting the labelling of early autophagosomes (green and red) and late autophagolysosomes (only red) using GFP-Cherry-Atg8. Diagram generated with BioRender.

B. Quantification of total number of follicular cells within the FasIII cellular domain in the following genotypes:

ptc-Gal4 (BL2017)/+; *Dronc*^{KO}Tub-*G80*^{ts} (BL7019)/+. (n=23)

ptc-Gal4 (BL2017)/+; Tub-*G80*^{ts} (BL7019)/UAS-*Atg1*-RNAi (BL35177). (n=14)

ptc-Gal4 (BL2017)/+; *Dronc*^{KO}Tub-*G80*^{ts} (BL7019)/UAS-*Atg1*-RNAi (BL35177). (n=27)

A Kruskal-Wallis test and Dunn's multiple comparison post-test were used to determine statistical significance (n.s.= not significant).

C. Quantification of the total number of Castor-expressing cells within the FasIII cellular domain in the genotypes described in B. n number is indicated also in B. An ordinary one-way ANOVA and Dunnett's multiple comparison post-test were used to determine statistical significance (n.s.= not significant; * p≤0.05; **** p≤0.0001).

D. Percentage of Castor-expressing cells versus the total number of Follicular cells (FasIII$^+$ cells) in germaria of the genotypes indicated in B. n numbers are shown in B. Data are expressed as box-and-whiskers plots, with min to max range as whiskers. A Kruskal-Wallis test and Dunn's multiple comparison post-test were used to determine statistical significance (n.s.= not significant; ***p≤0.001; ****p≤0.0001).

Data information. Experimental flies were kept after eclosion from the pupae for 14 days at 29°C prior to dissection. All the experimental data shown have been obtained from N ≥ 2 biological replicates. The median and quartiles are indicated in the violin plots. The box plot shows the median, first quartile, and third quartile of the dataset. The whiskers illustrate the range between the maximum and minimum values of the dataset. All the quantifications were made in germaria containing one single group of germline cells wrapped by follicular cells.

**Genotypes and fly lines information**	
**Figure**	**Genotype**
**1B**	Actin *DBS-S-QF*, UAS-*mCD8-GFP*, QUAS-*tomato-HA*/+; ;QUAS-*Gal4 /*+ (BL83123)
**1C**	Actin *DBS-S-QF*, UAS-*mCD8-GFP*, QUAS-*tomato-HA*/+; QUAS-*flippase* (BL30126)/+;<br>Actin5C FRT-*stop*-FRT *lacZ*-nls/+ (BL6355)
**1D-F**	Actin *DBS-S-QF*, UAS-*mCD8-GFP*, QUAS-*tomato-HA*/+; QUAS-*flippase* (BL30126)/+;<br>Actin5C FRT-*stop*-FRT *lacZ*-nls/+ (BL6355)
**1G**	**Control:**<br>Actin *DBS-S-QF*, UAS-*mCD8-GFP*, QUAS-*tomato-HA*/+; *ptc-Gal4* (BL2017)/+<br><br>**Experimental:**<br>Actin *DBS-S-QF*, UAS-*mCD8-GFP*, QUAS-*tomato-HA*/+; *ptc-Gal4* (BL2017)/UAS-*Dronc*RNAi (a gift from Pascal Meier)
**1I**	w;; *Dronc*$^{\text{KO-Gal4}}$ / UAS-*Histone-RFP* (BL56555)
**1J**	w;; *Dronc*^{TurboID} (a gift from Masayuki Miura)/ Tm3, Sb
**2A, 2B**	**Control:**<br>*109-30-Gal4* (BL7023)/+; *Dronc*^{KO} UAS-*Histone-RFP* (BL56555) Tub-*G80*^{ts} (BL7019)/+<br><br>**Experimental without Dronc expression:**<br>*109-30-Gal4* (BL7023)/+; *Dronc*^{KO} UAS-*Histone-RFP* (BL56555) Tub-*G80*^{ts} (BL7019)/ UAS-*flippase* (BL8209) *Dronc*$^{\text{KO-FRT-Dronc-GFP-APEX-FRT-QF}}$
**2C, 2D**	**Control:**<br>*109-30-Gal4* (BL7023)/+; *Dronc*^{KO} UAS-*Histone-RFP* (BL56555) Tub-*G80*^{ts} (BL7019)/+;<br>**Experimental without Dronc expression:**<br>*109-30-Gal4* (BL7023)/+; *Dronc*^{KO} UAS-*Histone-RFP* (BL56555) Tub-*G80*^{ts} (BL7019)/ UAS-*flippase* (BL8209) *Dronc*$^{\text{KO-FRT-Dronc-GFP-APEX-FRT-QF}}$.<br><br>**Control:**<br>*ptc-Gal4* (BL2017)/+; UAS-*Histone-RFP* (BL56555) Tub-*G80*^{ts} (BL7019)/+<br>**Experimental without Dronc expression:**<br>*ptc-Gal4* (BL2017)/+; *Dronc*^{KO} UAS-*Histone-RFP* (BL56555) Tub-*G80*^{ts} (BL7019)/ UAS-*flippase* (BL8209) *Dronc*$^{\text{KO-FRT-Dronc-GFP-APEX-FRT-QF}}$.
**2E**	**White triangle** = *ptc-Gal4* (BL2017)/+; +/+<br><br>**Yellow circle** = *ptc-Gal4* (BL2017)/+; *Dronc*^{KO} UAS-*Histone-RFP* (BL56555) Tub-*G80*^{ts} (BL7019)/ +<br><br>**Red square** = *ptc-Gal4* (BL2017)/+; *Dronc*^{KO} UAS-*Histone-RFP* (BL56555) Tub-*G80*^{ts} (BL7019)/ UAS-*flippase* (BL8209) *Dronc*$^{\text{KO-FRT-Dronc-GFP-APEX-FRT-suntag-HA}}$<br><br>**Blue circle** = *ptc-Gal4* (BL2017)/UAS-*Dronc* (BL56198); *Dronc*^{KO} UAS-*Histone-RFP* (BL56555) Tub-*G80*^{ts} (BL7019)/+
**2F**	*ptc-Gal4* (BL2017)/+; *Dronc*^{KO} UAS-*Histone-RFP* (BL56555) Tub-*G80*^{ts} (BL7019)/ +
**2G**	*ptc-Gal4* (BL2017)/+; *Dronc*^{KO} UAS-*Histone-RFP* (BL56555) Tub-*G80*^{ts} (BL7019)/UAS-*Dronc* (BL56198)

**2I-K**	**Control:**<br>*109-30-Gal4* (BL7023)/UAS-FUCCI(BL55100); *Dronc*^{KO} Tub-*G80*^{ts} (BL7019)/ +)<br><br>**Experimental without Dronc expression:**<br>*109-30-Gal4* (BL7023)/UAS-FUCCI(BL55100); *Dronc*^{KO} Tub-*G80*^{ts} (BL7019) / UAS-*flippase* (BL8209) *Dronc*$^{KO-FRT-Dronc-GFP-APEX-FRT-suntagHA}$)
**3A-C**	**Control:**<br>*109-30-Gal4* (BL7023)/+<br><br>**Experimental without Dronc expression:**<br>*109-30-Gal4* (BL7023)/+; *Dronc*^{KO} Tub-*G80*^{ts} (BL7019) / UAS-*flippase* (BL8209) *Dronc*$^{KO-}$ FRT Dronc-GFP-Apex FRT-Suntag-HA-Cherry<br><br>**Experimental expressing a catalytically inactive form of Dronc:**<br>*109-30-Gal4* (BL7023)/+; *Dronc*^{KO} Tub-*G80*^{ts} (BL7019) / UAS-*flippase* (BL8209) *Dronc*$^{KO-}$ FRT Dronc-GFP-Apex FRT-Dronc FL-CAEA-Suntag-HA-Cherry
**3D-F**	**Control:**<br>*109-30-Gal4*/+; Tub-*G80*^{ts} (BL7019)/+<br><br>**Experimental:**<br>*109-30-Gal4* (BL7023)/UAS-*Drice*RNAi UAS-*Decay*RNAi (a gift from Pascal Meier); UAS-*Damm*RNAi, UAS-*Dcp1*RNAi (a gift from Pascal Meier)<br><br>*109-30-Gal4* (BL7023)/+; Tub-*G80*^{ts} (BL7019)/ UAS-*Dcp1*RNAi (BL28909)<br><br>*109-30-Gal4* (BL7023)/+; Tub-*G80*^{ts} (BL7019)/ UAS-*Drice*RNAi (BL32403)<br><br>*109-30-Gal4* (BL7023)/UAS-*Dark-sh*; Tub-*G80*^{ts} (BL7019)/+ (a gift from M. Miura)<br><br>*109-30-Gal4* (BL7023)/UAS-microRNA-RHG; Tub-*G80*^{ts} (BL7019)/+ (a gift from Istwar Hariharan)
**4A-C**	**Control:**<br>*109-30-Gal4* (BL7023)*/ptc*-GFP^{CB02030} (a gift from Isabel Guerrero)<br><br>**Experimental:**<br>*109-30-Gal4* (BL7023)*/ptc*-GFP^{CB02030}*;* *Dronc*^{KO}Tub-*G80*^{ts} (BL7019)/ UAS-*flippase* *Dronc*$^{KO-FRT-Dronc-GFP-APEX-FRT-QF}$
**4F-G**	**Control:**<br>*109-30-Gal4* (BL7023)/UAS-*smo*^{Act} (BL44621); *Dronc*^{KO}Tub-*G80*^{ts} (BL7019)/+<br><br>**Experimental:**<br>*109-30-Gal4* (BL7023)/UAS-*smo*^{Act} (BL44621); *Dronc*^{KO}Tub-*G80*^{ts} (BL7019)/ UAS-*flippase* (BL8209) *Dronc*$^{KO-FRT-Dronc-GFP-APEX-FRT-QF}$

**4H, 4I**	*Dronc -/- = CTRL= 109-30-Gal4* (BL7023)/; *Dronc*^{KO}Tub-*G80*^{ts} (BL7019)/ UAS-*flippase* (BL8209) *Dronc*$^{KO\text{-}FRT\text{-}Dronc\text{-}GFP\text{-}APEX\text{-}FRT\text{-}QF}$<br><br>*CTRL= 109-30-Gal4* (BL7023)/UAS-*smo*^{Act} (BL44621); *Dronc*^{KO}Tub-*G80*^{ts} (BL7019)/+<br><br>*Dronc -/- = 109-30-Gal4* (BL7023)/UAS-*smo*^{Act} (BL44621); *Dronc*^{KO}Tub-*G80*^{ts} (BL7019)/ UAS-*flippase* (BL8209) *Dronc*$^{KO\text{-}FRT\text{-}Dronc\text{-}GFP\text{-}APEX\text{-}FRT\text{-}QF}$<br><br>*CTRL= 109-30-Gal4* (BL7023)/UAS-*Ci* (BL28984); *Dronc*^{KO}Tub-*G80*^{ts} (BL7019)/+<br><br>*Dronc -/- = 109-30-Gal4* (BL7023)/UAS- UAS-*Ci* (BL28984); *Dronc*^{KO}Tub-*G80*^{ts} (BL7019)/ UAS-*flippase* (BL8209) *Dronc*$^{KO\text{-}FRT\text{-}Dronc\text{-}GFP\text{-}APEX\text{-}FRT\text{-}QF}$
**4J, 4K**	*Dronc +/+ = ptc-Gal4* (BL2017)/+; Tub-*G80*^{ts} (BL7019)<br><br>*Dronc +/- = ptc-Gal4* (BL2017)/+; *Dronc*^{KO}Tub-*G80*^{ts} (BL7019)/+<br><br>*Dronc +/- UAS-Dronc = ptc-Gal4* (BL2017)/+; *Dronc*^{KO}Tub-*G80*^{ts} (BL7019)/UAS-*Dronc* (BL56198)
**5A-C**	*Dronc*^{KO}Tub-*G80*^{ts} (BL7019)/+<br><br>*ptc-Gal4* (BL2017)/+; Tub-*G80*^{ts} (BL7019)/+<br><br>*ptc-Gal4* (BL2017)/+; *Dronc*^{KO}Tub-*G80*^{ts} (BL7019)/+
**5D-G**	**Control:**<br>*ptc-Gal4* (BL2017)/UAS-*GFP-mCherry-Atg8* (BL37749)<br><br>**Experimental:**<br>*ptc-Gal4* (BL2017)/UAS-*GFP-mCherry-Atg8* (BL37749); *Dronc*^{KO}Tub-*G80*^{ts} (BL7019)/+
**5H-I**	*CTRL = 109-30*-Gal4 (BL7023)/+; *Dronc*^{KO}Tub-*G80*^{ts} (BL7019)/+<br><br>*Dronc -/- = 109-30Gal4* (BL7023)/+; *Dronc*^{KO}Tub-*G80*^{ts} (BL7019)/ UAS-*flippase* (BL8209) *Dronc*$^{KO\text{-}FRT\text{-}Dronc\text{-}GFP\text{-}APEX\text{-}FRT\text{-}QF}$<br><br>*Dronc -/-* UAS-*ptc*-RNAi = *109-30Gal4* (BL7023)/UAS-*ptc*-RNAi (BL55686); *Dronc*^{KO}Tub-*G80*^{ts} (BL7019)/ UAS-*flippase* (BL8209) *Dronc*$^{KO\text{-}FRT\text{-}Dronc\text{-}GFP\text{-}APEX\text{-}FRT\text{-}QF}$
**6A-D**	**Control:**<br>*ptc-Gal4* (BL2017)/ UAS-*GFP-mCherry-Atg8* (BL37749)<br><br>**Experimental:**<br>*ptc-Gal4* (BL2017)/ UAS-*GFP-mCherry-Atg8* (BL37749); *Dronc*^{KO}Tub-*G80*^{ts} (BL7019)/+

**6E-G**	*Dronc*^{KO}Tub-*G80*^{ts} (BL7019)/+<br><br>*ptc-Gal4* (BL2017)/+; Tub-*G80*^{ts} (BL7019)/+<br><br>*ptc-Gal4* (BL2017)/+; *Dronc*^{KO}Tub-*G80*^{ts} (BL7019)/+
**6J-L**	*ptc-Gal4* /+ = *ptc-Gal4* (BL2017)/+; Tub-*G80*^{ts} (BL7019)/+<br><br>*ptc-Gal4* /*Atg8*i = *ptc-Gal4* (BL2017)/UAS-*Atg8*RNAi (VDRC 109654); Tub-*G80*^{ts} (BL7019)/+<br><br>*ptc-Gal4* /*Atg8*i *Dronc*^{KO}/*Dronc*$^{KO\text{-suntag-HA-Cherry}}$= *ptc-Gal4* (BL2017)/UAS-*Atg8*RNAi (VDRC 109654); *Dronc*^{KO}Tub-*G80*^{ts} (BL7019)/ *Dronc*$^{KO\text{-FRT-Dronc-APEX-GFP-FRT-suntag-HA-Cherry}}$

Appendix Table S1. Description of fly lines and genotypes used in the experiments of the main Figures.

Genotypes and fly lines information	
**EV Figure**	**Genotype**
**EV 1B**	Actin *DBS-S-QF*, UAS-*mCD8-GFP*, QUAS-*tomato-HA*/+; QUAS-*flippase* (BL30126)/+; Actin5C FRT-*stop*-FRT *lacZ*-nls/+ (BL6355)
**EV 1C**	w;; *Dronc*$^{KO\text{-}Gal4}$ / UAS-*Histone-RFP* (BL56555)
**EV 1D**	*Dronc::*V5::TurboID/+ (a gift from Masayuki Miura)
**EV 1E-F**	*yw hs-flippase*$^{1.22}$/+; FRT80 *Dronc*^{I29}/ FRT80 Ubi*GFP*
**EV 2D**	*109-30*-Gal4 (BL7023)/+; UAS-*Histone-RFP* (BL56555) Tub-*G80*^{ts} (BL7019)/+
**EV 2E**	*109-30*-Gal4 (BL7023)/QUAS-*CD8-GFP* (BL 30002); *Dronc*^{KO} Tub-*G80*^{ts} (BL7019) / UAS-*flipasse* (BL8209) *Dronc*$^{KO\text{-}FRT\text{-}Dronc\text{-}GFP\text{-}APEX\text{-}FRT\text{-}QF}$
**EV 2F, 2G**	*109-30*-Gal4 (BL7023)/QUAS-*CD8-GFP* (BL 30002); *Dronc*^{KO} Tub-G80^{ts} (BL7019) / TM6b
**EV 2H**	*109-30*-Gal4 (BL7023)/QUAS-*CD8-GFP* (BL 30002); *Dronc*^{KO} Tub-*G80*^{ts} (BL7019) / UAS-*flipasse* (BL8209) *Dronc*$^{KO\text{-}FRT\text{-}Dronc\text{-}GFP\text{-}APEX\text{-}FRT\text{-}QF}$
**EV 2J**	*ptc-Gal4* (BL2017)/+; UAS-*Histone-RFP* (BL56555) Tub-*G80*^{ts} (BL7019)/+
**EV 2K**	*ptc-Gal4* (BL2017)/QUAS-*CD8-GFP* (BL 30002); *Dronc*^{KO} Tub-*G80*^{ts} (BL7019) / UAS-*Flipasse* (BL8209) *Dronc*$^{KO\text{-}FRT\text{-}Dronc\text{-}GFP\text{-}APEX\text{-}FRT\text{-}QF}$
**EV 2L**	**Control:**<br>*ptc-Gal4* (BL2017)/+; Tub-*G80*^{ts} (BL7019)/+<br><br>**Experimental:**<br>*ptc-Gal4* (BL2017)/+; *Dronc*^{KO} Tub-*G80*^{ts} (BL7019) / UAS-*flippase* (BL8209) *Dronc*$^{KO\text{-}FRT\text{-}Dronc\text{-}GFP\text{-}APEX\text{-}FRT\text{-}QF}$
**EV 3B, 3C**	*ptc-Gal4* (BL2017)<br>*ptc-Gal4* (BL2017)/UAS-*Diap1* (BL63819)<br>*ptc-Gal4* (BL2017)/UAS-P35 (BL5072); UAS-P35 (BL5073)/+
**EV 4B**	*109-30*-Gal4 (BL7023)/+
**EV 4C**	*109-30*-Gal4 (BL7023)/+; UAS-*Ci*-RNAi (BL28984)/+
**EV 4D, 4E**	CTRL= *109-30*-Gal4 (BL7023)/+<br><br>*Dronc -/-* =*109-30*-Gal4 (BL7023)/+; UAS-*Ci*-RNAi (BL28984)/+<br><br>CTRL = *ptc-Gal4* (BL2017)/ + ; Tub-*G80*^{ts} (BL7019)/+<br><br>UAS-*ptc*^{1130X}YFP = *ptc-Gal4* (BL2017)/ UAS-*ptc*^{1130X}YFP (BL52215); Tub-*G80*^{ts} (BL7019)/+
**EV 4F**	*109-30*-Gal4 (BL7023)/UAS-*Ci* (BL32571); *Dronc*^{KO} Tub-*G80*^{ts} (BL7019) / +
**EV 4G**	*109-30*Gal4 (BL7023)/UAS-*Ci* (BL32571); *Dronc*^{KO} Tub-*G80*^{ts} (BL7019)/ UAS-*Flipasse* (BL8209) *Dronc*$^{KO\text{-}FRT\text{-}Dronc\text{-}GFP\text{-}APEX\text{-}FRT\text{-}QF}$
**EV 4H**	*ptc-Gal4* (BL2017)/UAS-*hh-EGFP.H* (BL81024); Tub-*G80*^{ts} (BL7019) / +

**EV 4I**	*ptc-Gal4* (BL2017)/ UAS-*hh-EGFP.H* (BL81024); *Dronc*^{KO} Tub-*G80*^{ts} (BL7019)/ UAS-*Flipasse* (BL8209) *Dronc*$^{KO\text{-}FRT\text{-}Dronc\text{-}GFP\text{-}APEX\text{-}FRT\text{-}suntag\text{-}HA\text{-}Cherry}$
**EV 4J**	**Control:**<br>*ptc-Gal4* (BL2017)/UAS-*hh-EGFP.H* (BL81024); Tub-*G80*^{ts} (BL7019) / +<br><br>**Experimental:**<br>*ptc-Gal4* (BL2017)/ UAS-*hh-EGFP.H* (BL81024); *Dronc*^{KO} Tub-*G80*^{ts} (BL7019)/ UAS-*Flipasse* (BL8209) *Dronc*$^{KO\text{-}FRT\text{-}Dronc\text{-}GFP\text{-}APEX\text{-}FRT\text{-}suntag\text{-}HA\text{-}Cherry}$.
**EV 4L**	*ptc*-GFP^{CB02030} (a gift from Isabel Guerrero) /+; *Dronc*^{KO}Tub-*G80*^{ts} (BL7019)/+
**EV 4M**	*ptc*^{S2}*(BL6332)/+; Dronc*^{KO}/+
**EV 4N**	**Control:**<br>*ptc*^{S2}*(BL6332)/+*<br>**Experimental:**<br>*ptc*^{S2}*(BL6332)/+; Dronc*^{KO}/+
**EV 5A**	*ptc-Gal4* (BL2017)/UAS-*mCD8-GFP (BL108068)*; *Dronc*^{KO} Tub-*G80*^{ts} (BL7019)/+
**EV 5B**	*Dronc*^{KO}/+<br>*ptc-Gal4* (BL2017)/+<br>*ptc-Gal4* (BL2017)/+; *Dronc*^{KO} Tub-*G80*^{ts} (BL7019) /+
**EV 5C**	*ptc-Gal4* (BL2017)/ UAS-*ptc*^{1130X}YFP (BL52215); Tub-*G80*^{ts} (BL7019)
**EV 5D**	*ptc-Gal4* (BL2017)/ UAS-*ptc*^{1130X}YFP (BL52215); *Dronc*^{KO} Tub-*G80*^{ts} (BL7019) /+
**EV 5E**	**Control:**<br>CTRL = *ptc-Gal4* (BL2017)/ UAS-*ptc*^{1130X} YFP (BL52215); Tub-*G80*^{ts} (BL7019). (n=8)<br>**Experimental:**<br>*Dronc +/- = ptc-Gal4* (BL2017)/ UAS-*ptc*^{1130X}YFP (BL52215); *Dronc*^{KO} Tub-*G80*^{ts} (BL7019) /+
**EV 5F**	*ptc-Gal4* (BL2017)/ *ptc*-GFP^{CB02030}; *Dronc*^{KO} Tub-*G80*^{ts} (BL7019) / +
**Appendix Fig S4B-D**	*ptc-Gal4* (BL2017)/+; *Dronc*^{KO}Tub-*G80*^{ts} (BL7019)/+<br>*ptc-Gal4* (BL2017)/+; Tub-*G80*^{ts} (BL7019)/UAS-*Atg1*-RNAi (BL35177)<br>*ptc-Gal4* (BL2017)/+; *Dronc*^{KO}Tub-*G80*^{ts} (BL7019)/UAS-*Atg1*-RNAi (BL35177)

Appendix Table S2. Description of fly lines and genotypes used in the experiments of Expanded View and Appendix Figures.

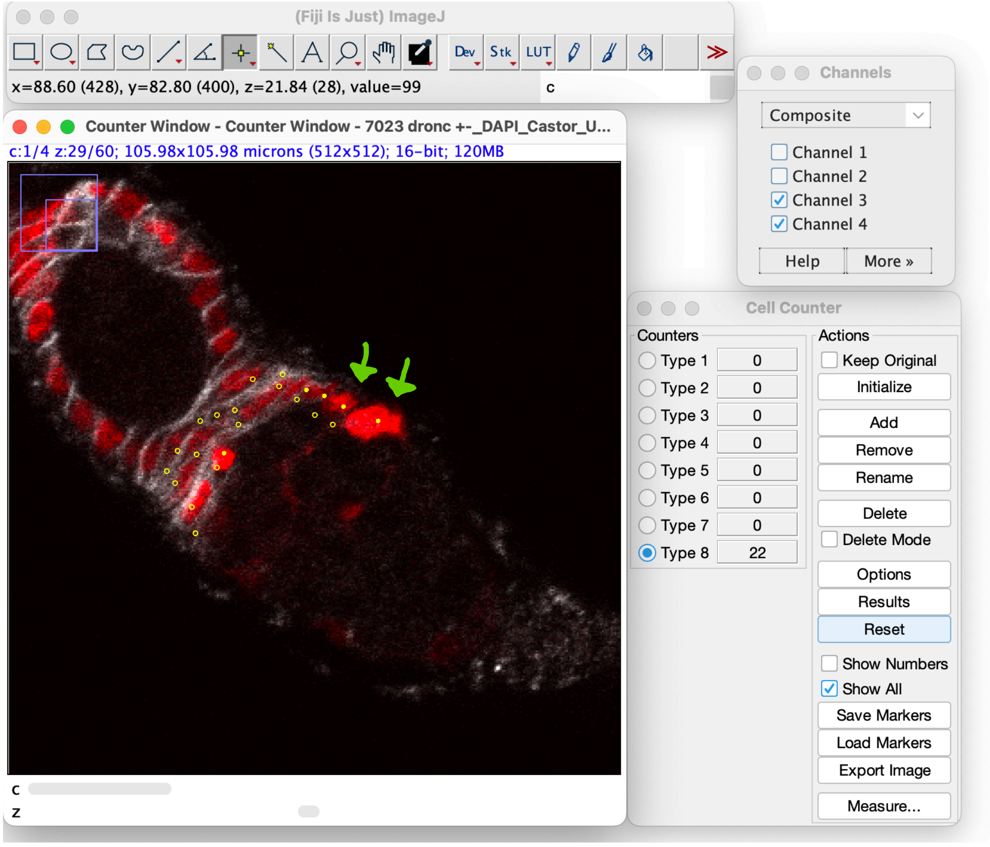

Focal plane 29

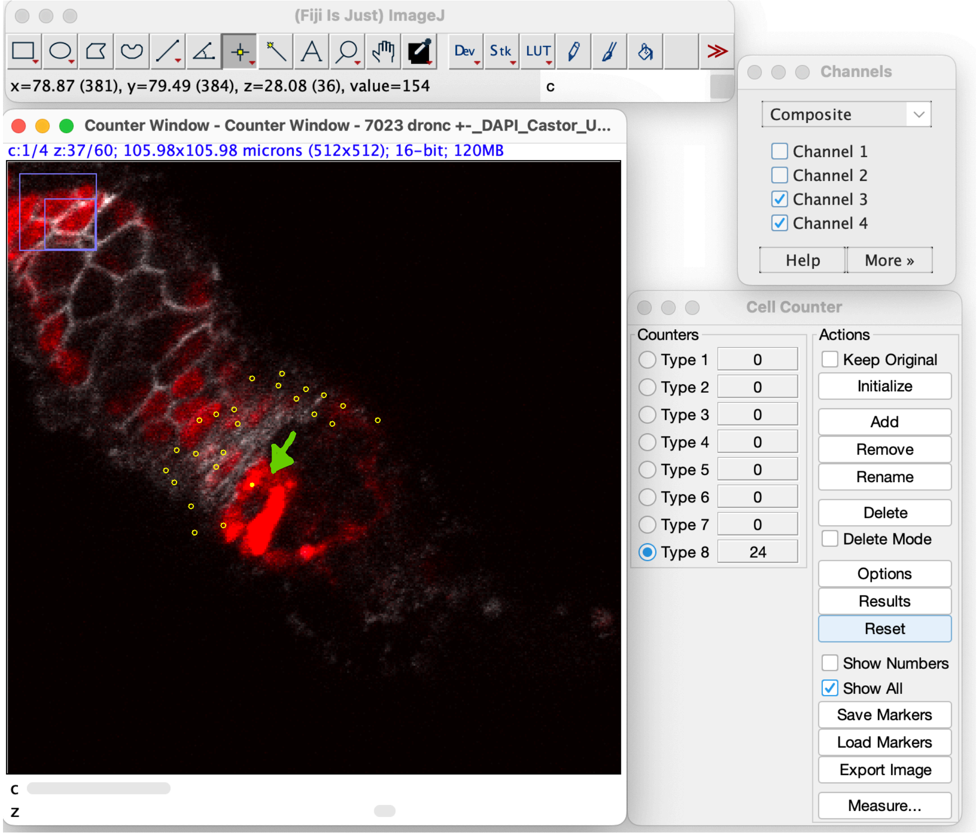

Focal plane 37

Appendix Figure S2. Figure describing the counting methodology used for follicular cells throughout the manuscript. To permanently label cells expressing the nuclear marker Histone-RFP (this is specific of the sample shown but we also used other markers to recognise follicular cells), we employ the Cell Counter plug-in in the Fiji software. Specifically, cells identified in a given focal plane, such as focal plane 29, are manually marked with a solid dot (some examples are indicated by the green arrowhead) while the counter simultaneously displays the number of dots generated. This process is repeated for each confocal plane in the Z-stack. Notably, in subsequent focal planes, such as focal plane 37, the position of previously labelled cells is displayed with an empty circle while new cells are marked with solid dots. This approach ensures accurate cell counting of follicular cells throughout the entire Z-stack.
