# Supplementary material for: Non‐apoptotic caspase activation ensures the homeostasis of ovarian somatic stem cells
Source: EMBO Rep. 2023 Apr 11;24(6):e51716. doi: 10.15252/embr.202051716 (PMC10240206; doi:10.15252/embr.202051716)
Supplement: Supplementary file 2 — Expanded View Figures PDF [file EMBR-24-e51716-s001.pdf]

Expanded View Figures

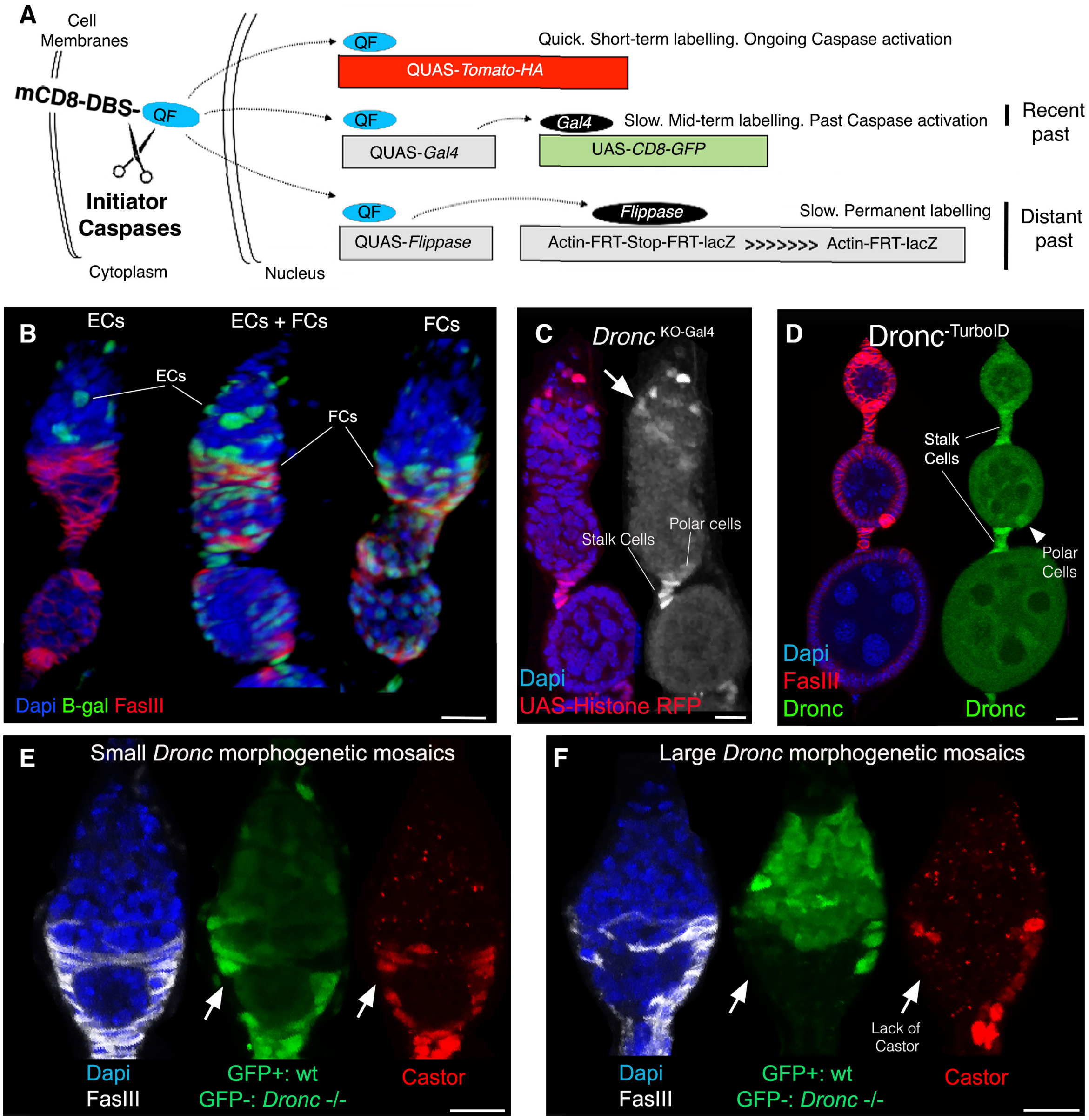

Figure EV1.

Figure EV1. Non-apoptotic caspase activation patterns detected in the germarium are connected to Dronc.

A Schematic diagram illustrating the temporal caspase activation profile obtained using Drice-based sensor (DBS-S-QF). Left: schematic representation of the membrane attached mCD8-DBS-QF sensor. Right: different labelling systems used in combination with mCD8-DBS-QF to visualise the temporal patterns of initiator caspase activation. The early and ongoing view of initiator caspase activation is obtained through the expression of QUAS-*tomato-HA*. In parallel to the expression of tomato-HA, the QF translocation into the nucleus upon caspase-mediated cleavage can also activate the expression of the Gal4 transcription factor (QUAS-*Gal4*) and the recombinase known as Flippase (QUAS-*flippase*). Gal4 production can subsequently promote the transcription of a second cellular marker (UAS-*CD8-GFP*). Notice that the appearance of the GFP is temporarily delayed with respect to the tomato-HA signal since it demands a second transcriptional step. However, the GFP signal lasts for longer than the tomato-HA upon the caspase-mediated release of QF ceases due to the second transcriptional amplification loop obtained with the Gal/UAS system. Permanent labelling of caspase-activating cells (lineage tracing) can be achieved with the production of the Flippase recombinase. This enzyme facilitates the genomic excision of an FRT-stop cassette that prevents the expression of a nuclear β-galactosidase (β-gal) from the *lacZ* gene under the control of the actin promoter. Upon cassette excision, the production of β-gal stays forever in cells alive (permanent labelling).

B Representative 3D projections of confocal images showing the permanent labelling of different regions in the germarium (green, β-gal) obtained with DBS-S-QF. Nuclei are labelled with DAPI staining (blue). FasIII immunostaining labels the somatic follicular cells (red). Genotype: Actin *DBS-S-QF*, UAS-*mCD8-GFP*, QUAS-*tomato-HA*/+; QUAS-*flippase* (BL30126)/+; Actin5C FRT-*stop*-FRT *lacZ*-nls/+ (BL6355).

C Representative 2D projection of confocal images showing the expression pattern of *Dronc*^{KO}-*Gal4* at 29°C 10 days after adult eclosion from the pupae. UAS-*Histone-RFP* reports on Gal4 transcription (red and grey). DAPI stains the nuclei (blue). Notice the expression of *Dronc* in somatic cells of the germarium (arrows) as well as germarium the stalk and polar cells. Genotype: w;; *Dronc*$^{KO\text{-}Gal4}$/UAS-*Histone-RFP* (BL56555).

D Biotinylation signal (green) generated in the germarium by a *Dronc*-TurboID allele; notice the signal enrichment in stalk cells (white arrows) and polar cells (white arrowhead). FasIII (red) and DAPI (blue) stainings label the somatic cells and the nuclei, respectively. Experimental flies were kept after eclosion from the pupae for 10 days at 29°C prior to dissection. Genotype: *Dronc*::V5::TurboID/+ (a gift from Masayuki Miura).

E, F Expression of Castor (red) and FasIII (grey) in mutant morphogenetic mosaics for *Dronc*^{I29} in the germarium (GFP negative cells). Notice the downregulation of Castor (red, white arrows). Genotype: *yw hs-flippase*$^{1.22}$/+; FRT80 *Dronc*^{I29}/FRT80 Ubi*GFP*.

Data information: Scale bars represent 10 μm in the entire figure. Full description of genotypes for all of the EV Figures can be found in Appendix Table S2.

 © 2023 The Authors

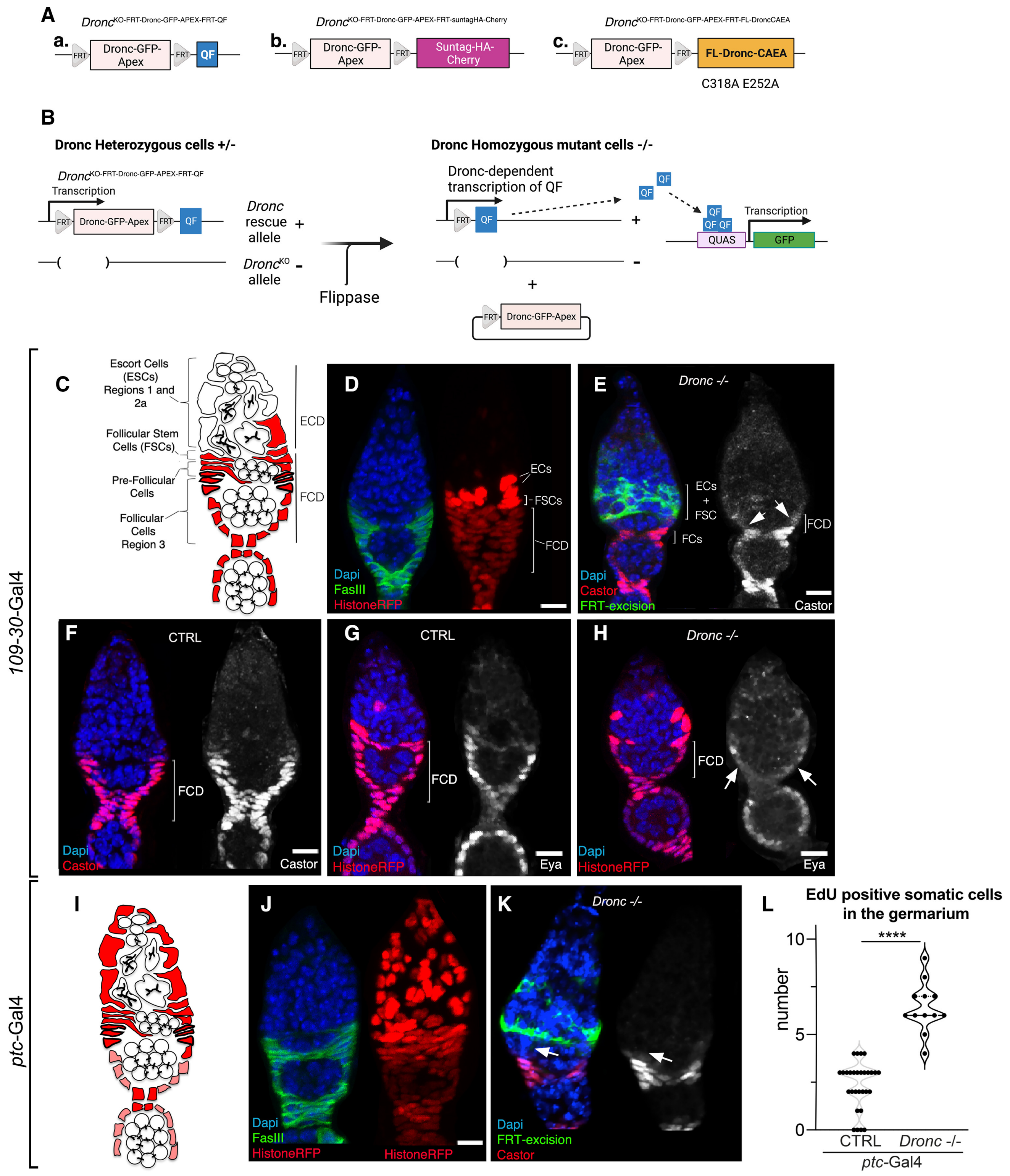

A
a. Dronc-GFP-Apex FRT QF
b. Dronc-GFP-Apex FRT Suntag-HA-Cherry
c. Dronc-GFP-Apex FRT FL-Dronc-CAEA
C318A E252A
B
Dronc Heterozygous cells +/-
Dronc Homozygous mutant cells -/-
Transcription
Dronc rescue allele
Dronc allele
Flippase
Dronc-dependent transcription of QF
QUAS
GFP
C
Escort Cells (ESCs) Regions 1 and 2a
Follicular Stem Cells (FSCs)
Pre-Follicular Cells
Follicular Cells Region 3
ECD
FCD
D
ECs
FSCs
Dapi
FasIII
HistoneRFP
E
Dronc -/-
ECs + FSC
FCs
Castor
FRT-excision
F
CTRL
G
Eya
H
109-30-Gal4
ptc-Gal4
I
J
K
L
EdU positive somatic cells in the germarium
number
****
CTRL
Dronc -/-

Figure EV2.

Figure EV2. Expression pattern of relevant Gal4 drivers used in the manuscript.

A Schematics depicting the conditional *Dronc* alleles used throughout the manuscript. (a) the *Dronc*$^{KO\text{-}FRT\text{-}Dronc\text{-}GFP\text{-}ApexFRT\text{-}QF}$ allele expresses the transcriptional activator QF under the physiological regulation of *Dronc* upon FRT-rescue cassette excision; (b) the *Dronc*$^{KO\text{-}FRT\text{-}Dronc\text{-}GFP\text{-}ApexFRT\text{-}suntagHA\text{-}Cherry}$ allele expresses a chimeric Suntag-HA-Cherry polypeptide upon FRT-rescue cassette excision; (c) the *Dronc*$^{KO\text{-}FRT\text{-}Dronc\text{-}GFP\text{-}ApexFRT\text{-}FL\text{-}DroncCAEA}$ allele can express a full-length version of Dronc that has mutated the cysteine in the catalytic pocket C318A and the glutamic acid required for Dronc activation E352A. Diagram generated with BioRender.

B Diagram illustrating the allele conversion of Dronc heterozygous cells into homozygous mutants using the allele *Dronc*$^{KO\text{-}FRT\text{-}Dronc\text{-}GFP\text{-}ApexFRT\text{-}QF}$. Notice the Dronc-QF-dependent induction of a QUAS-CD8-GFP transgene upon Flippase-mediated excision of the FRT-rescue cassette. Diagram generated with BioRender.

C Schematic depicting the lineage tracing of *109-30*-Gal4 expressing cells (coloured in red) in the germarium at 29°C. Follicular Stem Cells (FSCs), pre-follicular cells (Pre-FCs) and adjacent Escort cells (ECs) are indicated.

D Representative 2D projection of confocal images showing a germarium expressing UAS-*Histone-RFP* (red) under the regulation of *109-30*-Gal4 driver during 14 days at 29°C; Castor (green) and DAPI (blue) label the follicular cells and the nuclei, respectively. Genotype: *109-30*-Gal4 (BL7023)/+; UAS-*Histone-RFP* (BL56555) Tub-*G80*^{ts} (BL7019)/+.

E Castor expression (red, grey and white arrows) in follicular cells without Dronc (green). Genotype 109-30-Gal4/QUAS-CD8-GFP; *Dronc*^{KO} Tub-G80^{ts}/UAS-*flippase* *Dronc*$^{KO\text{-}FRT\text{-}Dronc\text{-}GFP\text{-}APEX\text{-}FRT\text{-}QF}$. *Dronc*-expressing cells excising the rescue cassette are labelled with GFP (green); notice the reduction in number of Castor-expressing cells and size of the FCD (white arrows). Genotype: *Dronc*−/−: *109-30*-Gal4 (BL7023)/QUAS-*CD8-GFP* (BL 30002); *Dronc*^{KO} Tub-*G80*^{ts} (BL7019)/UAS-*flipasse* (BL8209) *Dronc*$^{KO\text{-}FRT\text{-}Dronc\text{-}GFP\text{-}APEX\text{-}FRT\text{-}QF}$.

F Wild-type expression of the follicular marker Castor (red and/or grey) in a representative germarium of the following genotype: CTRL: *109-30*-Gal4 (BL7023)/QUAS-*CD8-GFP* (BL 30002); *Dronc*^{KO} Tub-G80^{ts} (BL7019)/TM6b.

G Wild-type expression of the follicular marker Eyes absent (Eya) (red and/or grey) in a representative germarium of the following genotype: CTRL: *109-30*-Gal4 (BL7023)/QUAS-*CD8-GFP* (BL 30002); *Dronc*^{KO} Tub-G80^{ts} (BL7019)/TM6b.

H Eya expression (red, grey and white arrows) in follicular cells without Dronc. Notice the reduced expression of Eya (white arrows). Genotype: *Dronc*−/−: *109-30*-Gal4 (BL7023)/QUAS-*CD8-GFP* (BL 30002); *Dronc*^{KO} Tub-*G80*^{ts} (BL7019)/UAS-*flipasse* (BL8209) *Dronc*$^{KO\text{-}FRT\text{-}Dronc\text{-}GFP\text{-}APEX\text{-}FRT\text{-}QF}$.

I Schematic depicting the lineage tracing of *ptc-Gal4* expressing cells (coloured in red) in the germarium at 29°C.

J Representative 2D projection of confocal images showing a germarium expressing UAS-*Histone-RFP* (red) under the regulation of *ptc-Gal4* driver at 29°C. FasIII (green) and DAPI (blue) label the follicular cells and the nuclei of the germarium, respectively. Genotype: *ptc-Gal4* (BL2017)/+; UAS-*Histone-RFP* (BL56555) Tub-*G80*^{ts} (BL7019)/+.

K Castor expression (red, grey and white arrows) in a representative *Dronc* mutant germarium of the following genotype *ptc-Gal4* (BL2017)/QUAS-*CD8-GFP* (BL 30002); *Dronc*^{KO} Tub-*G80*^{ts} (BL7019)/UAS-*Flipasse* (BL8209) *Dronc*$^{KO\text{-}FRT\text{-}Dronc\text{-}GFP\text{-}APEX\text{-}FRT\text{-}QF}$. *Dronc*-expressing cells excising the rescue cassette are labelled with GFP (green); notice the reduction in number of Castor-expressing cells in the FCD as well as the size (white arrows).

L Quantification of somatic cells in S-phase labelled by EdU incorporation in control (CTRL: *ptc-Gal4* (BL2017)/+; Tub-*G80*^{ts} (BL7019)/+; n = 30) versus *Dronc* mutant germaria (*Dronc*−/−: *ptc-Gal4* (BL2017)/+; *Dronc*^{KO} Tub-*G80*^{ts} (BL7019)/UAS-*flippase* (BL8209) *Dronc*$^{KO\text{-}FRT\text{-}Dronc\text{-}GFP\text{-}APEX\text{-}FRT\text{-}QF}$; n = 23). Statistical significance was determined using an unpaired parametric Welch's t-test (****$P \leq 0.0001$).

Data information: Scale bars represent 10 μm in the entire figure. Experimental flies were kept after eclosion from the pupae for 14 days at 29°C prior to dissection. All the experimental data shown have been obtained from $N \geq 2$ biological replicates. The median and quartiles are indicated in the violin plots. All the quantifications were made in germaria containing one single group of germline cells wrapped by follicular cells.
Source data are available online for this figure.

© 2023 The Authors

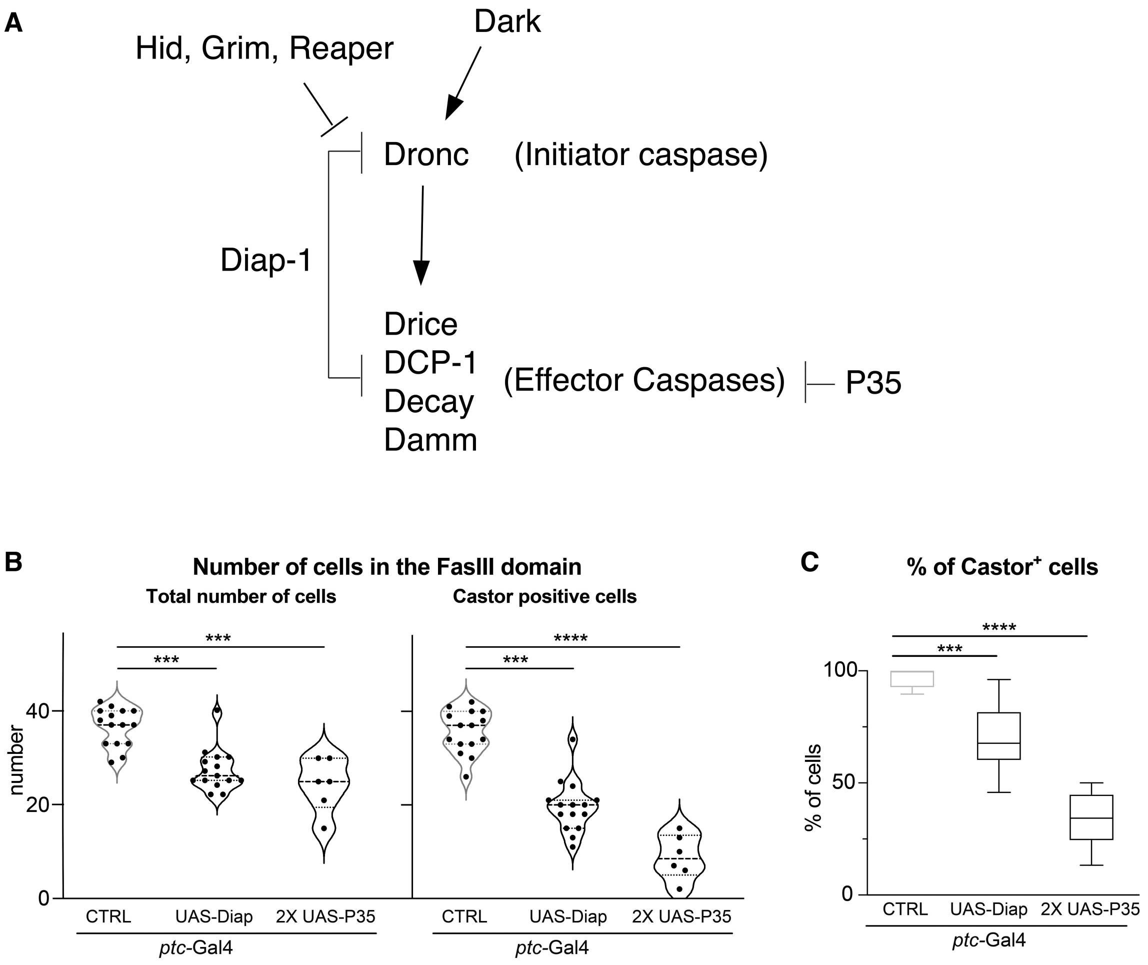
A
Dark
Hid, Grim, Reaper
Dronc (Initiator caspase)
Diap-1
Drice
DCP-1
Decay
Damm
(Effector Caspases)
P35
B
Number of cells in the FasIII domain
Total number of cells
Castor positive cells
number
CTRL UAS-Diap 2X UAS-P35
ptc-Gal4
C
% of Castor+ cells
% of cells
CTRL UAS-Diap 2X UAS-P35
ptc-Gal4

Figure EV3.

Figure EV3. Caspase inhibition alters the cellular properties of ovarian somatic cells.

A Schematic depicting the caspase pathway, upstream regulators of *Dronc* and specific inhibitors of caspase activation such as Diap-1 and P35.

B Quantification of total number of follicular cells (left) or Castor-expressing cells (right) within the FasIII cellular domain in the following genotypes from left to right: *ptc-Gal4* (BL2017) (N = 2; n = 15). *ptc-Gal4* (BL2017)/UAS-*Diap1* (BL63819) (N = 2; n = 15) *ptc-Gal4* (BL2017)/UAS-P35 (BL5072); UAS-P35 (BL5073)/+ (N = 1; n = 6). Statistical significance was established by using Kruskal–Wallis test and Dunn's multiple comparison post-test (***$P \leq 0.001$; ****$P \leq 0.0001$).

C Percentage of Castor-expressing cells versus the total number of Follicular cells ($FasIII^+$ cells) in germaria of the genotypes indicated in (B). n numbers are shown in (B). A Kruskal–Wallis test and Dunn's multiple comparison post-test were used to determine statistical significance (***$P \leq 0.001$; ****$P \leq 0.0001$).

Data information: Experimental flies were kept after eclosion from the pupae for 14 days at 29°C prior to dissection. The violin plots show the mean and standard deviations in the entire figure. The box plot shows the median, first quartile and third quartile of the dataset. The whiskers illustrate the range between the maximum and minimum values of the dataset. All the quantifications were made in germaria containing one single group of germline cells wrapped by follicular cells.
Source data are available online for this figure.

 © 2023 The Authors

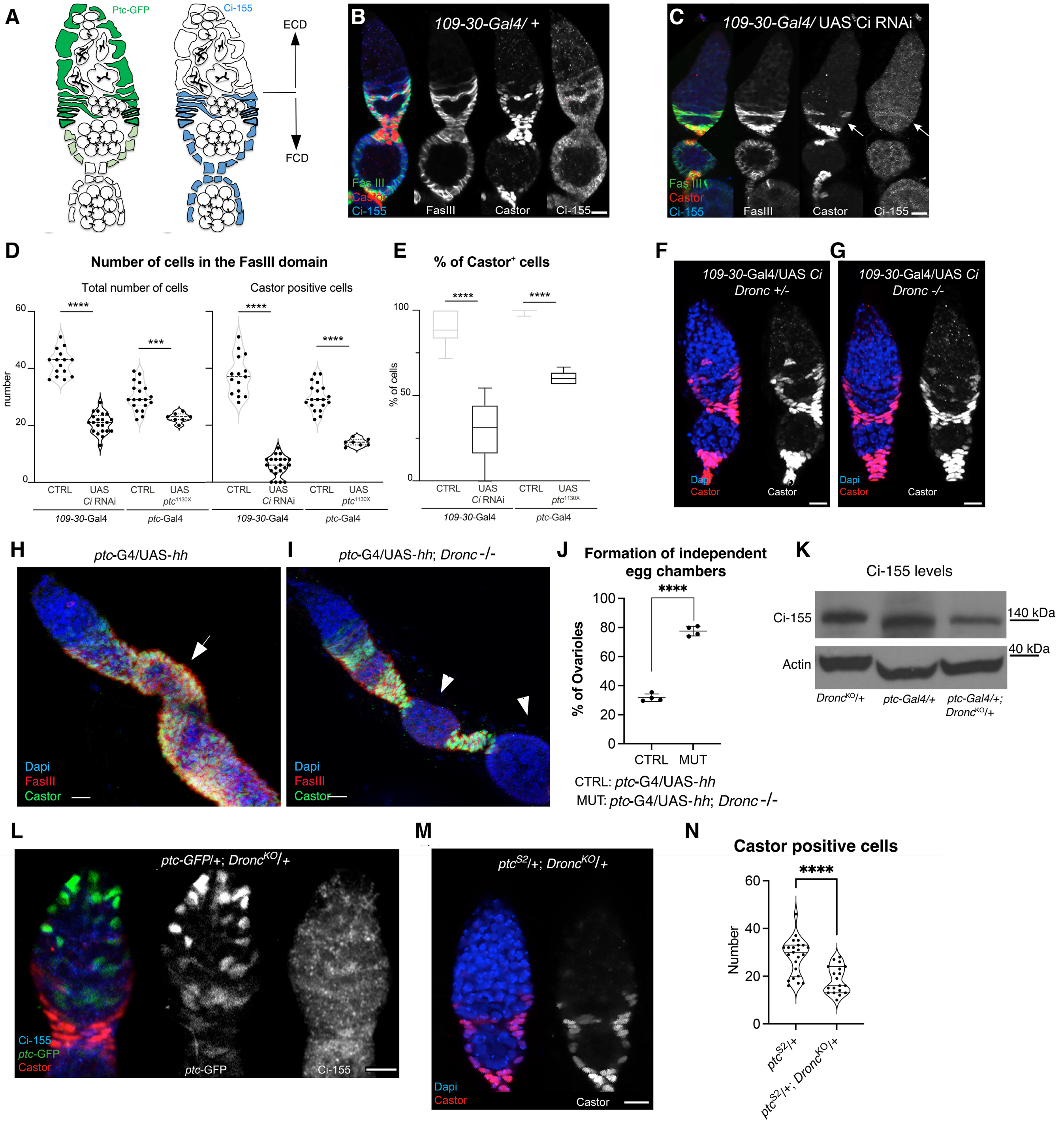
A
Ptc-GFP
Ci-155
ECD
FCD
B
109-30-Gal4/ +
Fas III
Castor
Ci-155
FasIII
Castor
Ci-155
C
109-30-Gal4/ UAS Ci RNAi
Fas III
Castor
Ci-155
FasIII
Castor
Ci-155
D
Number of cells in the FasIII domain
Total number of cells
Castor positive cells
number
CTRL
UAS Ci RNAi
CTRL
UAS ptc1130X
109-30-Gal4
ptc-Gal4
E
% of Castor+ cells
% of cells
F
109-30-Gal4/UAS Ci Dronc +/-
Dapi
Castor
Castor
G
109-30-Gal4/UAS Ci Dronc -/-
Dapi
Castor
Castor
H
ptc-G4/UAS-hh
Dapi
FasIII
Castor
I
ptc-G4/UAS-hh; Dronc -/-
Dapi
FasIII
Castor
J
Formation of independent egg chambers
% of Ovarioles
CTRL
MUT
CTRL: ptc-G4/UAS-hh
MUT: ptc-G4/UAS-hh; Dronc -/-
K
Ci-155 levels
Ci-155
Actin
140 kDa
40 kDa
DroncKO/+
ptc-Gal4/+
ptc-Gal4/+; DroncKO/+
L
ptc-GFP/+; DroncKO/+
Ci-155
ptc-GFP
Castor
ptc-GFP
Ci-155
M
ptcS2/+; DroncKO/+
Dapi
Castor
Castor
N
Castor positive cells
Number
ptcS2/+
ptcS2/+; DroncKO/+

Figure EV4.

Figure EV4. ***Dronc*** **deficiency limits Hh-signalling.**

A Schematics depicting the pattern of expression of ptc-GFP (green, left) and Ci-155 (blue, right) in the germarium. Notice the high levels of Ptc-GFP in Escort cells (ECs) and Follicular Stem Cells (FSCs) and the low levels in some follicular cells. High levels of Ci-155 are observed in follicular cells and the escort cells adjacent to the FSCs.

B Representative 2D projection of confocal images showing FasIII (green and/or grey), Castor (red and/or grey) and Ci-155 (blue and/or grey) expression in a control germarium (*109-30*-Gal4 (BL7023)/+).

C Representative 2D projection of confocal images showing FasIII (green and/or grey), Castor (red and/or grey) and Ci-155 (blue and/or grey) expression in a representative control germarium (*109-30*-Gal4 (BL7023)/+; UAS-*Ci*-RNAi (BL28984)/+). Notice the gaps in Castor expression within the Follicular region (white arrows).

D Quantification of total number of follicular cells (left) or Castor-expressing cells (right) within the FasIII cellular domain in the following genotypes from left to right: CTRL = *109-30*-Gal4 (BL7023)/+ (n = 15). *Dronc*−/− = *109-30*-Gal4 (BL7023)/+; UAS-*Ci*-RNAi (BL28984)/+ (n = 21). CTRL = *ptc-Gal4* (BL2017)/+; Tub-$G80^{ts}$ (BL7019)/+ (n = 19). UAS-ptc^{1130X}YFP = *ptc-Gal4* (BL2017)/UAS-ptc^{1130X}YFP (BL52215); Tub-$G80^{ts}$ (BL7019)/+ (n = 8). An unpaired parametric Welch's t-test was used to determine statistical significance (***$P \le 0.001$; ****$P \le 0.0001$).

E Percentage of Castor-expressing cells versus the total number of Follicular cells (FasIII^{+} cells) in germaria of the genotypes indicated in (D). n numbers are shown in (D). A nonparametric Mann–Whitney t-tests were used to determine statistical significance (****$P \le 0.0001$).

F, G Representative 2D projection of confocal images showing Castor expression (red and/or grey) and DAPI (blue) in mutant germarium of the following genotypes: *109-30*-Gal4 (BL7023)/UAS-*Ci* (BL32571); $Dronc^{KO}$ Tub-$G80^{ts}$ (BL7019)/+ (F). *109–30*Gal4 (BL7023)/UAS-*Ci* (BL32571); $Dronc^{KO}$ Tub-$G80^{ts}$ (BL7019)/UAS-*Flipasse* (BL8209) $Dronc^{KO\text{-}FRT\text{-}Dronc\text{-}GFP\text{-}}$APEX$^{\text{-}FRT\text{-}QF}$ (G).

H, I Representative 3D projections of confocal images showing Castor expression (green), FasIII (red) and DAPI (blue) in mutant germarium of the following genotypes: *ptc-Gal4* (BL2017)/UAS-*hh-EGFP.H* (BL81024); Tub-$G80^{ts}$ (BL7019)/+ (H). *ptc-Gal4* (BL2017)/UAS-*hh-EGFP.H* (BL81024); $Dronc^{KO}$ Tub-$G80^{ts}$ (BL7019)/UAS-*Flipasse* (BL8209) $Dronc^{KO\text{-}FRT\text{-}Dronc\text{-}GFP\text{-}APEX\text{-}FRT\text{-}suntag\text{-}HA\text{-}Cherry}$ (I). Whereas Castor expression is continuous and the egg chambers are normally fused in the genetic background of (H), caspase deficiency in (I) facilitates the formation of independent egg chambers and discontinuity in Castor expression (arrowheads).

J Frequency of ovarioles showing independent egg chambers and castor discontinuity in the genotypes shown in (H and I). *ptc-Gal4* (BL2017)/UAS-*hh-EGFP.H* (BL81024); Tub-$G80^{ts}$ (BL7019)/+. (N = 4, n = 445). *ptc-Gal4* (BL2017)/UAS-*hh-EGFP.H* (BL81024); $Dronc^{KO}$ Tub-$G80^{ts}$ (BL7019)/UAS-*Flipasse* (BL8209) $Dronc^{KO\text{-}FRT\text{-}Dronc\text{-}GFP\text{-}APEX\text{-}FRT\text{-}suntag\text{-}HA\text{-}Cherry}$. ($N$ = 4, n = 335). An unpaired parametric Welch's t-test was used to determine statistical significance (****$P \le 0.0001$).

K Western blot showing Ci-155 expression (upper lane) and actin (bottom lane, loading control) in the genotypes indicated in the picture. Notice the downregulation of Ci-155 72 h after siRNA treatment.

L Representative 2D projection of confocal images showing the expression of Ci-155 (blue and grey channels), *ptc*-GFP (green and grey) and Castor (red and grey) in *ptc*-GFP^{CB02030} (a gift from Isabel Guerrero)/+; $Dronc^{KO}$Tub-$G80^{ts}$ (BL7019)/+ germaria. Notice the downregulation of *ptc*-GFP and Ci-155 (compare with Fig 4A).

M Representative 2D projection of confocal images showing the expression of Castor (blue and grey) and DAPI (blue) in a ptc^{S2}*(BL6332)*/+; $Dronc^{KO}$/+ germarium.

N Quantification of Castor expression within the FasIII domain in the following genotypes: ptc^{S2}*(BL6332)*/+ (N = 2; n = 23). ptc^{S2}*(BL6332)*/+; $Dronc^{KO}$/+ (N = 2; n = 19). An unpaired parametric Welch's t-test was used to determine statistical significance (****$P \le 0.0001$).

Data information: Scale bars represent 10 μm in all of the confocal images of the figure. Experimental flies were kept after eclosion from the pupae for 14 days at 29°C prior to dissection. The median and quartiles are indicated in the violin plots. The box plots show the median, first quartile and third quartile of datasets. The whiskers illustrate the range between the maximum and minimum values of datasets. All the quantifications were made in germaria containing one single group of germline cells wrapped by follicular cells.

Source data are available online for this figure.

 © 2023 The Authors

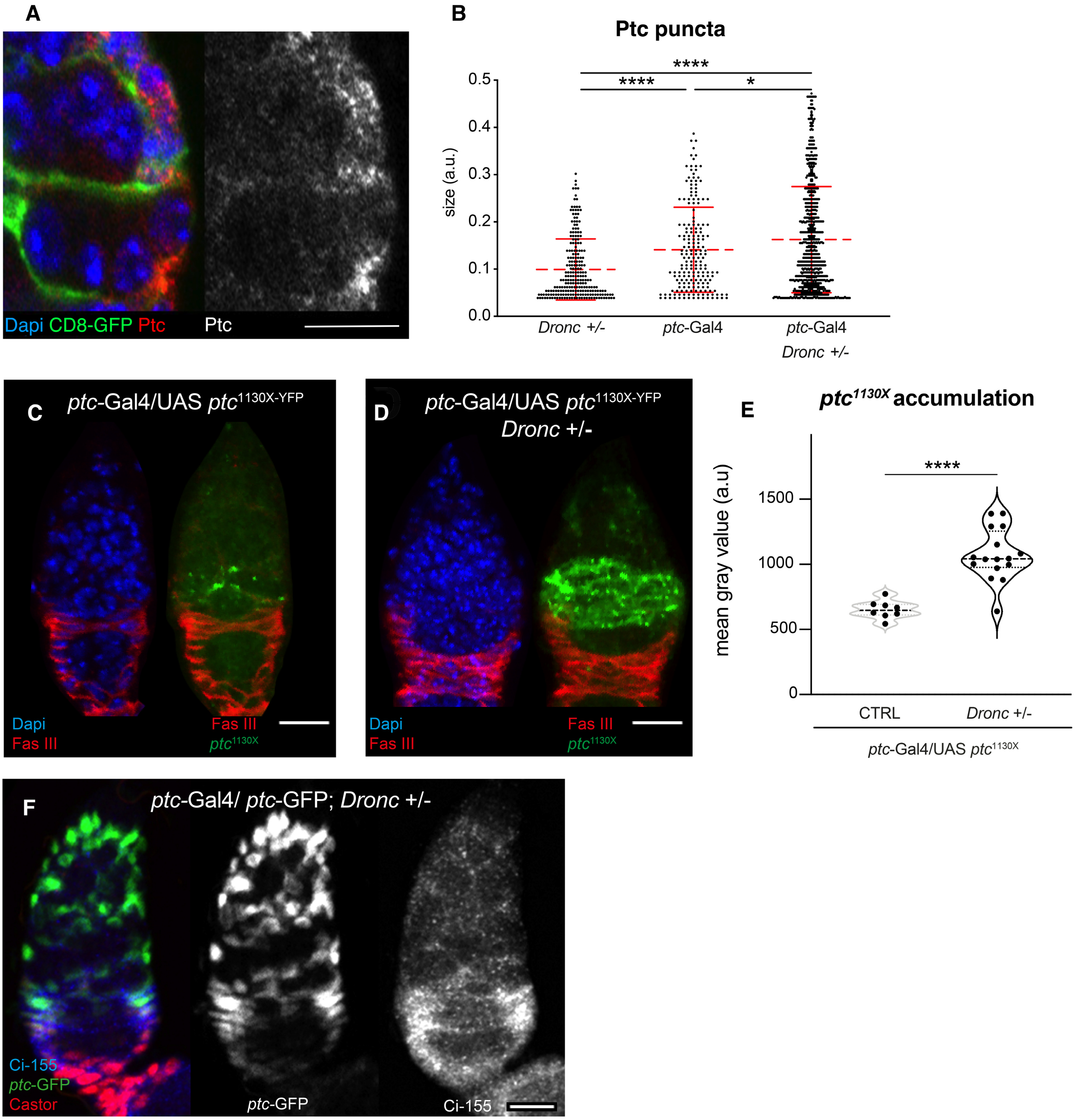
A
Dapi CD8-GFP Ptc
Ptc
B
Ptc puncta
size (a.u.)
0.0
0.1
0.2
0.3
0.4
0.5
****
****
*
Dronc +/-
ptc-Gal4
ptc-Gal4 Dronc +/-
C
ptc-Gal4/UAS ptc1130X-YFP
Dapi
Fas III
Fas III
ptc1130X
D
ptc-Gal4/UAS ptc1130X-YFP Dronc +/-
Dapi
Fas III
Fas III
ptc1130X
E
ptc1130X accumulation
mean gray value (a.u)
0
500
1000
1500
****
CTRL
Dronc +/-
ptc-Gal4/UAS ptc1130X
F
ptc-Gal4/ ptc-GFP; Dronc +/-
Ci-155
ptc-GFP
Castor
ptc-GFP
Ci-155

Figure EV5.

◀ **Figure EV5. *Dronc* deficiency facilitates the intracellular accumulation of Ptc.**

A Representative 2D projection of confocal images showing the expression of Ptc (red and grey), mCD8-GFP (green) and DAPI (blue) in a *ptc-Gal4* (BL2017)/UAS-*mCD8-GFP (BL108068)*; *Dronc*^{KO} Tub-*G80*^{ts} (BL7019)/+ germarium. Notice the intracellular.

B Estimation of Ptc-positive puncta size in germaria of the following genotypes: *Dronc*^{KO}/+ (n = 10). *ptc-Gal4* (BL2017)/+ (n = 9). *ptc-Gal4* (BL2017)/+; *Dronc*^{KO} Tub-*G80*^{ts} (BL7019)/+ (n = 10). The statistical significance between groups was established using one-way ANOVA Tukey's multiple comparisons test (****$P \leq 0.0001$, ***$P \leq 0.001$, *$P \leq 0.05$). Notice the enlargement of Ptc-positive puncta in a double heterozygous germaria (*ptc-Gal4*/+; *Dronc*+/−). The mean and the standard deviation are indicated in the graph.

C, D Expression of the *ptc*^{1130X}YFP (green) in representative germaria of the following genotypes: *ptc-Gal4* (BL2017)/UAS-*ptc*^{1130X}YFP (BL52215); Tub-*G80*^{ts} (BL7019) (C) *ptc-Gal4* (BL2017)/UAS-*ptc*^{1130X}YFP (BL52215); *Dronc*^{KO} Tub-*G80*^{ts} (BL7019)/+ (D). Dapi (blue) and FasIII (red) stainings label the nuclei and follicular cells, respectively. Notice the preferential accumulation of GFP signal within the ECs next to the boundary of FasIII expression in (D).

E Quantification of *ptc*^{1130X}-YFP expression levels in germaria of the following genotypes: CTRL = *ptc-Gal4* (BL2017)/ UAS-*ptc*^{1130X} YFP (BL52215); Tub-*G80*^{ts} (BL7019) (n = 8). *Dronc*+/− = *ptc-Gal4* (BL2017)/UAS-*ptc*^{1130X}YFP (BL52215); *Dronc*^{KO} Tub-*G80*^{ts} (BL7019)/+ (n = 16). An unpaired parametric Welch's t-test was used to determine statistical significance (****$P \leq 0.0001$). Median and quartiles are shown in the violin plot.

F Representative confocal image showing the expression of Ci-155 (blue and/or grey), *ptc*-GFP (green and/or grey) and Castor (red) in germaria of the following genotype *ptc-Gal4* (BL2017)/*ptc*-GFP^{CB02030}; *Dronc*^{KO} Tub-*G80*^{ts} (BL7019)/+. Notice that the expression levels of Ci, *ptc*-GFP and Castor are largely restored.

Data information: Scale bars represent 10 μm in all of the confocal images of the figure. All the experimental data shown have been obtained from $N \geq 2$ biological replicates. Experimental flies were kept after eclosion from the pupae for 14 days at 29°C prior to dissection. All the quantifications were made in germaria containing one single group of germline cells wrapped by follicular cells.
Source data are available online for this figure.

 © 2023 The Authors
