# Supplementary figures and images for: Non‐apoptotic caspase activation ensures the homeostasis of ovarian somatic stem cells
Source: EMBO Rep. 2023 Apr 11;24(6):e51716. doi: 10.15252/embr.202051716 (PMC10240206; doi:10.15252/embr.202051716)

**
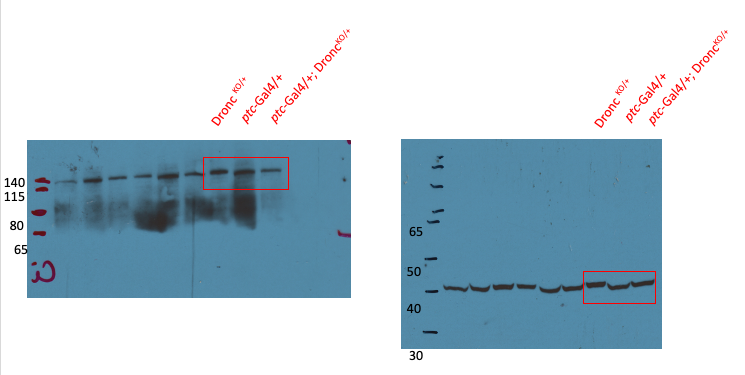
**

**Anti-Ci-155 Anti-actin**

**EV Figure 4K.** Uncropped images of the Western blots shown

Supplement: Supplementary file 3 — Source Data for Expanded View and Appendix [file EMBR-24-e51716-s005.zip › Source_data_EV_Figures_and_Appendix/Figure EV4/EV Figure 4K .docx]

**
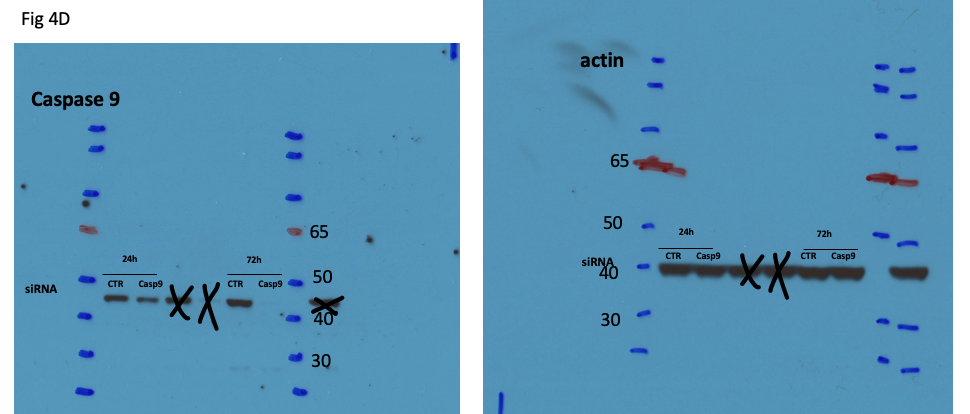
**

**Anti-Caspase-9 Anti-actin**

**Figure 4D.** Uncropped images of the Western blots

Supplement: Supplementary file 8 — Source Data for Figure 4 [file EMBR-24-e51716-s008.zip › Figure 4D.docx]

**
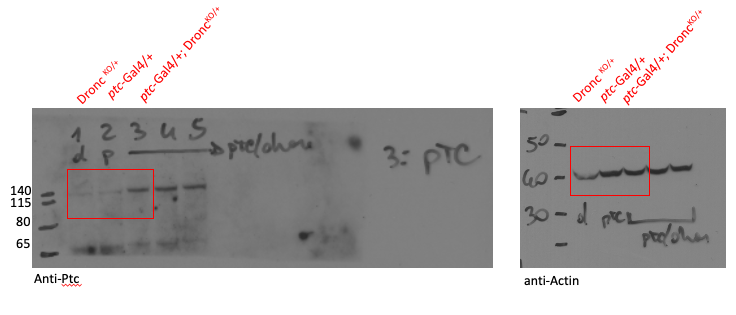
**

**Anti-Ptc Anti-actin**

**Figure 5C.** Uncropped images of the Western blots shown.

Supplement: Supplementary file 9 — Source Data for Figure 5 [file EMBR-24-e51716-s009.zip › Figure 5C.docx]

**
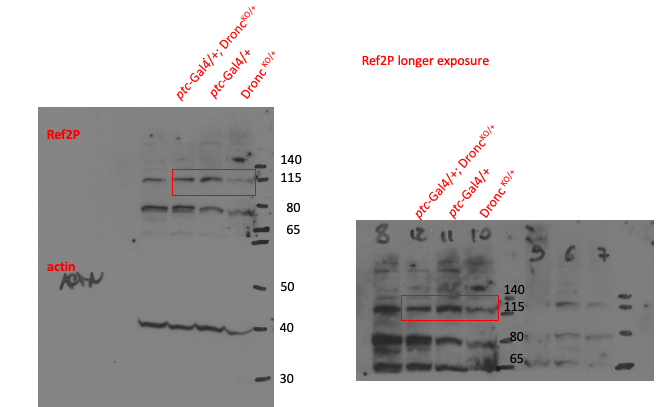
**

**Figure 6G.** Uncropped images of the Western blots shown.

Supplement: Supplementary file 10 — Source Data for Figure 6 [file EMBR-24-e51716-s003.zip › Figure 6G.docx]

**
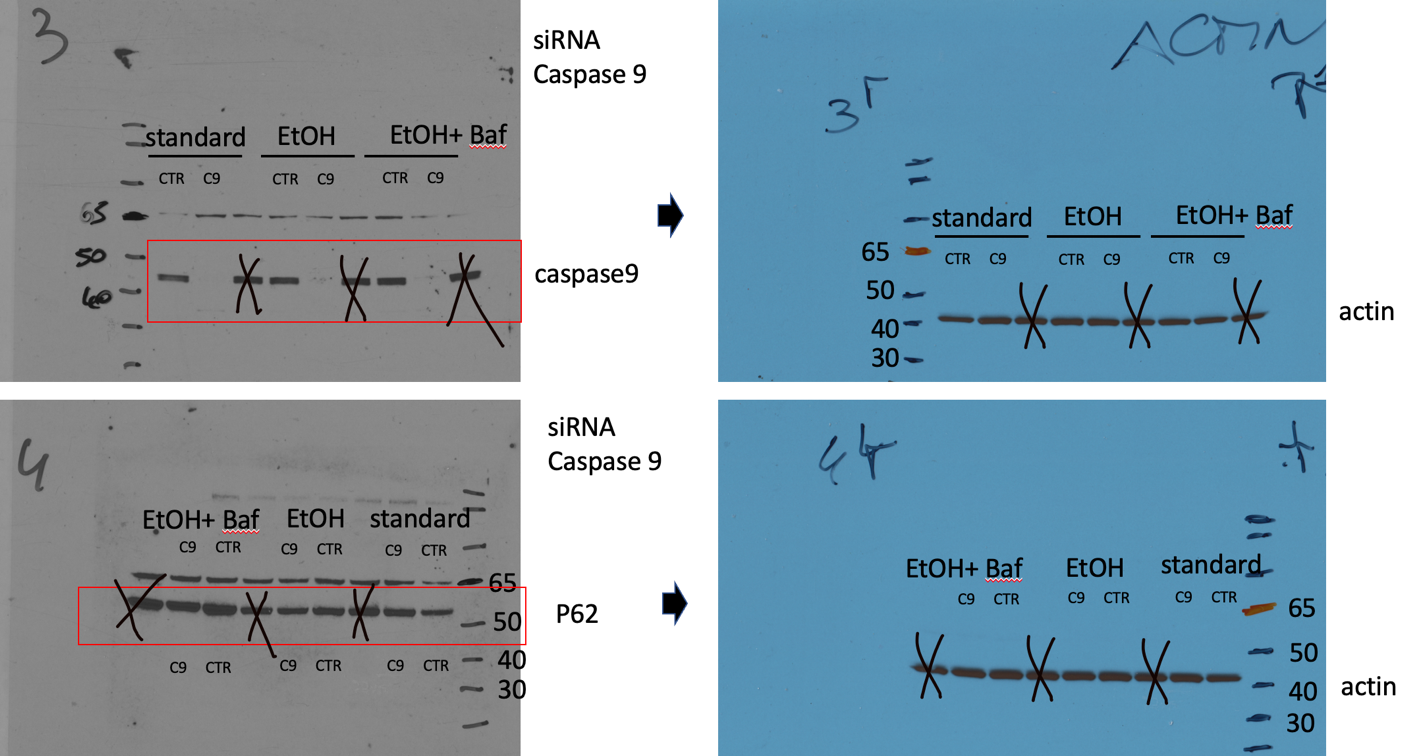
**

**Anti-P62 Anti-actin**

**Figure 6H.** Uncropped images of the Western blots shown.

Supplement: Supplementary file 10 — Source Data for Figure 6 [file EMBR-24-e51716-s003.zip › Figure 6H V2.docx]
