# Supplementary material for: Non‐apoptotic caspase activation ensures the homeostasis of ovarian somatic stem cells
Source: EMBO Rep. 2023 Apr 11;24(6):e51716. doi: 10.15252/embr.202051716 (PMC10240206; doi:10.15252/embr.202051716)
Supplement: Supplementary file 4 — PDF+ [file EMBR-24-e51716-s002.pdf]

Article

SOURCE DATA TRANSPARENT PROCESS OPEN ACCESS

EMBO reports

Non-apoptotic caspase activation ensures the homeostasis of ovarian somatic stem cells

Alessia Galasso[1], Derek Cui Xu[2], Claire Hill[3], Daria Iakovleva[4], Maria Irina Stefana[5] & Luis Alberto Baena-Lopez[2,*]

Abstract

Current evidence has associated caspase activation with the regulation of basic cellular functions without causing apoptosis. Malfunction of non-apoptotic caspase activities may contribute to specific neurological disorders, metabolic diseases, autoimmune conditions and cancers. However, our understanding of non-apoptotic caspase functions remains limited. Here, we show that non-apoptotic caspase activation prevents the intracellular accumulation of the Patched receptor in autophagosomes and the subsequent Patched-dependent induction of autophagy in *Drosophila* follicular stem cells. These events ultimately sustain Hedgehog signalling and the physiological properties of ovarian somatic stem cells and their progeny under moderate thermal stress. Importantly, our key findings are partially conserved in ovarian somatic cells of human origin. These observations attribute to caspases a pro-survival role under certain cellular conditions.

Keywords autophagy; caspases; Hedgehog signalling; non-apoptotic; ovarian stem cells

Subject Categories Autophagy & Cell Death; Development; Stem Cells & Regenerative Medicine

DOI 10.15252/embr.202051716 | Received 11 September 2020 | Revised 22 February 2023 | Accepted 14 March 2023 | Published online 11 April 2023

EMBO Reports (2023) 24: e51716

Introduction

Recent findings have associated the evolutionarily conserved family of enzymes known as caspases with the regulation of essential cellular functions distinct from apoptosis (Aram *et al*, 2017; Bell & Megeney, 2017; Baena-Lopez, 2018). These novel non-apoptotic caspase roles ensure tissue homeostasis while preventing the initiation and development of numerous diseases (Aram *et al*, 2017; Baena-Lopez, 2018). However, our understanding of non-apoptotic caspase functions is still very limited. During the last decade, the *Drosophila* ovary has played a key role in enhancing our understanding regarding the fundamental principles that regulate stem cell physiology and intercellular communication (Losick *et al*, 2011; Hayashi *et al*, 2020). Intriguingly, the somatic progenitor cells in the *Drosophila* ovary and their progeny can activate effector caspases at sublethal levels in response to environmental stress (Tang *et al*, 2015). Therefore, it is an ideal cellular system to study the interplay between caspases, signalling mechanisms and stem cell physiology.

The early development of *Drosophila* female gametes occurs in the germarium, a tissue composed of germline cells and supporting somatic cells (Losick *et al*, 2011; Fadiga & Nystul, 2019; Hayashi *et al*, 2020; Fig 1A). In the anterior regions of the germarium (regions 1 and 2a), the germline is supported by the escort cells (ECs; hereafter, escort cellular domain ECD; Fig 1A; Fadiga & Nystul, 2019; Hayashi *et al*, 2020). At the boundary between the regions 2a and 2b, a population of follicular stem cells (FSCs) gives rise to all the posteriorly located follicular cells (pre-FCs and FCs) and a subset of proximal anterior ECs (Fig 1A; Nystul & Spradling, 2010; Fadiga & Nystul, 2019). In regions 2b and 3 (hereafter, follicular cellular domain, FCD; Fig 1A), the germline is encapsulated to form independent follicles (Reilein *et al*, 2017; Fadiga & Nystul, 2019). Cell identity markers of the FCD are Fasciclin III (FasIII), Castor (Cas) and Eyes absent (Eya; Fig 1A; Nystul & Spradling, 2010; Chang *et al*, 2013). The cellular properties of the germarium are strongly influenced by the Hedgehog signalling pathway (Zhang & Kalderon, 2001; Vied & Kalderon, 2009; Rojas-Rios *et al*, 2012; Chang *et al*, 2013; Sahai-Hernandez & Nystul, 2013; Huang & Kalderon, 2014; Hayashi *et al*, 2020). The interaction of the Hedgehog (Hh) ligand with its membrane receptor Patched (Ptc) elicits the activation of the signalling transducer Smoothened (Smo; Briscoe & Therond, 2013). Smo subsequently prevents the proteolytic processing of the transcriptional regulator Cubitus interruptus (Ci; Briscoe & Therond, 2013; Hsia *et al*, 2015), thus controlling the expression of Hh target genes (Briscoe & Therond, 2013). The ECs and FSCs are the main somatic cells receiving Hh in the germarium (Rojas-Rios *et al*, 2012; Sahai-Hernandez & Nystul, 2013; Hayashi *et al*, 2020). The proliferation and differentiation of ovarian somatic precursors strongly rely on the activation of Hh pathway (Zhang & Kalderon, 2001; Chang *et al*, 2013; Sahai-Hernandez &

1 Faculty of Medicine Centre, Imperial College London, South Kensington Campus, London, UK
2 Sir William Dunn School of Pathology, University of Oxford, Oxford, UK
3 School of Medicine, Dentistry and Biomedical Sciences, Queen's University Belfast Medicine, Belfast, UK
4 Center for Regenerative Medicine, University of Edinburgh, Edinburgh, UK
5 Wellcome Center for Human Genetics, University of Oxford, Oxford, UK
*Corresponding author. Tel: +44 01865 618653; E-mail: alberto.baenalopez@path.ox.ac.uk

© 2023 The Authors. Published under the terms of the CC BY 4.0 license.

Nystul, 2013; Huang & Kalderon, 2014; Dai *et al*, 2017; Singh *et al*, 2018). Beyond the developmental requirements, Hh-signalling prevents the excess of autophagy induced by Ptc under stress conditions, thus ensuring the homeostasis of ovarian somatic cells (Hartman *et al*, 2013; Singh *et al*, 2018). Importantly, numerous Hh pathway functions in ovarian somatic cells are conserved in humans (Ray *et al*, 2011; Szkandera *et al*, 2013; Rosales-Nieves & Gonzalez-Reyes, 2014; Zeng *et al*, 2017).

Here, we investigate the potential caspase-dependent regulation of cellular properties in ovarian somatic cells. Our experiments suggest that the homeostasis of somatic cells in the ovary under moderate thermal stress requires transient bursts of non-apoptotic caspase activation. At the molecular level, non-apoptotic caspase activation appears to prevent the accumulation of Ptc in autophagosomes and the subsequent Ptc-dependent induction of autophagy. This ultimately sustains the level of Hh-signalling and the physiological properties of ovarian somatic cells. These observations attribute to caspases a previously unrecognised pro-survival role.

Results

Non-apoptotic patterns of *Dronc* activation in ovarian somatic stem cells

We have previously generated DBS-S-QF (Drice-based-sensor short QF), a genetic sensor based on a cleavable but catalytically inactive form of the effector caspase Drice (Baena-Lopez *et al*, 2018). Amongst other applications, this reporter provides a temporal perspective of initiator caspase activation by combining several cellular markers with variable cellular perdurance (Baena-Lopez *et al*, 2018; Fig EV1A). Whereas the sensor-dependent induction of tomato-HA expression and GFP report on either the ongoing or recent past caspase activation, the presence of β-gal labelling without any of the other signals are the unambiguous demonstration of past caspase activation without apoptosis (Baena-Lopez *et al*, 2018; Fig EV1A). Intrigued by the previously described non-apoptotic activation patterns of effector caspases in response to thermal (cold shock) or metabolic stress (starvation) in the *Drosophila* ovary (Tang *et al*, 2015), we used DBS-S-QF to investigate whether such activation was linked to initiator caspases. Interestingly, experiments conducted with our sensor under moderate thermal stress (29°C) consistently labelled putative somatic cells in the germarium (Fig 1B, full description of genotypes for all of the main figures can be found in Appendix Table S1). Furthermore, some DBS-S-QF positive cells did not show the fluorescent marker linked to ongoing caspase activation (tomato-HA), yet they expressed GFP (recent past caspase activation) without showing signs of apoptosis (TUNEL labelling; Fig 1B). These observations suggested the presence of transient and non-apoptotic bursts of caspase activation in the germarium. Supporting this hypothesis, we also noticed the presence of β-gal immunoreactivity in large populations of seemingly proliferative and therefore healthy ECs and FCs (Fig 1C; hereafter, raw data used for the quantitative analyses in the main Figures can be found in the Source data files). Furthermore, the proportion of germaria permanently labelled with β-gal significantly increased over time in flies kept at 29°C (Fig 1D). Co-immunostaining with FasIII and β-gal also revealed that the permanent labelling increase was not linked to germaria only showing β-gal in the ECD (Fig 1E) but to germaria with combined labelling in the ECD and FCD (Figs 1F and EV1B, full description of genotypes for all of the EV figures can be found in Appendix Table S2). These results specifically correlated the increased DBS-S-QF labelling with the proliferative activity of FSCs.

Since our sensor was designed to detect the activation of initiator caspases, we next evaluated the dependency of the DBS-S-QF labelling with Dronc; main initiator *Drosophila* caspase linked to non-apoptotic functions and orthologue of Caspase-2/9. The overexpression of a *Dronc*-RNAi transgene, under the control of *ptc-Gal4*, in all of the escort and follicular stem cells of the germarium reduced to residual levels the labelling with DBS-S-QF sensor (Fig 1G). Using a fly line expressing Gal4 under the physiological regulation of *Dronc* ($Dronc^{KO\text{-}Gal4}$; Arthurton *et al*, 2019), we correlated our previous findings with a consistent transcriptional activation of *Dronc* in both follicular stem cells and their progeny at 29°C (ECs and FCs; Figs 1H and I, and EV1C). Also, the so-called polar and stalk cells in more mature follicle chambers showed robust transcriptional activation of *Dronc* (Figs 1I and EV1C). Furthermore, taking advantage of a biotin ligase TurboID fused to Dronc (Shinoda *et al*, 2019), we detected an enrichment of Dronc in follicular (Fig 1J), polar and stalk cells (Fig EV1D). Collectively, these results strongly suggested that the ovarian follicular stem cells and their progeny (escort and follicular cells) can transiently activate Dronc in a non-apoptotic manner under moderate thermal stress (29°C).

Dronc activation sustains cell proliferation and differentiation in follicular cells

To determine the biological significance of non-apoptotic Dronc activation in the germarium, we generated morphogenetic mosaics using the $Dronc^{I29}$ null allele. $Dronc^{I29}$ homozygous cells showed a downregulation of the follicular marker Castor (Chang *et al*, 2013; Fig EV1E and F). However, the genetic mosaics showing this phenotype normally encompassed both somatic and germline cells (Fig EV1F). This situation precluded us to extract unambiguous conclusions about the origin of the phenotype. More importantly, these large mosaics were recovered with very low frequency after applying several heat shocks (Laws & Drummond-Barbosa, 2015; 11.4% (4/35); total number of analysed clones $n = 35$ in 85 germaria). Unfortunately, this experimental regime was largely incompatible with our aim to investigate the role of *Dronc* under moderate stress since it was previously shown to cause cell death in *Drosophila* tissues (Perez-Garijo *et al*, 2004). To circumvent these technical limitations, we capitalised on several *Dronc* conditional alleles generated in the laboratory through genome engineering (Arthurton *et al*, 2019; Fig EV2A). These alleles enable the efficient conversion of heterozygous wild-type cells containing an *FRT-Dronc-FRT* rescue cassette into homozygous mutant upon exposure to the Flippase recombinase (Fig EV2B; Arthurton *et al*, 2019). The *FRT-Dronc-FRT* rescue cassette was followed by the QF transcriptional activator in one of these alleles, thus allowing the expression of any cDNA of interest under the physiological regulation of *Dronc* upon cassette excision (Fig EV2B). Combining this allele with the *109-30*-Gal4 follicular driver (Fig EV2C and D; Sahai-Hernandez & Nystul, 2013), we efficiently eliminated Dronc expression (100% of inspected germaria, $n = 14$) in the prospective FCD and a subset of

 © 2023 The Authors

ECs (Fig EV2E). Interestingly, these genetic manipulations caused a characteristic set of morphological and genetic defects in the germarium (compare Fig 2A and B; compare Fig EV2E–H). Specifically, the total number of follicular cells and the total number of Castor-positive cells in the FCD were significantly reduced (Fig 2C). Furthermore, Castor expression was downregulated since the proportion of Castor-positive cells versus the total of FCs also diminished (Fig 2D). Equivalent results were obtained by eliminating

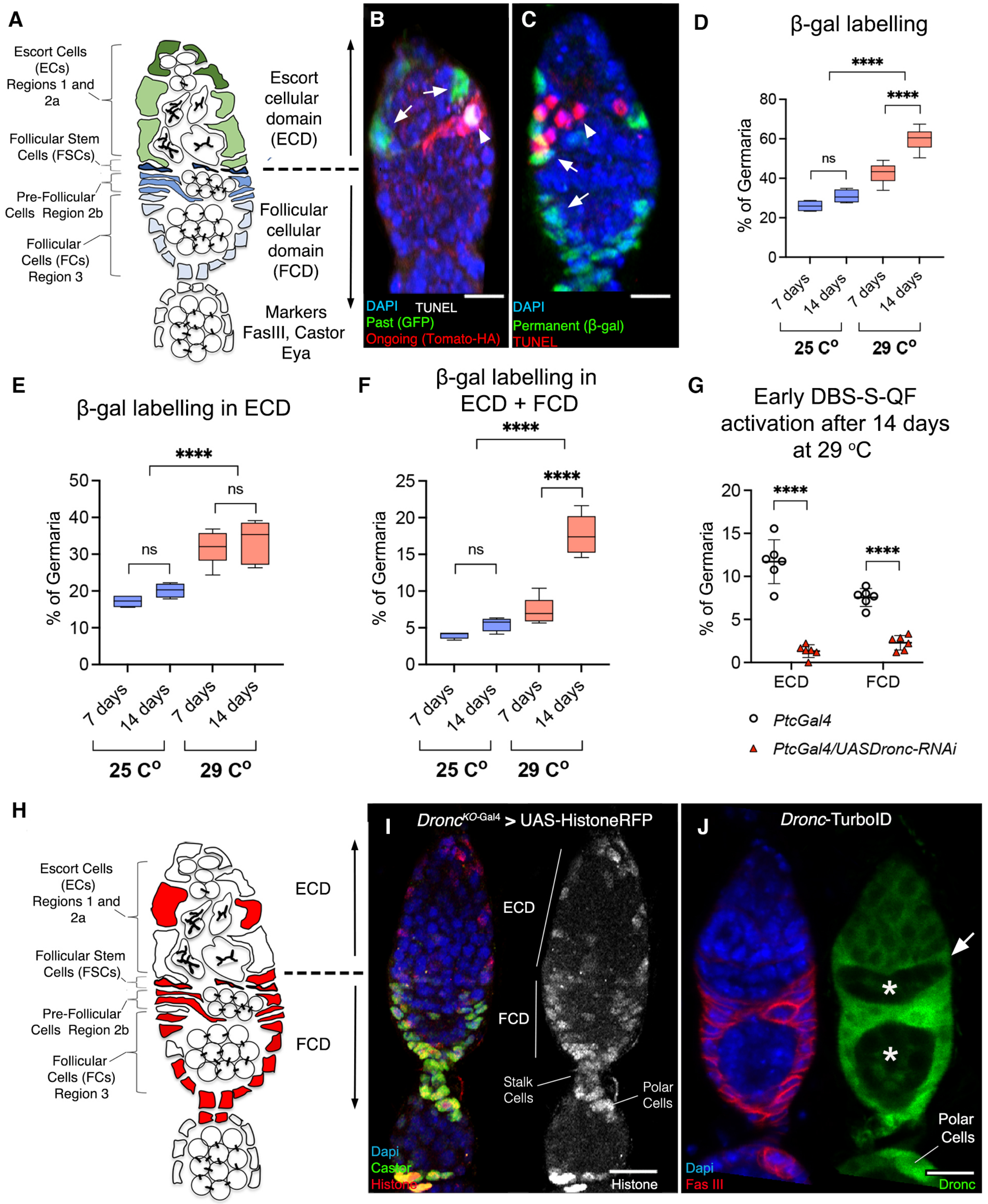

Figure 1.

Figure 1. Non-apoptotic activation of initiator caspases in somatic cells of the *Drosophila* germarium.

A Schematic drawing of the *Drosophila* germarium. Somatic cells relevant to this study (escort, follicular stem and pre-follicular) are, respectively, depicted in green, dark blue and light blue; germline cells are shown in white.

B Representative 2D projection of confocal images showing caspase activation at the dissection time point (red channel) and in the recent past (green channel, arrows) within putative escort somatic cells via DBS-S-QF sensor; TUNEL staining indicates apoptosis (grey, arrowhead); DAPI labels the nuclei in the entire Figure. Experimental flies were kept after eclosion from the pupae for 14 days at 29°C prior to dissection. Genotype: Actin *DBS-S-QF*, UAS-*mCD8-GFP*, QUAS-*tomato-HA*/+;;QUAS-*Gal4*/+ (BL83123).

C Representative 2D projection of confocal images showing escort and follicular somatic cells permanently labelled with DBS-S-QF sensor (green channel, arrows); the arrowhead indicates the presence of germline cells positive for TUNEL staining (red, arrowhead). Notice the lack of TUNEL signal and therefore apoptosis in somatic cells permanently labelled with DBS-S-QF sensor (green). Experimental flies were kept after eclosion from the pupae for 14 days at 29°C prior to dissection. Genotype: Actin *DBS-S-QF*, UAS-*mCD8-GFP*, QUAS-*tomato-HA*/+; QUAS-*flippase* (BL30126)/+; Actin5C FRT-stop-FRT *lacZ*-nls/+ (BL6355).

D Graph showing the percentage of germaria permanently labelled with DBS-S-QF overtime at 25°C (N = 4 biological replicates; n = 520 and n = 536 germaria were inspected at 7 and 14 days post adult eclosion, respectively) and 29°C (N = 7, n = 799 at 7 days and n = 588 at 14 days). A two-way ANOVA and Tukey's multiple comparison tests were used to determine statistical significance (n.s. = not significant; ****P < 0.0001). Genotype: Actin *DBS-S-QF*, UAS-*mCD8-GFP*, QUAS-*tomato-HA*/+; QUAS-*flippase* (BL30126)/+; Actin5C FRT-*stop*-FRT *lacZ*-nls/+ (BL6355).

E Percentage of germaria showing permanently labelled with DBS-S-QF the ECD over time at 25 and 29°C. FasIII immunostaining was used to locate the DBS-S-QF labelling in the inspected germaria. The N and n numbers of these experiments are indicated in (D). A two-way ANOVA and Tukey's multiple comparison tests were used to determine statistical significance (n.s., not significant; ****P < 0.0001). Genotypes are shown in (D).

F Percentage of germaria showing permanently labelled with DBS-S-QF the ECD over time at 25 and 29°C. FasIII immunostaining was used to locate the DBS-S-QF labelling in the inspected germaria. The N and n numbers of these experiments are indicated in (D). A two-way ANOVA and Tukey's multiple comparison tests were used to determine statistical significance (n.s., not significant; ****P < 0.0001). Genotypes are shown in (D).

G Graph comparing the caspase activation levels reported by DBS-S-QF at the dissection time point (tomato-HA) in different regions of the germarium with and without *Dronc* expression. Experimental flies were kept after eclosion from the pupae for 14 days at 29°C prior to dissection. FasIII immunostaining was used to locate the DBS-S-QF labelling in the different regions of the germarium. N = 6 biological replicates; *ptc-Gal4* (n = 294) and *ptc-Gal4* UAS-*Dronc*-RNAi (n = 404) inspected germaria. An unpaired parametric Welch's t-test was used to determine statistical significance (****P < 0.0001). The graph shows the mean and standard deviations. Genotypes: Actin *DBS-S-QF*, UAS-*mCD8-GFP*, QUAS-*tomato-HA*/+; *ptc-Gal4* (BL2017)/+ and Actin *DBS-S-QF*, UAS-*mCD8-GFP*, QUAS-*tomato-HA*/+; *ptc-Gal4* (BL2017)/UAS-Dronc-RNAi (a gift from Pascal Meier).

H Schematic drawing of the *Drosophila* germarium illustrating the somatic cells relevant consistently showing Dronc transcription (red).

I Representative 2D projection of confocal images showing the activation of UAS-Histone-RFP (red channel, arrows) under the regulation of *Dronc*$^{KO\text{-}Gal4}$ after 7 days at 29°C; the follicular marker Castor is shown in green. Genotype: w;; *Dronc*$^{KO\text{-}Gal4}$/UAS-*Histone-RFP* (BL56555).

J Biotinylation signal (green) generated in the germarium by a *Dronc*-TurboID allele; notice the signal enrichment in the presumptive follicular stem cells (white arrows) and follicular cells; notice that germline cells express relative low levels of Dronc (asterisk). FasIII immunostaining (red) labels the somatic follicular cells. DAPI labels the nuclei (blue). Experimental flies were kept after eclosion from the pupae for 7 days at 29°C prior to dissection. Genotype: w;; *Dronc*^{TurboID} (a gift from Masayuki Miura)/Tm3, Sb.

Data information: Scale bars represent 10 μm in the entire Figure. Full description of genotypes for all of the main figures can be found in Appendix Table S1. The box plots show the median, first quartile and third quartile of datasets. The whiskers illustrate the range between the maximum and minimum values of datasets.
Source data are available online for this figure.

Dronc expression in all of the ECs and FSCs with *ptc-Gal4* (Hartman *et al*, 2015; Figs 2C and D, and EV2I–K).

To better determine the origin of the reduction in the number of follicular cells, we analysed the rate of apoptosis and the cell cycle profile in our mutant conditions. Predictably, low levels of Dronc in somatic cells reduced the rate of apoptosis in the germarium while its overexpression induced cell death (reduction in TUNEL staining, Fig 2E). These genetic manipulations also caused nonautonomous apoptosis in the germline (notice the TUNEL staining in groups of cells negative for somatic markers such as FasIII and Histone-RFP; arrows; Fig 2F and G) that could indicate cell communication defects between somatic cells and the germline (Sahai-Hernandez *et al*, 2012; Shi *et al*, 2021). These results dissociated the growth and differentiation phenotypes induced by caspase deficiency from the process of apoptosis itself since one would expect an excess of follicular cells upon inhibiting apoptosis, as opposed to a reduction.

Next, we analysed the impact of Dronc deficiency on cell proliferation by using several cell cycle markers. The Fly-Fucci construct reports on the cell cycle progression by labelling the different stages with distinguishable fluorescent markers (Zielke *et al*, 2014; Fig 2H). This tool revealed that follicular cells with limited Dronc expression were accumulated in the S-phase at the expense of other cell cycle stages (Fig 2I–K). The accumulation of cells in S-phase was also correlated with a significant increase in EdU labelling (S-phase marker; Fig EV2L, hereafter, raw data used for the quantitative analyses in Figs EV can be found in the Source data files). Since the excess of cells in the S-phase in these experiments did not increase the total number of FCs but caused a reduction (Fig 2C), we concluded that Dronc deficiency slows down the transition through the S-phase of FCs.

To discard potential unwanted effects of QF expression and validate the association of the phenotypes with Dronc, we analysed the behaviour of a different conditional Dronc allele in the follicular cells; *Dronc*$^{FRT\text{-}Dronc\text{-}FRT\text{-}suntag\text{-}HA\text{-}cherry}$ (Arthurton *et al*, 2019). This allele expresses a synthetic suntag-HA-mCherry peptide instead of QF upon stop cassette excision (Fig EV2A). Follicular cells only expressing Dronc$^{suntag\text{-}HA\text{-}Cherry}$ showed proliferation and differentiation defects comparable to that of cells without Dronc protein (Fig 3A–C). Collectively, our data suggested that Dronc activation facilitates the proliferation and differentiation in the FCD at 29°C.

The entire apoptotic pathway can be engaged for non-lethal purposes in ovarian somatic cells

To assess the molecular features of Dronc required in our experimental scenario, we used an allele that can conditionally express a full-length but enzymatically inactive form of Dronc (*Dronc*$^{FRT\text{-}Dronc\text{-}FRT\text{-}FLCAEA}$; Fig EV2A; Arthurton *et al*, 2019).

 © 2023 The Authors

Dronc^{FLCAEA} contains two mutations that prevent its enzymatic activity (C318A) and proteolytic activation (E352A; Chai *et al*, 2003; Muro *et al*, 2004). Follicular cells exclusively expressing Dronc^{FLCAEA} showed proliferation and differentiation defects (Fig 3A–C) that suggested an enzymatic requirement of Dronc in our experimental scenario. Next, we evaluated whether the implementation of Dronc functions could be mediated by the effector caspases; main Dronc substrates during apoptosis. To that end, we co-overexpressed in the FSCs validated RNAi constructs able to reduce their expression (Drice, DCP-1, Decay and Damm; Leulier *et al*, 2006; Fig EV3A). This genetic manipulation caused proliferation and differentiation phenotypes reminiscent of Dronc deficiency (Fig 3D–F). Since effector caspases can be functionally redundant (Xu *et al*, 2006), we separately assessed the contribution of DCP-1 and Drice to these phenotypes. Although these experiments also caused proliferation defects, the differentiation phenotypes were weaker (Fig 3D–F; notice the proportion of Castor-expressing cells versus the total number of follicular cells was reduced to a lesser extent), thus suggesting a likely implementation of Dronc functions mediated by the combinatorial activity of effector caspases. Supporting this hypothesis, the overexpression of either Diap-1 or P35 (a viral pan-effector caspase inhibitor (Hay *et al*, 1994)) also limited the proliferation and differentiation of ovarian somatic cells (Fig EV3A–C). In these experiments, we used *ptc-Gal4* to consistently reduce the activity of effector caspases in the entire ECD and the FSCs. In a different set of experiments, we evaluated the potential mechanisms of Dronc activation by ectopically expressing either a short-hairpin RNA against Dark (*Drosophila* orthologue of Apaf-1; Obata *et al*, 2014) or a microRNA against the main pro-apoptotic factors (Hid, Reaper and Grim; RHG; Siegrist *et al*, 2010), respectively (Fig EV3A). Intriguingly, these experiments reduced the total number of follicular and Castor-positive cells (Fig 3D and E); however, the proportion of Castor-expressing cells versus the total was not affected (Fig 3F). These findings indicated that differentiation and proliferation phenotypes can be uncoupled. More importantly, our data provided evidence that the entire caspase pathway can be activated for non-apoptotic purposes in somatic cells of the ovary.

Dronc activation can facilitate Hh-signalling

The Hh pathway is activated in follicular stem cells and their progeny; *ptc* is a universal transcriptional Hh target (Briscoe & Therond, 2013) and the Ci-155 antibody recognises the activated form of Ci (Motzny & Holmgren, 1995; Figs 4A and EV4A). This activation sustains the cell proliferation and differentiation in the FCD (Zhang & Kalderon, 2001; Chang *et al*, 2013; Sahai-Hernandez &

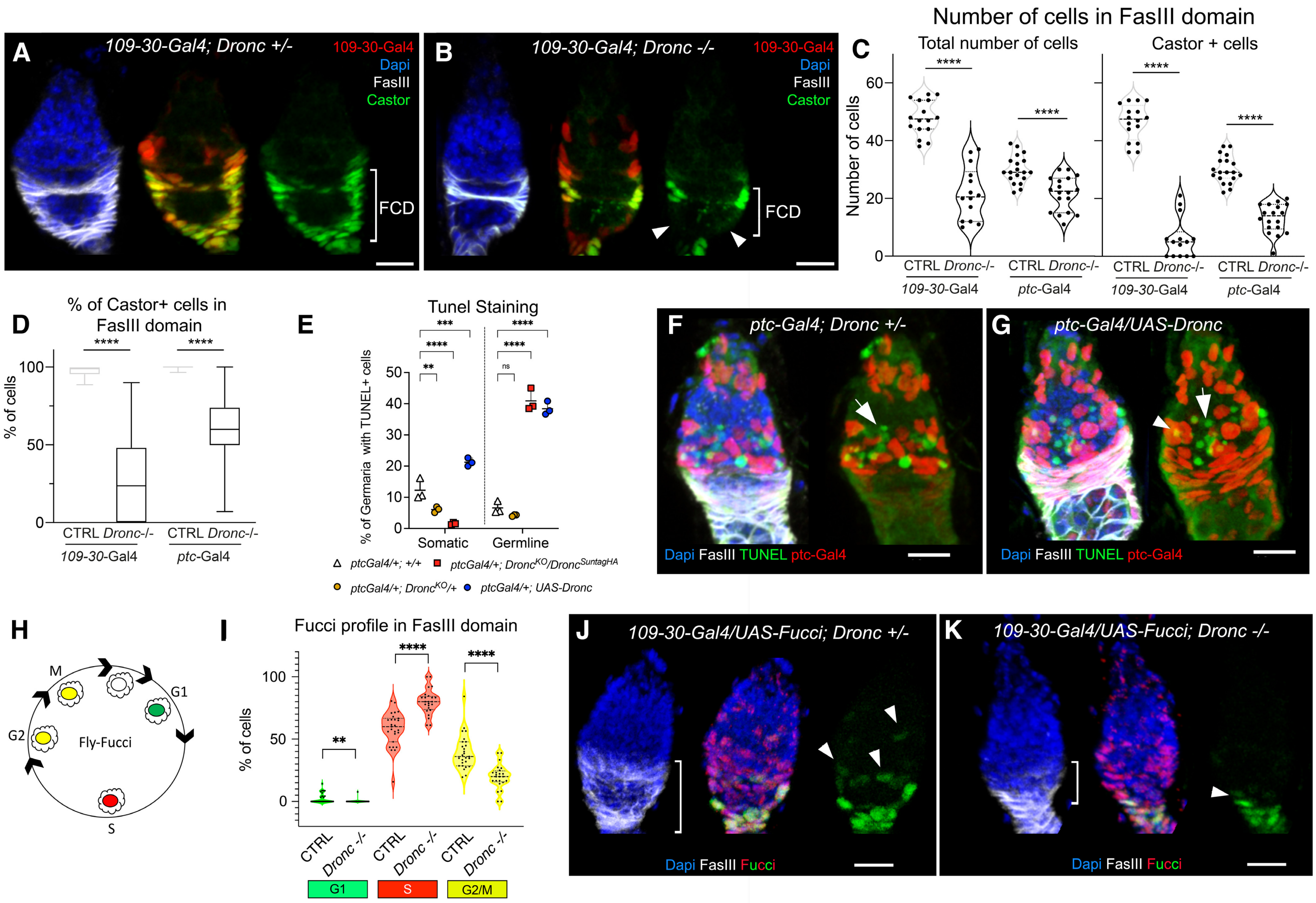

Figure 2.

◀ **Figure 2. Functional characterisation of *Dronc* in somatic cells.**

A, B Representative 2D projection of confocal images showing the expression of the cell identity Castor in follicular cells control with Dronc expression (A: *109-30-Gal4* (BL7023)/+; *Dronc*^{KO} UAS-*Histone-RFP* (BL56555) Tub-*G80*^{ts} (BL7019)/+) and without Dronc (B: *109-30-Gal4* (BL7023)/+; *Dronc*^{KO} UAS-*Histone-RFP* (BL56555) Tub-*G80*^{ts} (BL7019)/UAS-*flippase* (BL8209) *Dronc*$^{KO\text{-}FRT\text{-}Dronc\text{-}GFP\text{-}APEX\text{-}FRT\text{-}QF}$). Notice the reduction in the number of Castor-expressing cells in the FCD and the reduction in size of this region in (B). Nuclei are labelled with DAPI (blue); Castor (green) and FasIII (grey) label the follicular cells; UAS-Histone-RFP labels the nuclei of Gal4-expressing cells (red).

C Quantification of total number of follicular cells (left) and Castor-expressing cells (right) within the FCD (FasIII positive region) in either heterozygous or homozygous *Dronc* mutant conditions: Genotypes: *CTRL* = *109-30-Gal4* (BL7023)/+; *Dronc*^{KO} UAS-*Histone-RFP* (BL56555) Tub-*G80*^{ts} (BL7019)/+; *Dronc–/–* = *109-30-Gal4* (BL7023)/+; *Dronc*^{KO} UAS-*Histone-RFP* (BL56555) Tub-*G80*^{ts} (BL7019)/UAS-*flippase* (BL8209) *Dronc*$^{KO\text{-}FRT\text{-}Dronc\text{-}GFP\text{-}APEX\text{-}FRT\text{-}QF}$.*CTRL* = *ptc-Gal4* (BL2017)/+; UAS-*Histone-RFP* (BL56555) Tub-*G80*^{ts} (BL7019)/+. *Dronc–/–* = *ptc-Gal4* (BL2017)/+; *Dronc*^{KO} UAS-*Histone-RFP* (BL56555) Tub-*G80*^{ts} (BL7019)/ UAS-*flippase* (BL8209) *Dronc*$^{KO\text{-}FRT\text{-}Dronc\text{-}GFP\text{-}APEX\text{-}FRT\text{-}QF}$. $N = 2$; the n number for each column in order of appearance $n = 16$, $n = 14$, $n = 20$, $n = 17$, $n = 19$, $n = 18$. Statistical significance was determined by using an unpaired parametric Welch's *t*-test (****$P \le 0.001$). The median and quartiles are indicated in the violin plots.

D Percentage of Castor-expressing cells versus the total number of Follicular cells in germaria of the genotypes indicated in (C). The box plot shows the median, first quartile and third quartile of the dataset. The whiskers illustrate the range between the maximum and minimum values of the dataset. Statistical significance was established by using a nonparametric Mann–Whitney *t*-test (****$P \le 0.0001$). *n* numbers and genotypes are shown in (C).

E Percentage of germaria showing TUNEL staining in different cell types of the germarium (somatic cells and germline cells) upon altering *Dronc* expression in all of the escort cells and follicular stem cells. $N = 3$ biological replicates; the n number of inspected germaria per condition is indicated close to the genotype. The genotypes in the graph are as follows: White triangle ($n = 378$ germaria) = *ptc-Gal4* (BL2017)/+; +/+. Yellow circle ($n = 381$) = *ptc-Gal4* (BL2017)/+; *Dronc*^{KO} UAS-*Histone-RFP* (BL56555) Tub-*G80*^{ts} (BL7019)/+. Red square ($n = 196$) = *ptc-Gal4* (BL2017)/+; *Dronc*^{KO} UAS-*Histone-RFP* (BL56555) Tub-*G80*^{ts} (BL7019)/UAS-*flippase* (BL8209) *Dronc*$^{KO\text{-}FRT\text{-}Dronc\text{-}GFP\text{-}APEX\text{-}FRT\text{-}suntag\text{-}HA}$. Blue circle ($n = 184$) = *ptc-Gal4* (BL2017)/UAS-*Dronc* (BL56198); *Dronc*^{KO} UAS-*Histone-RFP* (BL56555) Tub-*G80*^{ts} (BL7019)/+. An ordinary one-way ANOVA and Holm–Sidak's multiple comparison test were used to determine statistical significance (n.s., not significant; **$P \le 0.01$; ***$P \le 0.001$; ****$P \le 0.0001$). The graph shows the mean and standard deviations.

F Representative 2D projection of confocal images showing TUNEL staining (green) in a germarium *ptc-Gal4* (BL2017)/+; *Dronc*^{KO} UAS-*Histone-RFP* (BL56555) Tub-*G80*^{ts} (BL7019)/+. UAS-*Histone-RFP* expression induced by *ptc-Gal4* is shown in red. Nuclei are labelled with DAPI (blue). FasIII (grey) labels the follicular cells. Notice a group of germline cells (negative for FasIII and Histone-RFP) positive for TUNEL (green; white arrow).

G Representative 2D projection of confocal images showing TUNEL staining (green) in a germarium *ptc-Gal4* (BL2017)/+; *Dronc*^{KO} UAS-*Histone-RFP* (BL56555) Tub-*G80*^{ts} (BL7019)/UAS-*Dronc* (BL56198). UAS-*Histone-RFP* expression induced by *ptc-Gal4* is shown in red. Nuclei are labelled with DAPI (blue). FasIII (grey) labels the follicular cells. Notice a group of germline cells (negative for FasIII and Histone-RFP) positive for TUNEL (green; white arrow). Overlap of TUNEL with Histone-RFP and FasIII indicates apoptosis of a somatic cell (arrowhead).

H Diagram illustrating the labelling of the different stages in the cell cycle using UAS-Fly-Fucci.

I Graph quantifying the Fly-FUCCI labelling in follicular cells with or without Dronc expression. Genotypes: CTRL ($N = 2$; $n = 27$) = *109-30-Gal4* (BL7023)/UAS-FUCCI (BL55100); *Dronc*^{KO} Tub-*G80*^{ts} (BL7019)/+ (*Dronc–/–*: ($N = 2$; $n = 24$) = *109-30-Gal4* (BL7023)/UAS-FUCCI(BL55100); *Dronc*^{KO} Tub-*G80*^{ts} (BL7019)/UAS-*flippase* (BL8209) *Dronc*$^{KO\text{-}FRT\text{-}Dronc\text{-}GFP\text{-}APEX\text{-}FRT\text{-}suntagHA}$). FasIII staining was used as a reference to locate the follicular cells in the inspected germaria. Notice the accumulation of cells in S-phase (red signal) at expense of other cell cycle stages. A nonparametric Mann–Whitney *t*-test was used to determine statistical significance (**$P \le 0.01$; ****$P \le 0.0001$). The median and quartiles are indicated in the violin plots.

J, K Representative 2D projection of confocal images of the genotypes quantified in I. Nuclei are labelled with DAPI (blue). Red and green nuclei show the expression of different Fucci markers. FasIII (grey) was used to locate the FCD. Notice the reduction of follicular cells and limited overlap between the green and red fluorescent signals (white arrowheads) in (K).

Data information: Scale bars represent 10 μm. Experimental flies were kept after eclosion from the pupae for 14 days at 29°C prior to dissection (applicable to all panels).
Source data are available online for this figure.

Nystul, 2013; Huang & Kalderon, 2014; Dai *et al*, 2017; Singh *et al*, 2018). Accordingly, our experiments either reducing the expression of Ci in FCs or overexpressing *ptc*^{1130X} in Hh-expressing cells compromised the proliferation and differentiation of FCs (Fig EV4B–E). *ptc*^{1130X} encodes a form of Ptc receptor that efficiently prevents the activation of the Hh pathway in Hh-producing cells (Johnson *et al*, 2000; Lu *et al*, 2006). Since both Hh-signalling and caspase activation are able to sustain the cellular properties of FCs, we decided to investigate in detail their potential interplay. As indicated by *ptc*-GFP expression and Ci^{155} immunoreactivity, the activation of the Hh pathway was downregulated in FCs without Dronc (Fig 4A–C). Importantly, comparable results were obtained upon reducing Caspase-9 in OVCAR-3 cells (ovarian somatic cells of human origin (Godwin *et al*, 1992); Fig 4D and E). To investigate how Dronc deficiency could be limiting Hh-signalling, we overexpressed either Ci or a constitutively active form of Smo in follicular cells without Dronc. Importantly, these genetic manipulations rescued the growth and Castor expression (compare Fig 2A–C with Figs 4F–I, and EV4F and G). We then evaluated whether the Hh-ligand was competent for signalling in FCs without Dronc. Since Hh is a diffusible extracellular ligand (Briscoe & Therond, 2013), we attempted to mainly restrict its overexpression within Hh-expressing cells by using the *ptc-Gal4* driver. As previously reported (Chang *et al*, 2013), the excess of Hh caused a dramatic expansion of Castor-positive cells and the fusion of egg chambers in otherwise wild-type germaria (Fig EV4H). However, these phenotypes were partially rescued upon limiting Dronc expression in ECs and FSCs; notice the smaller proportion of fused egg chambers with continuous Castor expression and moderate increase in the number of stalk cells (Fig EV4I and J). These results suggested a potential intersection of Dronc with the Hh pathway upstream of Smo.

Ptc is the receptor of Hh that represses the activation of Smo in an unbound state to Hh (Briscoe & Therond, 2013; Hsia *et al*, 2015). Strikingly, double heterozygous *ptc-Gal4:Dronc* germaria (*ptc-Gal4*/+; *Dronc*^{KO}/+) showed differentiation defects compatible with Hh deficiency instead of a Hh gain-of-function (Figs 4J and K, and EV4K, and Appendix Fig S2). This phenotype was also linked to lower levels of *ptc*-GFP and Ci-155 expression (Fig EV4M); notice that *ptc-Gal4* and *ptc*-GFP are both weak hypomorph transcriptional *ptc* alleles (Shyamala & Bhat, 2002; Buszczak *et al*, 2007). Furthermore, double heterozygous *ptc*^{S2}:*Dronc*^{KO} germaria (*ptc*^{S2}/+; *Dronc*^{KO}/+) also showed a reduction in the number of Castor-

 © 2023 The Authors

expressing cells (Fig EV4N and L); ptc^{S2} is a null allele. The specificity of the genetic interaction between Ptc and Dronc was corroborated by overexpressing Dronc in *ptc-Gal4:Dronc*^{KO} germaria, since this genetic manipulation was sufficient to rescue the Castor expression and therefore the differentiation defects (Fig 4J and K). These results provided genetic evidence about an intriguing intersection of Dronc activation with the Hh pathway at the level of Ptc.

Dronc activation prevents Ptc accumulation in autophagosomes

The localisation of Ptc receptor in the plasma membrane is essential to limit the activation of Smo in cells not receiving Hh (Briscoe & Therond, 2013) and to promote Hh degradation upon internalisation (Hsia *et al*, 2015). To better understand a potential interplay between Dronc and the Hh pathway at the level of Ptc, we decided to investigate the distribution of Ptc in *ptc-Gal4:Dronc* germaria. Immunostainings using anti-Ptc antibody showed a significant increase in Ptc immunoreactivity in *ptc-Gal4:Dronc* mutant germaria (Fig 5A and B). Furthermore, the cytoplasmic Ptc puncta (Fig EV5A) were significantly increased in size in the mutant condition (Fig EV5B). The excess of Ptc was also detected by western blot (Fig 5C). These results were unexpected since we previously showed a transcriptional downregulation of *ptc* in somatic cells without Dronc (Fig 4B). To assess whether Ptc could

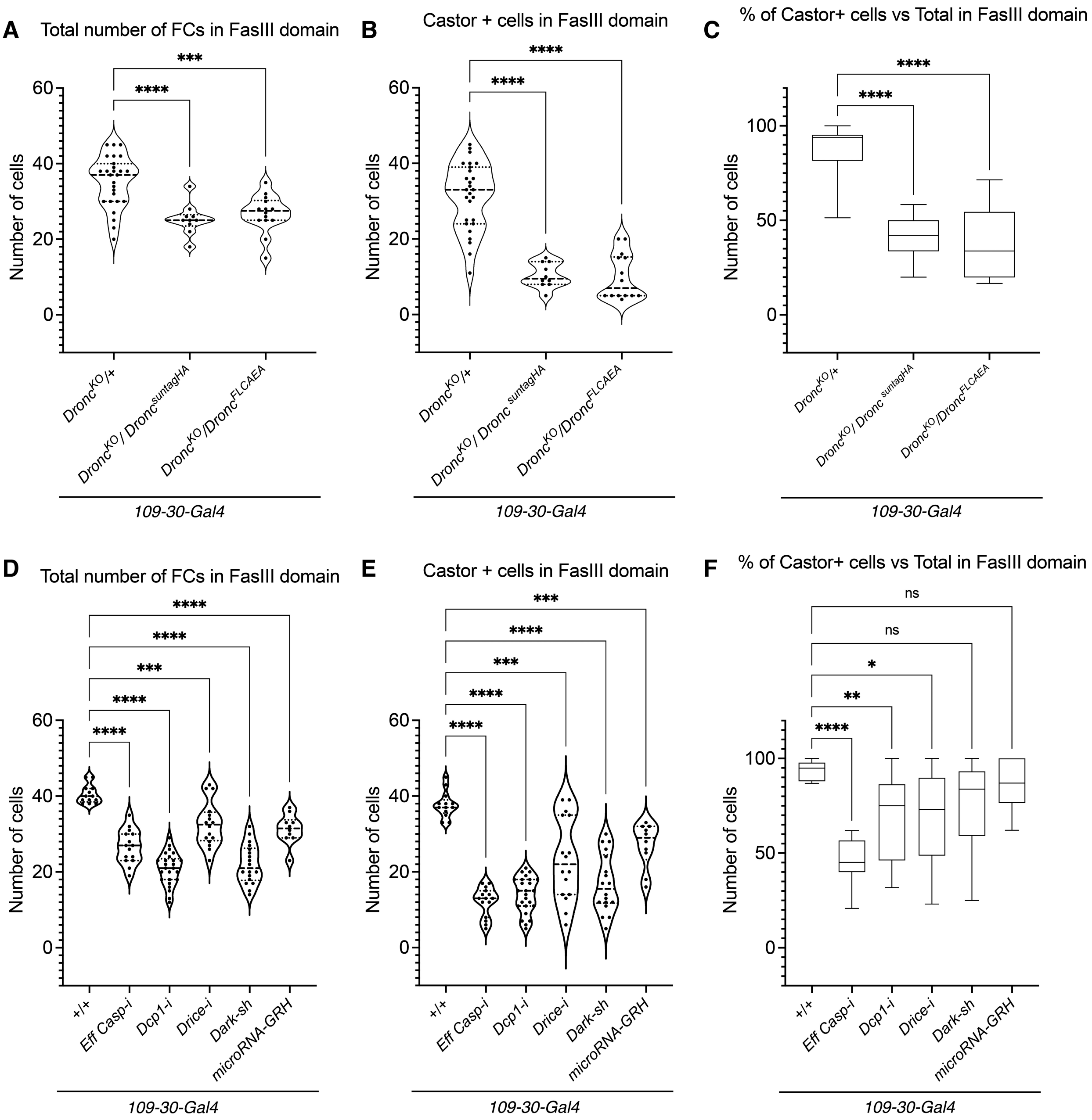

Figure 3.

Figure 3. Enzymatic activity of Dronc and the entire caspase pathway is required to sustain the cellular properties of ovarian somatic cells.

A Quantification of total number of follicular cells in germaria with follicular stem cells expressing normal levels of Dronc (*Dronc*^{KO}/+), without Dronc expression (*Dronc*^{KO}/*Dronc*$^{KO\text{-Suntag-HA-Cherry}}$), and expressing a catalytically inactive form of Dronc (*Dronc*^{KO}/*Dronc*$^{KO\text{-FL-CAEA-Suntag-HA-Cherry}}$). The genotypes are as follows: *109-30-Gal4* (BL7023)/+ (n = 13). *109-30-Gal4* (BL7023)/+; *Dronc*^{KO} Tub-*G80*^{ts} (BL7019)/UAS-*flippase* (BL8209) *Dronc*$^{KO\text{-FRT Dronc-GFP-Apex FRT-Suntag-HA-Cherry}}$ (n = 15). *109-30-Gal4* (BL7023)/+; *Dronc*^{KO} Tub-*G80*^{ts} (BL7019)/UAS-*flippase* (BL8209) *Dronc*$^{KO\text{-FRT Dronc-GFP-Apex FRT-Dronc FL-CAEA-Suntag-HA-Cherry}}$ (n = 14). A one-way ANOVA and Dunnett's multiple comparison post-test were used to determine statistical significance (***$P \le 0.001$; ****$P \le 0.0001$).

B Quantification of total Castor-expressing cells within the FasIII cellular domain in the genotypes shown in (A). n numbers are shown in (A). A one-way ANOVA and Dunnett's multiple comparison post-test were used to determine statistical significance (****$P \le 0.0001$).

C Percentage of Castor-expressing cells versus the total number of Follicular cells in germaria of the genotypes indicated in (A). A Kruskal–Wallis test and Dunn's multiple comparison post-test were used to determine statistical significance (****$P \le 0.0001$).

D Quantification of total number of follicular cells within the FasIII cellular domain in the following genotypes: *109-30-Gal4*/+; Tub-*G80*^{ts} (BL7019)/+ (n = 16). *109-30-Gal4* (BL7023)/UAS-*Drice*RNAi UAS-*Decay*RNAi (a gift from Pascal Meier); UAS-*Damm*RNAi, UAS-*Dcp1*RNAi (a gift from Pascal Meier) (n = 15). *109-30-Gal4* (BL7023)/+; Tub-*G80*^{ts} (BL7019)/UAS-*Dcp1*RNAi (BL28909) (n = 25) *109-30-Gal4* (BL7023)/+; Tub-*G80*^{ts} (BL7019)/UAS-*Drice*RNAi (BL32403) (n = 16) *109-30-Gal4* (BL7023)/UAS-*Dark-sh*; Tub-*G80*^{ts} (BL7019)/+ (a gift from M. Miura) (n = 18). *109-30-Gal4* (BL7023)/UAS-microRNA-RHG; Tub-*G80*^{ts} (BL7019)/+ (a gift from Iswar Hariharan) (n = 10). A one-way ANOVA and Dunnett's multiple comparison post-test were used to determine statistical significance (***$P \le 0.001$; ****$P \le 0.0001$).

E Quantification of total number of Castor-expressing cells in germaria of the genotypes indicated in (D). n numbers are shown in (D). A one-way ANOVA and Dunnett's multiple comparison post-test were used to determine statistical significance (***$P \le 0.001$; ****$P \le 0.0001$).

F Percentage of Castor-expressing cells versus the total number of Follicular cells (FasIII^{+} cells) in germaria of the genotypes indicated in (A). A Kruskal–Wallis test and Dunn's multiple comparison post-test were used to determine statistical significance (n.s., not significant, *$P \le 0.05$, **$P \le 0.01$, ****$P \le 0.0001$).

Data information: Experimental flies were kept after eclosion from the pupae for 14 days at 29°C prior to dissection (applicable to all panels). All the experimental data shown have been obtained from $N \ge 2$ biological replicates. The median and quartiles are indicated in the violin plots. The box plots show the median, first quartile and third quartile of datasets. The whiskers illustrate the range between the maximum and minimum values of datasets. All the quantifications were made in germaria containing one single group of germline cells wrapped by follicular cells.
Source data are available online for this figure.

be intracellularly accumulated either on its way to the plasma membrane or after Hh-mediated internalisation, we investigated the distribution of Ptc^{1130X}, a highly stable form of Ptc in the plasma membrane with low internalisation rate (Lu *et al*, 2006). Our experiments showed again a striking accumulation of Ptc^{1130X} in somatic cells without Dronc (Fig EV5C–E). These data strongly suggested that Dronc activation can post-translationally prevent the intracellular accumulation of Ptc likely before reaching the plasma membrane.

Intriguingly, recent reports have revealed a function of Ptc as a regulator of autophagy in the *Drosophila* ovary independent of Hh pathway (Jimenez-Sanchez *et al*, 2012; Singh *et al*, 2018). In mammalian cells, it has also been reported the autophagy-dependent degradation of Ptch1 (Yang *et al*, 2021). These findings prompted us to explore whether the Ptc aggregates observed in *ptc-Gal4:Dronc* germaria could be localised in autophagosomes. To this end, we performed colocalisation experiments between Ptc and the autophagy regulator Atg8 (Slobodkin & Elazar, 2013). These experiments showed a significantly increased colocalisation between Ptc and Atg8 in *ptc-Gal4:Dronc* germaria (Fig 5D and E), a higher proportion of Ptc in Atg8+ autophagosomes (Fig 5F), and more Atg8+ autophagosomes loaded with Ptc (Fig 5G). To assess the biological significance of Ptc accumulation in autophagosomes, we reduced Ptc protein levels without affecting its molecular features by overexpressing a Ptc RNAi construct in FSCs. These experiments rescued the proliferation and differentiation defects shown by FCs without Dronc (Fig 5H and I). A compatible phenotypic rescue was obtained by using a heteroallelic combination for *ptc* that reduced the transcription efficiency of the gene without affecting the molecular structure of the protein (Fig EV5F). Collectively, our experiments revealed a previously unrecognised ability of Dronc to prevent the accumulation of Ptc in autophagosomes. They also underscored the relevance of limiting the intracellular accumulation of Ptc to maintain the homeostasis of ovarian somatic cells.

Dronc phenotypes are strongly linked to Ptc-induced autophagy

The reported ability of Ptc to induce autophagy (Jimenez-Sanchez *et al*, 2012; Singh *et al*, 2018) and its prevalent accumulation in Atg8 autophagosomes in somatic cells without Dronc (Fig 5D–G) prompted us to evaluate the biological significance of autophagy in our cellular scenario. To assess the autophagy flux, we first used a dual GFP-mCherry-Atg8 reporter (Nezis *et al*, 2010). Whereas early autophagosomes are labelled with GFP and mCherry, late autophagolysosomes only retain the mCherry signal (Nezis *et al*, 2010; Appendix Fig S1A). This is due to the degradation of GFP caused by the acidification of autophagosomes upon fusing with the lysosomes (Nezis *et al*, 2010). The live observation of the GFP-mCherry-Atg8 reporter showed more abundant and larger autophagosomes in *ptc-Gal4:Dronc* germaria (Fig 6A–C). Interestingly, the proportion of early autophagosomes expressing GFP and mCherry also increased (Fig 6D). We then evaluated the expression of the autophagy marker known as Ref2P; Ref2P is degraded in cells with high levels of autophagy (Bjorkoy *et al*, 2009) and is the *Drosophila* orthologue of p62 (Nezis *et al*, 2008). Our experiments showed an accumulation of Ref2P in *ptc-Gal4*/+ germaria that was rescued by halving the dose of *Dronc* (*ptc-Gal4*/+; *Dronc*^{KO}/+; Fig 6E–G). Importantly, similar results were observed in human Caspase-9-deficient cells exposed to low concentrations of EtOH (Fig 6H and I); previous reports have shown that low levels of EtOH trigger moderate cellular stress and activation of autophagy (Li *et al*, 2014). We obtained further evidence regarding the specificity of p62 downregulation in these experiments by treating Caspase-9 deficient cells exposed to ETOH with bafilomycin, a well-characterised inhibitor of autophagy (Mauvezin & Neufeld, 2015). The bafilomycin treatment restored the intracellular levels of p62 (Fig 6H and I). These findings strongly connected caspase deficiency with the Ptc-dependent induction of autophagy.

To functionally assess the contribution of autophagy to Dronc phenotypes, we compromised the expression of Atg8 in FCs without Dronc. The reduction of Atg8 and therefore autophagy was

© 2023 The Authors

sufficient to sustain the proliferation and differentiation of FCs without Dronc (Fig 6J–L; compare with Fig 2C). Consistently, Atg1 deficiency also rescued the differentiation defects observed in *ptc-Gal4*/+; *Dronc*^{KO}/+ germaria (Appendix Fig S1B–D; compare with Fig 4J). These findings suggested that the autophagy excess caused by caspase deficiency is key to explaining the proliferation and differentiation defects detected in ovarian somatic cells under moderate thermal stress.

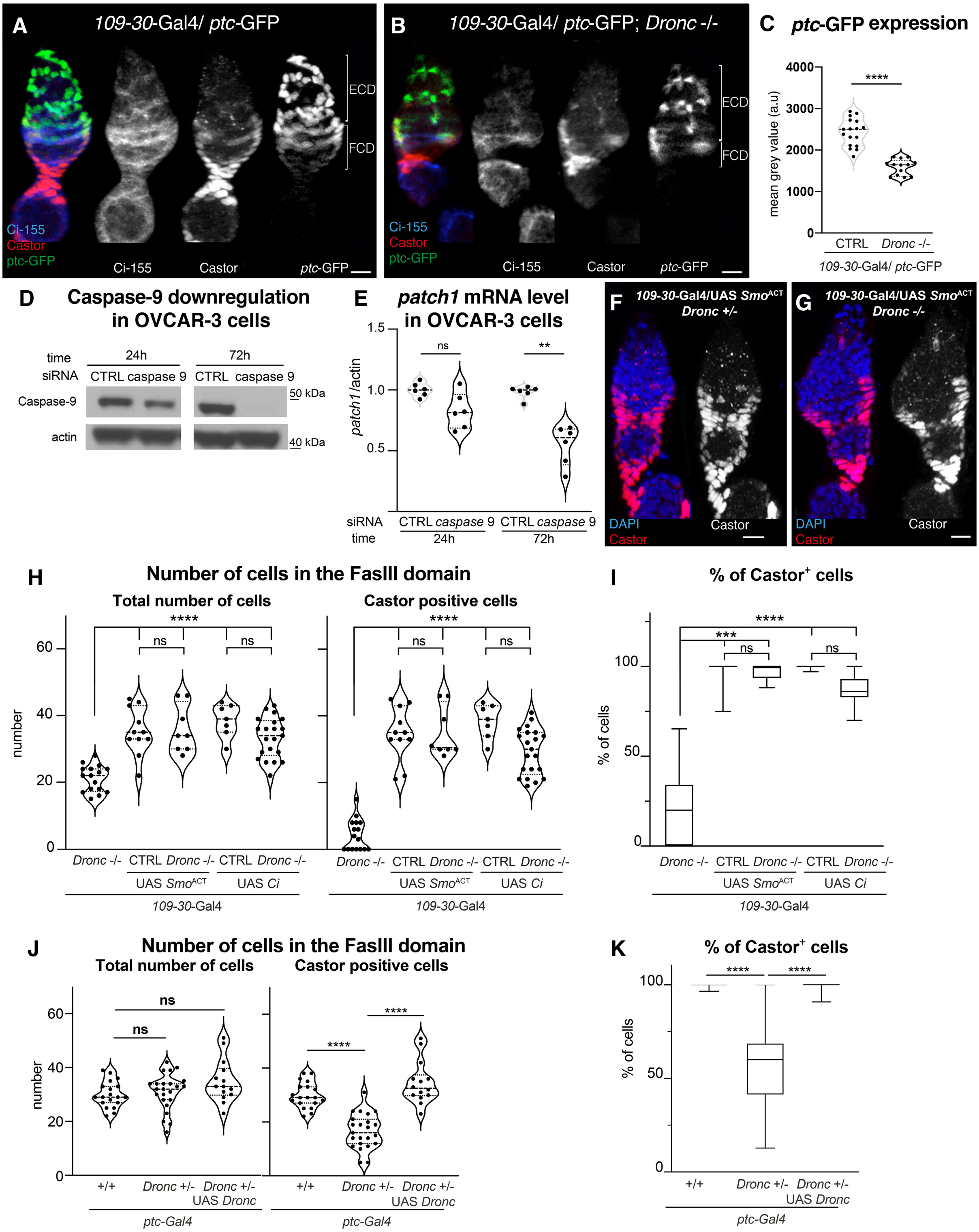

Figure 4.

Figure 4. *Dronc* deficiency reduces Hh-signalling in *Drosophila* and OVCAR-3 ovarian somatic cells.

A, B Representative 2D projections of confocal images showing the expression of Ci-155 (blue and grey channels), *ptc*-GFP (green and grey); *ptc*-GFP is a *bona-fide* transcriptional read-out of Hh pathway and weak hypomorph allele (Buszczak *et al*, 2007), and Castor (red and grey) in germaria with either normal (A) or deficient Dronc expression (B). Genotypes: *109-30-Gal4* (BL7023)/*ptc*-GFP^{CB02030} (a gift from Isabel Guerrero) (A) and *109-30-Gal4* (BL7023)/*ptc*-GFP^{CB02030}; *Dronc*^{KO}Tub-*G80*^{ts} (BL7019)/UAS-*flippase Dronc*$^{KO\text{-}FRT\text{-}Dronc\text{-}GFP\text{-}APEX\text{-}FRT\text{-}QF}$.

C Quantification of *ptc*-GFP expression in either control ($n = 17$) or *Dronc* mutant ($n = 13$) germaria shown in (A and B); an unpaired parametric Welch's *t*-test was used to establish the statistical significance (****$P \le 0.0001$).

D Western blot showing Caspase-9 expression (upper lane) and actin (bottom lane, loading control) in either control or *Caspase-9* deficient OVCAR-3 cells (24 and 72 h post-transfection of an shRNA against *Caspase-9*). Notice the strong downregulation of Caspase-9 72 h after siRNA treatment.

E mRNA levels of *patch1* measured by Q-PCR in either control or *Caspase-9* deficient OVCAR-3 cells; a nonparametric Mann–Whitney *t*-test was used to establish the statistical significance (**$P \le 0.01$). $N = 6$ biological replicates.

F, G Representative 2D projection of confocal images showing Castor expression (red and grey channels) in follicular cells either heterozygous (F) or homozygous (G) mutant for *Dronc* that express a constitutively active form of *smo*. DAPI staining labels the nuclei. Genotypes: *109-30-Gal4* (BL7023)/UAS-*smo*^{Act} (BL44621); *Dronc*$^{KOTub\text{-}G80ts}$ (BL7019)/+ (F). *109-30-Gal4* (BL7023)/UAS-*smo*^{Act} (BL44621); *Dronc*^{KO}Tub-*G80*^{ts} (BL7019)/UAS-*flippase* (BL8209) *Dronc*$^{KO\text{-}FRT\text{-}Dronc\text{-}GFP\text{-}APEX\text{-}FRT\text{-}QF}$ (G).

H Quantification of total number of follicular cells (left) or Castor-expressing cells (right) within the FasIII cellular domain in the following genotypes from left to right: *Dronc−/− = CTRL = 109-30-Gal4* (BL7023)/; *Dronc*^{KO}Tub-*G80*^{ts} (BL7019)/ UAS-*flippase* (BL8209) *Dronc*$^{KO\text{-}FRT\text{-}Dronc\text{-}GFP\text{-}APEX\text{-}FRT\text{-}QF}$ ($n = 16$). *CTRL = 109-30-Gal4* (BL7023)/UAS-*smo*^{Act} (BL44621); *Dronc*^{KO}Tub-*G80*^{ts} (BL7019)/+ ($n = 11$). Dronc−/− = 109-30-Gal4 (BL7023)/UAS-smo^{Act} (BL44621); Dronc^{KO}Tub-G80^{ts} (BL7019)/UAS-*flippase* (BL8209) Dronc$^{KO\text{-}FRT\text{-}Dronc\text{-}GFP\text{-}APEX\text{-}FRT\text{-}QF}$ ($n = 8$). *CTRL = 109-30-Gal4* (BL7023)/UAS-*Ci* (BL28984); *Dronc*^{KO}Tub-*G80*^{ts} (BL7019)/+. ($n = 7$). Dronc−/− = 109-30-Gal4 (BL7023)/UAS-UAS-Ci (BL28984); Dronc^{KO}Tub-G80^{ts} (BL7019)/UAS-*flippase* (BL8209) Dronc$^{KO\text{-}FRT\text{-}Dronc\text{-}GFP\text{-}APEX\text{-}FRT\text{-}QF}$ ($n = 21$). A Kruskal–Wallis test and Dunn's multiple comparison post-test were used to determine statistical significance (n.s., not significant; ****$P \le 0.0001$).

I Percentage of Castor-expressing cells versus the total number of Follicular cells (FasIII$^+$ cells) in germaria of the genotypes indicated in (H). A Kruskal–Wallis test and Dunn's multiple comparison post-test were used to determine statistical significance (n.s., not significant, ***$P \le 0.001$, ****$P \le 0.0001$).

J Quantification of total number of follicular cells (left) or Castor-expressing cells (right) within the FasIII cellular domain in the following genotypes from left to right: *Dronc+/+ = ptc-Gal4* (BL2017)/+; Tub-*G80*^{ts} (BL7019) ($n = 19$). *Dronc+/− = ptc-Gal4* (BL2017)/+; *Dronc*^{KO}Tub-*G80*^{ts} (BL7019)/+ ($n = 23$). *Dronc+/− UAS-Dronc = ptc-Gal4* (BL2017)/+; *Dronc*^{KO}Tub-*G80*^{ts} (BL7019)/UAS-*Dronc* (BL56198) ($n = 14$). A one-way ordinary ANOVA and Welch's correction were used to establish statistical significance (n.s., not significant; ****$P \le 0.0001$).

K Percentage of Castor-expressing cells versus the total number of Follicular cells (FasIII$^+$ cells) in germaria of the genotypes indicated in (J). A Kruskal–Wallis test and Dunn's multiple comparison post-test were used to determine statistical significance (****$P \le 0.0001$).

Data information: Scale bars represent 10 μm in the entire figure. Experimental flies were kept after eclosion from the pupae for 14 days at 29°C prior to dissection (applicable to all panels). Unless otherwise indicated, all experimental data presented were obtained from $N \ge 2$ biological replicates. The median and quartiles are indicated in the violin plots. The box plots show the median, first quartile and third quartile of datasets. The whiskers illustrate the range between the maximum and minimum values of datasets. All the quantifications were made in germaria containing one single group of germline cells wrapped by follicular cells.
Source data are available online for this figure.

Figure 5. *Dronc* deficiency facilitates the intracellular accumulation of Ptc in autophagosomes.

A Representative 2D projection of confocal images showing Ptc immunostaining (grey channel) in germaria of the following genotypes: *Dronc*^{KO}Tub-*G80*^{ts} (BL7019)/+. *ptc-Gal4* (BL2017)/+; Tub-*G80*^{ts} (BL7019)/+. *ptc-Gal4* (BL2017)/+; *Dronc*^{KO}Tub-*G80*^{ts} (BL7019)/+.

B Relative number of Ptc-positive puncta per germaria of the following genotypes, from left to right: Dronc^{KO} Tub-G80^{ts}/+ ($n = 10$). ptc-Gal4/+; Tub-G80^{ts}/+ ($n = 9$). ptc-Gal4/+; Dronc^{KO} Tub-G80^{ts}/+ ($n = 10$). A Kruskal–Wallis test and Dunn's multiple comparison post-test were used to determine statistical significance (***$P \le 0.001$).

C Western blot showing Ptc (upper lane) and actin (bottom lane, loading control) expression in ovaries of the genotypes shown in (A). Notice the Ptc accumulation in double heterozygous germaria (*ptc-Gal4*/+; *Dronc*^{KO} *Tub-G80*^{ts}/+).

D Representative 2D projection of confocal images showing Ptc (green) and Atg8 immunostainings (red) in germaria of the following genotypes: *ptc-Gal4* (BL2017)/UAS-*GFP-mCherry-Atg8* (BL37749). *ptc-Gal4* (BL2017)/UAS-*GFP-mCherry-Atg8* (BL37749); *Dronc*^{KO}Tub-*G80*^{ts} (BL7019)/+. Notice the higher colocalisation between the green and red signal in the germarium *ptc-Gal4* (BL2017)/+; *Dronc*^{KO}Tub-*G80*^{ts} (arrows).

E Pearson colocalisation index between Ptc and Atg8 in germaria of the following genotypes: *ptc-Gal4* (BL2017)/UAS-*GFP-mCherry-Atg8* (BL37749) ($n = 12$). *ptc-Gal4* (BL2017)/UAS-*GFP-mCherry-Atg8* (BL37749); *Dronc*^{KO}Tub-*G80*^{ts} (BL7019)/+ ($n = 18$). An unpaired parametric Welch's *t*-test was used to establish the statistical significance (**$P \le 0.01$).

F Fraction of Ptc positive that overlaps with Atg8 in germaria of the genotypes shown in (E). *n* number is indicated in (E). An unpaired parametric Welch's *t*-test was used to establish the statistical significance (***$P \le 0.001$). The *Y* axis in the graph shows the Manders' Coefficient M1-M2.

G Fraction of Atg8+ autophagosomes that overlaps with Ptc in germaria of the genotypes shown in (E). *n* number is indicated in (E). An unpaired parametric Welch's *t*-test was used to establish the statistical significance (**$P \le 0.01$). The *Y* axis in the graph shows the Manders' Coefficient M2-M1.

H Quantification of total number of follicular cells (left) or Castor-expressing cells (right) within the FasIII cellular domain in the following genotypes from left to right: *CTRL = 109-30*-Gal4 (BL7023)/+; *Dronc*^{KO}Tub-*G80*^{ts} (BL7019)/+. ($n = 17$). Dronc−/− = 109–30Gal4 (BL7023)/+; Dronc^{KO}Tub-G80^{ts} (BL7019)/UAS-*flippase* (BL8209) Dronc$^{KO\text{-}FRT\text{-}Dronc\text{-}GFP\text{-}APEX\text{-}FRT\text{-}QF}$. ($n = 16$). *Dronc−/−* UAS-*ptc*-RNAi = *109–30Gal4* (BL7023)/UAS-*ptc*-RNAi (BL55686); *Dronc*^{KO}Tub-*G80*^{ts} (BL7019)/ UAS-*flippase* (BL8209) *Dronc*$^{KO\text{-}FRT\text{-}Dronc\text{-}GFP\text{-}APEX\text{-}FRT\text{-}QF}$. ($n = 15$). A Kruskal–Wallis test and Dunn's multiple comparison post-test were used to determine statistical significance (****$P \le 0.0001$).

I Percentage of Castor-expressing cells versus the total number of Follicular cells (FasIII$^+$ cells) in germaria of the genotypes indicated in (H). Data are expressed as box-and-whiskers plots, with min to max range as whiskers. A Kruskal–Wallis test and Dunn's multiple comparison post-test were used to determine statistical significance (***$P \le 0.001$; ****$P \le 0.0001$).

Data information: Scale bars represent 10 μm. Experimental flies were kept after eclosion from the pupae for 14 days at 29°C prior to dissection (applicable to all panels). All the experimental data shown have been obtained from $N \ge 2$ biological replicates. The median and quartiles are indicated in the violin plots. The box plot shows the median, first quartile and third quartile of the dataset. The whiskers illustrate the range between the maximum and minimum values of the dataset. All the quantifications were made in germaria containing one single group of germline cells wrapped by follicular cells.
Source data are available online for this figure.

© 2023 The Authors

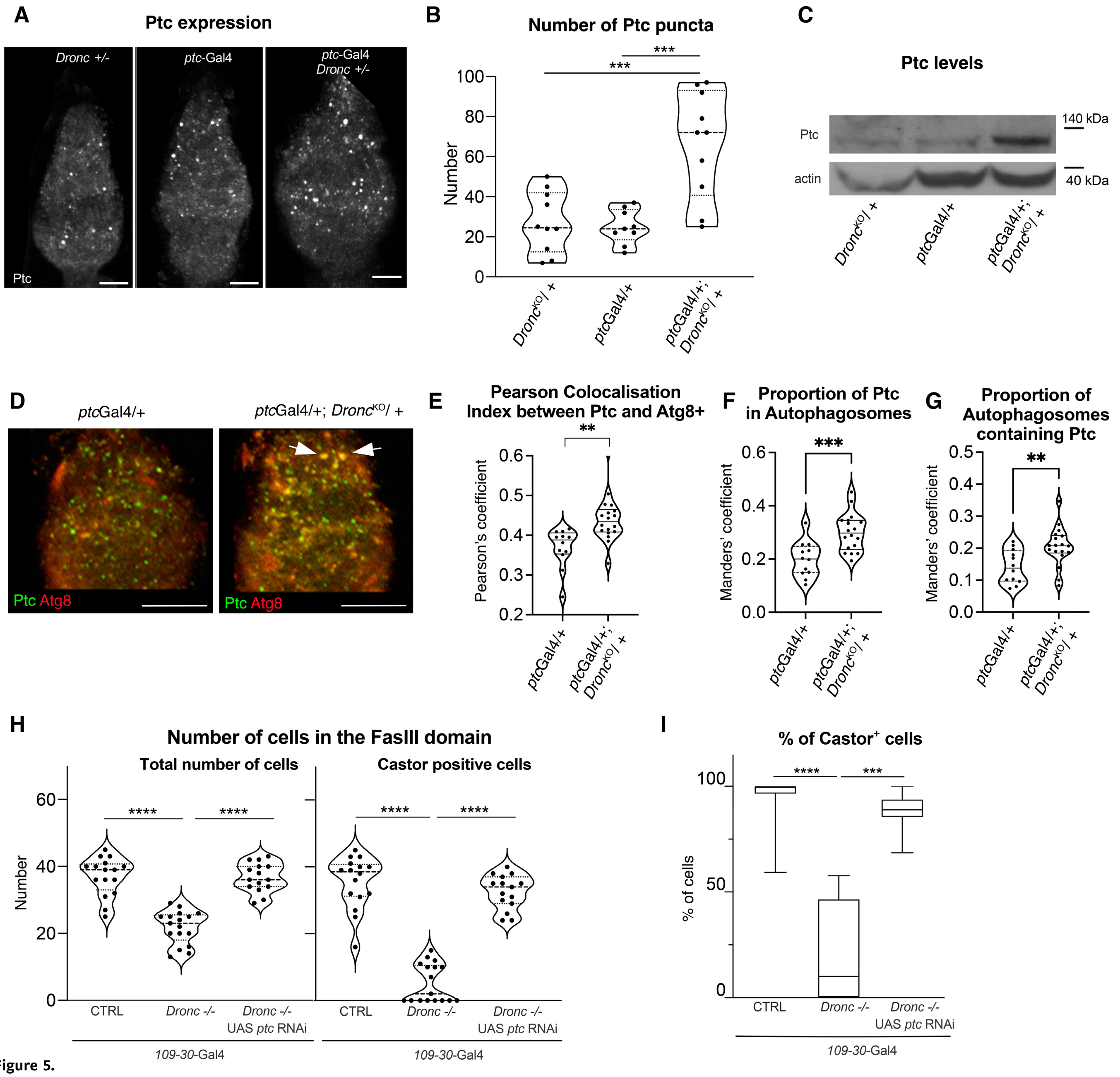

Figure 5.

Discussion

The caspases are the main drivers of apoptosis but also control numerous cellular processes without inducing cell death (Aram *et al*, 2017; Bell & Megeney, 2017; Baena-Lopez, 2018). However, our knowledge regarding the regulation and biological significance of non-apoptotic caspase functions is still limited. Here, we show that non-apoptotic caspase activation prevents autophagy excess and sustains Hh-signalling in ovarian somatic cells under moderate thermal stress. Importantly, these novel caspase functions are pro-survival since they facilitate cell proliferation and differentiation of ovarian somatic precursors and their progeny.

Caspase activation can support essential cellular functions without causing apoptosis

Our experiments have shown widespread expression and transient non-apoptotic activation of caspases in *Drosophila* ovarian somatic cells under moderate thermal stress (Figs 1 and EV1). Importantly, such caspase activation appears to sustain cell proliferation and differentiation (Fig 2). These findings strongly support a pro-survival role of moderate and transient caspase activation in ovarian somatic cells while cautioning against the generic association of caspase patterns with apoptosis (Ding *et al*, 2016; Sun *et al*, 2017). Interestingly, the regulation of cell proliferation and differentiation

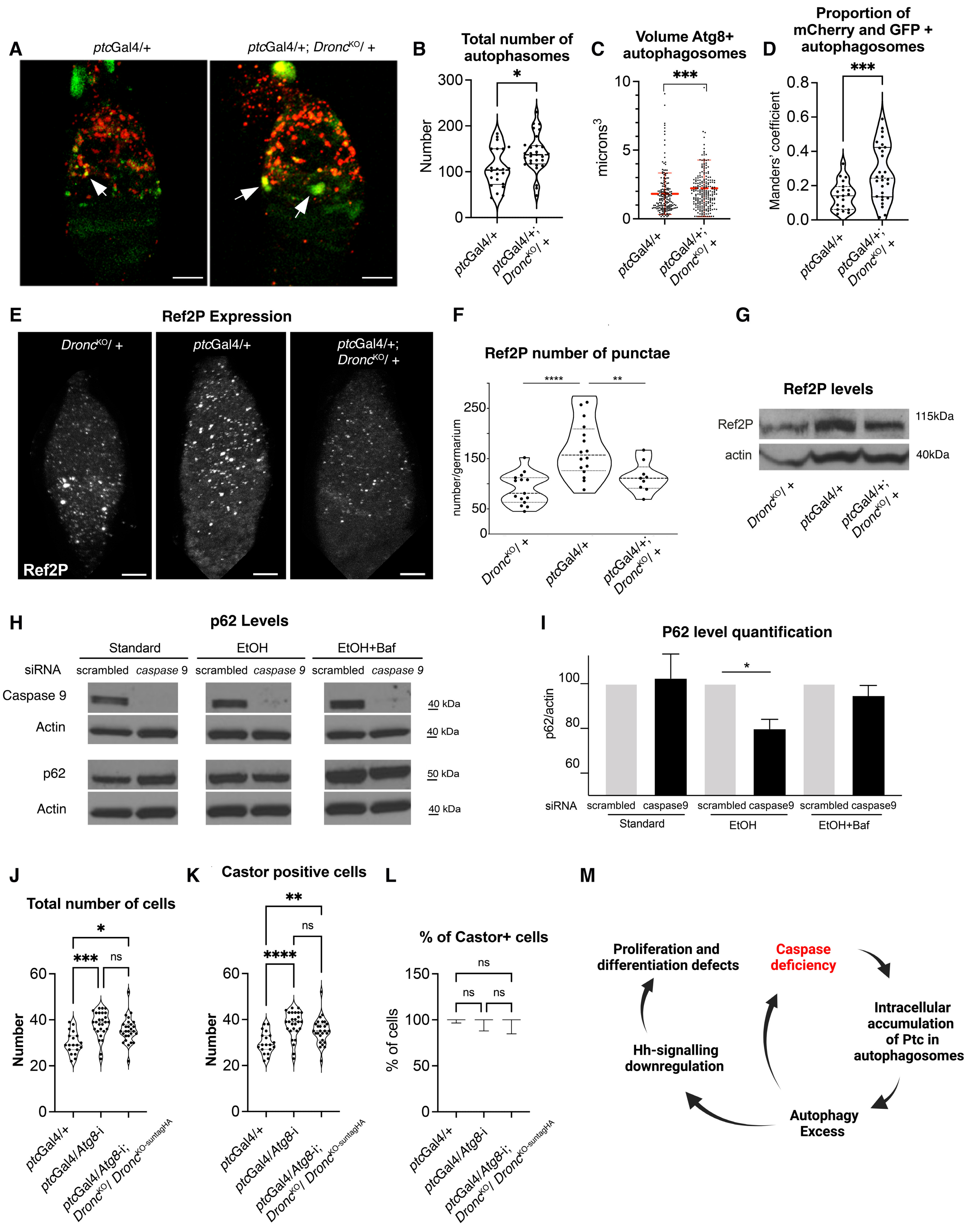

Figure 6.

© 2023 The Authors

Figure 6. *Dronc* deficiency phenotypes are strongly linked to an excess of autophagy induced by Ptc accumulation.

A Representative 2D projection of confocal images showing the overlap between GFP (green) and Cherry (red) of Atg8+ autophagosomes in germaria of the following genotypes: *ptc-Gal4* (BL2017)/UAS-*GFP-mCherry-Atg8* (BL37749). *ptc-Gal4* (BL2017)/ UAS-*GFP-mCherry-Atg8* (BL37749); *Dronc*^{KO}Tub-*G80*^{ts} (BL7019)/+. Notice the higher colocalisation between the green and red signals in the germarium with reduced Dronc expression (arrows) as well as the higher size of Atg8+ puncta.

B Total number of Atg8+ autophagosomes in germaria of the following genotypes: *ptc-Gal4* (BL2017)/UAS-*GFP-mCherry-Atg8* (BL37749) ($n = 21$). *ptc-Gal4* (BL2017)/UAS-*GFP-mCherry-Atg8* (BL37749); *Dronc*^{KO}Tub-*G80*^{ts} (BL7019)/+ ($n = 28$). An unpaired parametric Welch's *t*-test was used to establish the statistical significance (*$P \leq 0.05$).

C Volume of Atg8+ autophagosomes in germaria of the genotypes described in (B). *n* number is indicated in (B). A nonparametric Mann–Whitney *t*-test was used to determine statistical significance (***$P < 0.001$).

D Fraction of Cherry positive that overlaps with GFP in Atg8+ autophagosomes within germaria of the genotypes shown in (B). *n* number is indicated in (B). An unpaired parametric Welch's *t*-test was used to establish the statistical significance (***$P \leq 0.001$). The *Y* axis in the graph shows the Manders' Coefficient M1-M2.

E Representative 2D projection of confocal images showing Ref2P immunostaining (grey channel) in germaria of the following genotypes: *Dronc*^{KO}Tub-*G80*^{ts} (BL7019)/+. *ptc-Gal4* (BL2017)/+; Tub-*G80*^{ts} (BL7019)/+. *ptc-Gal4* (BL2017)/+; *Dronc*^{KO}Tub-*G80*^{ts} (BL7019)/+.

F Relative number of Ref2P-positive punctae per germaria of the following genotypes, from left to right: *Dronc*^{KO}Tub-*G80*^{ts} (BL7019)/+. ($n = 15$). *ptc-Gal4* (BL2017)/+; Tub-*G80*^{ts} (BL7019)/+. ($n = 16$). *ptc-Gal4* (BL2017)/+; *Dronc*^{KO}Tub-*G80*^{ts} (BL7019)/+. ($n = 9$). A Kruskal–Wallis test and Dunn's multiple comparison post-test were used to determine statistical significance (**$P \leq 0.01$, ****$P \leq 0.0001$).

G Western blot showing Ref2P (upper lane) and actin (bottom lane, loading control) in ovaries of the genotypes shown in (A). Notice the Ref2P reduction in double heterozygous germaria (*ptc-Gal4*/+; *Dronc*^{KO} *Tub-G80*^{ts}/+) compared with the (*ptc-Gal4*/+; *Tub-G80*^{ts}/+) control.

H Western blot showing the expression levels of the autophagy marker p62 (upper lane), Caspase-9 (middle lane) and Actin (bottom lane, loading control) in either scrambled or *Caspase-9* deficient OVCAR-3 cells; the protein levels of the different read-outs were measured at 72 h after siRNA treatment in cells grown during the last 4 h before sample processing in our standard cell culture conditions, in cell culture media containing EtOH (0.2%), and in cell culture media containing EtOH (0.2%) + bafilomycin A1 (400 nM).

I Quantification of p62 protein levels in the experimental conditions described in (H). one sample T Wilcoxon test was used to calculate statistical significance, *$P \leq 0.05$, $N \geq 3$. Bars indicate value of the mean while error bars represent the standard deviation.

J Quantification of total number of follicular cells within the FasIII cellular domain in the following genotypes: *ptc-Gal4*/+ = *ptc-Gal4* (BL2017)/+; Tub-*G80*^{ts} (BL7019)/+. ($n = 19$). *ptc-Gal4* /*Atg8*i = *ptc-Gal4* (BL2017)/UAS-*Atg8*RNAi (VDRC 109654); Tub-*G80*^{ts} (BL7019)/+. ($n = 27$). *ptc-Gal4*/*Atg8*i *Dronc*^{KO}/*Dronc*$^{KO\text{-suntag-HA-Cherry}}$ = *ptc-Gal4* (BL2017)/UAS-*Atg8*RNAi (VDRC 109654); *Dronc*^{KO}Tub-*G80*^{ts} (BL7019)/*Dronc*$^{KO\text{-FRT-Dronc-APEX-GFP-FRT-suntag-HA-Cherry}}$ ($n = 27$). A Kruskal–Wallis test and Dunn's multiple comparison post-test were used to determine statistical significance (n.s., not significant, *$P \leq 0.05$, ***$P \leq 0.001$).

K Quantification of the total number of Castor-expressing cells within the FasIII cellular domain in the genotypes described in (J). *n* number is indicated also in (J). An ordinary one-way ANOVA and Dunnett's multiple comparison post-test were used to determine statistical significance (n.s., not significant, **$P \leq 0.01$, ****$P \leq 0.0001$).

L Percentage of Castor-expressing cells versus the total number of Follicular cells (FasIII^{+} cells) in germaria of the genotypes indicated in (J). Data are expressed as box-and-whiskers plots, with min to max range as whiskers. A Kruskal–Wallis test and Dunn's multiple comparison post-test were used to determine statistical significance (n.s., not significant).

M Summary diagram illustrating the effects of caspase deficiency in ovarian somatic cells and their likely sequence of appearance based on our results. Diagram generated with BioRender.

Data information: Scale bars represent 10 μm. Experimental flies were kept after eclosion from the pupae for 14 days at 29°C prior to dissection (applicable to all panels). All the experimental data shown have been obtained from $N \geq 2$ biological replicates. The median and quartiles are indicated in the violin plots. The box plot shows the median, first quartile and third quartile of the dataset. The whiskers illustrate the range between the maximum and minimum values of the dataset. All the quantifications were made in germaria containing one single group of germline cells wrapped by follicular cells.
Source data are available online for this figure.

likely requires different levels of caspase activation. Supporting this hypothesis, the combined downregulation of all effector caspases causes more penetrant phenotypes than the individual deficiency of selected effector caspase members (Fig 3D–F). In parallel, our genetic manipulations have shown that the entire apoptotic pathway can be engaged for non-apoptotic purposes (Fig 3D–F). Importantly, this is not exclusive to *Drosophila* since similar observations have been reported in mammalian muscle precursors (Dehkordi *et al*, 2020). Collectively, these results support the hypothesis that low and transient levels of caspase activation can implement pro-survival functions while potent and sustained activation leads to apoptosis (Aram *et al*, 2017; Bell & Megeney, 2017; Burgon & Megeney, 2017; Baena-Lopez, 2018).

Non-apoptotic caspase activation prevents intracellular accumulation of Ptc and excess of autophagy

Our experiments have uncovered that caspase deficiency promotes the intracellular accumulation of Ptc receptor in autophagosomes under moderate thermal stress (Fig 5D–G); therefore, we propose that caspase activation limits the inclusion of Ptc into autophagosomes (Fig 6M). Importantly, this previously unrecognised caspase function appears to limit the Ptc-dependent induction of autophagy (Jimenez-Sanchez *et al*, 2012; Singh *et al*, 2018). Supporting this hypothesis, we have shown that either a reduction of Ptc protein levels (Fig 5H and I) or autophagy (Fig 6J–L) is sufficient to rescue the cell proliferation and differentiation defects caused by caspase deficiency in follicular cells (Fig 2). Although the key molecular substrate underlying these caspase functions remains elusive, our genetic experiments suggest that there must be a protein cleaved by effector caspases since either the lack of expression or activity of these caspase pathway components is sufficient to compromise the physiological properties of ovarian somatic cells (Fig 3D–F). The mutual antagonism between the apoptotic pathway and autophagy has repeatedly been described in the literature. Whereas caspase activation has been shown to cleave key autophagy components (e.g., the autophagy regulators Atg4 and Beclin are well chracterised Caspase-3 substrates; Betin & Lane, 2009; Luo & Rubinsztein, 2010; Nikoletopoulou *et al*, 2013), several members of the caspase pathway are often degraded in autophagosomes to promote cell survival (Nikoletopoulou *et al*, 2013). Our findings now indicate that caspase activation can also limit autophagy levels by controlling the accumulation of autophagy inducers such as Ptc (Fig 6M). Importantly, the interplay between caspases and autophagy could

© 2023 The Authors

be evolutionarily conserved in ovarian somatic cells of human origin (Fig 6H).

Non-apoptotic caspase activation facilitates Hh-signalling

The cell proliferation and differentiation phenotypes caused by caspase deficiency in the germarium are highly reminiscent of Hh-signalling deprivation. Consistently, either caspase or Hh deficiency can delay the progression of the cell cycle in ovarian somatic cells (preprint: Melamed *et al*, 2022; Fig 2I). Furthermore, both factors are key to sustaining the expression of somatic markers such as Castor and Eyes absent (Fig 2A–D; Chang *et al*, 2013). These findings strongly connected the phenotypes caused by caspase deficiency with Hh-signalling downregulation. Supporting this hypothesis, the ligand-independent hyperactivation of the Hh pathway (overexpressing a constitutively active form of Smo or Ci) was sufficient to fully rescue the homeostasis of ovarian somatic cells without caspase activity (Figs 4F–I and 6M). Importantly, Hh-signalling has previously been reported to inhibit autophagy in mammalian cells (Jimenez-Sanchez *et al*, 2012), and the Hh-dependent inhibition of autophagy is considered one of the main factors conferring drug resistance to transformed cells (Zeng & Ju, 2018). Taking into consideration all these factors, we suggest that the excess of autophagy is likely the primary defect caused by caspase deficiency, and this subsequently limits the efficient transduction of the Hh pathway (Fig 6M). In this scenario, it is conceivable that the excess of autophagy in ovarian somatic cells without caspase activity could promote the degradation of Hh pathway transduction components, thus limiting its signalling capacity (Fig 6M). Supporting this model, we have observed that the lack of autophagy is also sufficient to revert the proliferation and differentiation defects of FCs without caspase activation (Fig 6M). Irrespective of the ultimate molecular mechanism, the genetic evidence strongly supports that non-apoptotic caspase activation prevents autophagy excess while facilitating Hh-signalling in ovarian somatic cells.

Evolutionary implications of non-apoptotic caspase activation in ovarian somatic cells

Taking into consideration the non-apoptotic roles of ancient members of the caspase family (Lee *et al*, 2010; Dick & Megeney, 2013; Bell & Megeney, 2017), our findings may have evolutionary implications. Since Dronc can act as a pro-survival factor in ovarian somatic cells, our data support the hypothesis that caspases could initially sustain basic cellular processes, and only their inadvertent/persistent activation would lead to cell death (Dick & Megeney, 2013). From this perspective, these pro-apoptotic enzymes could act as pro-survival factors, thus inverting the widely held view regarding their most primitive function.

Materials and Methods

Fly strains and fly husbandry details

All fly strains used are described at www.flybase.bio.indiana.edu unless otherwise indicated. After 24 h of egg laying at 25°C, experimental specimens were raised at 18°C, thus enabling the repression of Gal4 activity through a Gal80^{ts}. This prevents lethality in our experiments during larval and pupal stages. After hatching, adults were then transferred from 18°C to 29°C until dissection time. At 29°C, the repression of Gal80^{ts} disappears, and therefore gene expression via Gal4 is elicited within specific cell subpopulations.

Detailed genotype description

A full description of experimental genotypes and fly lines can be found in Figure legends and Appendix Tables S1 and S2.

Immunohistochemistry

Adult *Drosophila* ovaries were dissected in ice-cold PBS. Immunostainings and washes were performed according to standard protocols (fixing in PBS 4% paraformaldehyde, washing in PBT 0.3% (0.3% Triton X-100 in PBS)). Primary antibodies used in our experiments were as follows anti-Castor (1:2,000; a gift from Alex Gould); rabbit anti-HA (1:1,000; Cell Signaling C29F4); mouse anti-β-Gal (1:500; Promega Z378B); chicken Anti-β-Gal (1:200, Abcam AB9361); Anti-FasIII (1:75, Hybridoma Bank 7G10); Anti-Ci-155-full length (1:50, Hybridoma Bank 2A1); Anti-Ptc (1:50, Hybridoma Bank Apa1); Anti-Ref2P (1:300, Abcam 178440); Anti-mCherry (1:50, invitrogen Cat #PA5-34974). Conjugated secondary antibodies (Molecular Probes) were diluted in 0.3% PBT and used in a final concentration (1:200): conjugated donkey anti-rabbit Alexa-Fluor-488 (A21206) or 555 (A31572) or 647 (A31573), conjugated donkey anti-mouse Alexa-Fluor-488 (A21202) or 555 (A31570) or 647 (A31571), conjugated goat anti-rat Life Technologies (Paisley, UK) Alexa-Fluor-488 (A21247) or 555 (A21434). The detection of biotinylated proteins was made using Streptavidin conjugated with the 488 fluorophore (1:500; S11223). DAPI was added to the solution with the secondary antibodies for labelling the nuclei (1:1,000; Thermo Scientific 62248). Following incubation in secondary antibodies, samples were washed several times during 60 min in PBT. Finally, they were mounted on Poly-Prep Slides (P0425-72EA, Sigma) in Aqua-Poly/Mount (Polysciences, Inc (18606)).

TUNEL staining

Follicles from adult *Drosophila* females were dissected in ice-cold PBS and fixed in PBS containing 4% formaldehyde for 20′. After fixation, the samples were washed three times for 15′ with PBS and subsequently permeabilised with PBS containing 0.3% triton and 0.1% sodium citrate for 8′ on ice. Next we performed three washes for 20′ in PBS. The *in situ* detection of fragmented genomic DNA was performed according to the DeadEnd colorimetric TUNEL (Terminal transferase-mediated dUTP nick-end labelling) system (Promega). Briefly, samples were first equilibrated at room temperature in equilibration buffer (5–10′) and then incubated with TdT reaction mix for 1 h at 37°C in a humidified chamber to obtain the 3′-end labelling of fragmented DNA. The reaction was terminated with three washes for 15′ in PBS. If necessary, the TUNEL protocol was followed by standard immunofluorescent staining. The detection of TUNEL-positive cells was

 © 2023 The Authors

achieved by an incubation of 45′ with streptavidin-fluorophore conjugated dyes.

EdU staining

Adult female ovaries were dissected in 1× PBS, transferred to a microfuge tube containing 10 mM EdU in 1× PBS, and kept at room temperature on a shaker for 1 h. Ovarioles were then dissociated, fixed and stained with primary and secondary antibodies as described above. The EdU detection reaction was performed according to the manufacturer's manual (Thermo Fisher Scientific, C10640).

Generation of morphogenetic mosaics

Two-day-old adult females of the genotype yw hs-*Flp*$^{1.22}$/+; UAS-*flippase*/+; FRT80, *Dronc*^{l29}/ FRT80 Ubiquitin-*GFP* were given either two or four 1-h heat shocks at 37°C spread over 2 days (12 h apart). This allowed variable mitotic recombination efficiency and therefore different number of genetic mosaics. The higher is the number of heat shocks, the larger is the probability of covering a large fraction of tissue with mutant cells. After the last heat shock, flies were kept at 29°C under a regime of frequent transfer (every 2 days) to a fresh vial with standard food supplemented with yeast. Flies were dissected and immunostained 7 days after the last heat shock.

Imaging

Drosophila ovarioles were imaged using the Olympus Fluoview FV1200 and associated software. Z-stacks were taken with a 40× objective at intervals along the apical-basal axis that ensured adequate resolution along *Z*-axis (step size 0.5–1.5-μm). The same confocal settings were used during the image acquisition process of experimental and control samples. Acquired images were processed using ImageJ 1.52n, Adobe Photoshop2020 and Adobe Illustrator 2020 in order to complete the figure preparation. When confocal images were rotated, a dark rectangular background was added to create regularly shaped figures. The drawings of the manuscript corresponding to the synopsis, Figs EV2A and B, 6M, and Appendix Fig S1 were generated by Irina Stefana using the BioRender software. Biorender Figure licences can be provided upon request.

Ovaries expressing the transgene GFP-mCherry-Atg8 of the genotypes indicated in the figure (Fig 6A–D) were dissected and dissociated with dissection forceps over an ice-cold slide coated with polylysine (P0425-72EA). After the dissection was completed, two small coverslips (25/25 mm) were placed at both sides of the dissected germaria. Then, a large coverslip (25/50 mm) was placed on top of the specimens to facilitate the live imaging for no longer than 20 min under the microscope indicated above. The microscope was maintained at RT during image acquisition.

Image quantification

All of the images used in this study were randomised and blindly scored during the quantification process. Images for quantification purposes were processed with ImageJ 1.52p.

The total number of cells expressing either FasIII or UAS-Histone-RFP in the follicular region of the germarium was manually quantified in approximately 60 different confocal planes comprising the entire volume of the germarium using the ImageJ Cell Counter plug-in and the following protocol. Using the Cell Counter plug-in, we permanently label each follicular cell identified in a given focal plane with a solid dot. In subsequent focal planes, these dots appear in the same position as empty circles, thus avoiding the repeated counting of the same object. New dots can be added throughout the entire Z-stack, in each focal plane (please see an example of the procedure in Appendix Fig S2). This procedure was followed to estimate the number of cells within the follicular region also expressing other markers (e.g., Castor). The percentage of Castor-expressing cells in the follicular region was estimated dividing the total number of Castor-expressing cells by the total number of FasIII/UAS-Histone-RFP-positive cells in the follicular region.

To quantify the number and size of Ptc and Ref2P-positive particles in the germarium, we first made a maximum projection of the total focal planes. Then, we sequentially applied the thresholding and "Analyse Particles" plug-ins from ImageJ. An equivalent image processing method was used to estimate the levels of Ptc expression based on the GFP signal. The "mean grey value" function of image J was used in this instance to estimate the GFP levels.

To obtain Pearson's colocalisation and Mander's coefficients between Ptc and Atg8 as well as GFP and mCherry, we processed the confocal images with Fiji and applied the Fiji plug-in named JACoP. Channels of interest were first separated and transformed into black-and-white images 8-bit images. Then, they were merged and the area outside of the germarium was eliminated using the Fiji command "Clear outside." Then, we opened the Fiji plug-in JACoP and thresholded the images with the tool of this software dedicated to that function. We also limit the particle size (1 to 7) using the "Obj. function." Finally, we clicked the Analyse button to obtain the results.

To calculate the volume of Atg8 autophagosomes, the channel showing the mCherry signal was separated and transformed into an 8-bit black-and-white image in Fiji. Then, we used the "3D Objects counter" Fiji plug-in to threshold and measure the particle volume.

Western blot

Adult *Drosophila* ovaries were dissected in ice-cold PBS and snap-frozen in liquid nitrogen. Subsequently, they were homogenised in NP40 buffer (150 mM NaCl, 50 mM Tris–HCl pH 7.5, 5% glycerol, 1% IGEPAL CA-630). Cells were harvested using trypsin/EDTA and centrifuged at 300 *g* for 5′. Pellets were washed in PBS and then treated with RIPA lysis buffer 1× (150 mM NaCl, 50 mM Tris–HCl pH 7.5, 0.1 mM EGTA, 0.5 mM EDTA, 1% Triton X-100). Halt Protease and Phosphatase Inhibitor Cocktail (Thermo Scientific Pierce) and Benzonase (BaseMuncher, Expedeon) were added according to the manufacturer's instructions. Protein content was determined using Bradford reagent (Bio-Rad). Extracts were mixed with NuPAGE LDS Sample Buffer and separated by SDS-PAGE. For performing the SDS-PAGE electrophoresis, lysates were loaded and run in NuPAGE Bis-Tris Gels in NuPAGE MOPS SDS Running Buffer (Thermofisher Scientific). Protein blot transfers were performed using Trans-Blot Turbo Transfer System (Bio-Rad). Nitrocellulose blots were incubated at room temperature for 30′ in blocking buffer

© 2023 The Authors

(Tris-buffered saline with 0.1% Tween containing 5% nonfat dried milk) and then incubated overnight at 4°C in the same blocking solution with the corresponding antibodies. After washing three times for 15′ each with Tris-buffered saline containing 0.1% Tween, the blots were incubated with horseradish peroxidase-conjugated (HRP) IgG, followed by washing. Immunoreactive bands were detected using the SuperSignal West Pico PLUS Chemiluminescent Substrate (Thermofisher Scientific). Developed CL-XPosure films (Thermofisher Scientific) were scanned using a flat-bed scanner, and the density of the bands was measured using Gel Analyzer plug-in in ImageJ software. Primary antibodies used: Anti-Ptc (1:500, Hybridoma Bank Apa1); Anti-Ref2P (1:500, Abcam 178440); Anti-Actin (1:500, Hybridoma Bank JLA20s); Anti-Ci-155-full length (1:500, Hybridoma Bank 2A1); Anti-Caspase-9 (C9) (1:1,000, Cell Signalling 9508); Anti-β-Actin—Peroxidase (1:20,000, Sigma A3854), Anti SQSTM1/P62 antibody (1:5,000, GeneTex GTX111393).

Cell culture mammalian cells

OVCAR-3 cells were maintained in RPMI (Sigma, R8758), supplemented with 10% FBS (Life Technologies, 10500064) and grown at 37°C in a humidified atmosphere with 5% CO_2. For the experiment shown in Fig 5C and D, we replaced the media with fresh media containing either EtOH (0.2%) or EtOH (0.2%) + the inhibitor of autophagy bafilomycin A1 (400 nM, Merck Chemicals). Cells were grown in these two different cell culture media during the last 4 h prior to sample processing.

RNA interference

Small interfering RNA (siRNA) specific for Caspase-9 (ON-TARGETplus SMART pool human L-003309-00-0005, 842), PTCH1 (ON-TARGETplus Human PTCH1, L-003924-00-0005, 5727) and nontargeting controls (ON-TARGET plus Non-targeting Pool, D-001810-10-05) were purchased from Dharmacon Inc. (UK). Cells were plated and transfected the day after with Oligofectamine™ Transfection Reagent (Thermofisher 12252) in the presence of siRNAs according to the manufacturer's instructions. Cells were kept in the transfection mix before processing for western blot or Q-PCR at the specified time points (24 and 72 h).

Gene expression analyses by Q-PCR

RNA extraction was performed using the Qiagen RNeasy Plus kit (74034). cDNAs were synthesised with Maxima First Strand cDNA synthesis kit (Molecular Biology, Thermofisher, K1642) Q-PCR were performed using QuantiNova SYBR Green PCR Kit (Qiagen, 208054). Detection was performed using Rotor-Gene Q Real-time PCR cycler (Qiagen).

Data were analysed using the Pfaffl method, based on $\Delta\Delta - Ct$ and normalised to actin as the housekeeping gene.

Gene expression was estimated with the following primers:

Patched1:

Forward CCACGACAAAGCCGACTACAT

Reverse GCTGCAGATGGTCCTTACTTTTTC

B-actin:

Forward CCTGGCACCCAGCACAAT

Reverse GGGCCGGACTCGTCATAC

Data availability

No primary datasets have been generated and deposited.

Expanded View for this article is available online.

Acknowledgements

Thanks for providing flies and reagents to Isabel Guerrero (*ptc-GFP*; Centro de Biología Molecular); Pascal Meier (UAS-*Dronc*-RNAi, UAS-*Drice*-RNAi, UAS-*Dcp*-RNAi, UAS-*Damm*-RNAi and UAS-*Decay*-RNAi); Iswar Hariharan (UAS-*miRGH*); Alex Gould (anti-Castor antibody, CRICK Institute), Masayuki Miura (*Dronc*^{TurboID}), the Developmental Studies Hybridoma Bank (antibodies), Addgene (pCDNA3-*connexin-GFP-Apex2* plasmid), Bloomington Stock Center (fly strains), Kyoto Stock Center (fly strains), Vienna Drosophila Research Center (VDRC; fly strains) and DGRC (wild-type cDNA of *dronc*). Thanks to Genewiz and Bestgene for making the DNA synthesis and generating transgenic flies, respectively. Thanks also to Ulrike Gruneberg, Sonia Muliyil, Xavier Franch-Marro, Jordan Raff and the caspaselab members (https://www.caspaselab.com) for the critical reading of the manuscript and valuable suggestions. Some diagrams in the manuscript have been created with BioRender.com. This work has been supported by Cancer Research UK C49979/A17516, the John Fell Fund from the University of Oxford (162/001), the Edward Penley Abraham Research Funds (RF290 and RF286 (19)), and the John Fell Fund from the University of Oxford 162/001. DCX was a Ph.D. student supported by the NIH NIDCR Intramural Research Program as part of the NIH-Oxford/Cambridge Scholars Program. AG was a postdoctoral fellow of CRUK (C49979/A17516) during the execution of the project. CH was supported by funding from the Biotechnology and Biological Sciences Research Council (BBSRC; BB/M011224/1).

Author contributions

Alessia Galasso: Data curation; formal analysis; supervision; validation; investigation; visualization; methodology; writing – review and editing. **Derek Cui Xu:** Data curation; investigation; writing – review and editing. **Claire Hill:** Data curation; investigation; writing – review and editing. **Daria Iakovleva:** Investigation; writing – review and editing. **Maria Irina Stefana:** Resources; data curation; writing – review and editing. **Luis Alberto Baena-Lopez:** Conceptualization; data curation; formal analysis; supervision; funding acquisition; validation; investigation; visualization; methodology; writing – original draft; project administration; writing – review and editing.

Disclosure and competing interests statement

The authors declare that they have no conflict of interest.

References

Aram L, Yacobi-Sharon K, Arama E (2017) CDPs: caspase-dependent non-lethal cellular processes. *Cell Death Differ* 24: 1307 – 1310

Arthurton L, Nahotko DA, Alonso J, Baena-Lopez LA (2019) Non-apoptotic caspase-dependent regulation of enteroblast quiescence in *Drosophila*. *EMBO Rep* 21: e48892

Baena-Lopez LA (2018) All about the caspase-dependent functions without cell death. *Semin Cell Dev Biol* 82: 77 – 78

Baena-Lopez LA, Arthurton L, Bischoff M, Vincent JP, Alexandre C, McGregor R (2018) Novel initiator caspase reporters uncover previously unknown features of caspase-activating cells. *Development* 145: dev170811

 © 2023 The Authors

Bell RAV, Megeney LA (2017) Evolution of caspase-mediated cell death and differentiation: twins separated at birth. *Cell Death Differ* 24: 1359 – 1368

Betin VM, Lane JD (2009) Caspase cleavage of Atg4D stimulates GABARAP-L1 processing and triggers mitochondrial targeting and apoptosis. *J Cell Sci* 122: 2554 – 2566

Bjorkoy G, Lamark T, Pankiv S, Overvatn A, Brech A, Johansen T (2009) Monitoring autophagic degradation of p62/SQSTM1. *Methods Enzymol* 452: 181 – 197

Briscoe J, Therond PP (2013) The mechanisms of Hedgehog signalling and its roles in development and disease. *Nat Rev Mol Cell Biol* 14: 416 – 429

Burgon PG, Megeney LA (2017) Caspase signaling, a conserved inductive cue for metazoan cell differentiation. *Semin Cell Dev Biol* 82: 96 – 104

Buszczak M, Paterno S, Lighthouse D, Bachman J, Planck J, Owen S, Skora AD, Nystul TG, Ohlstein B, Allen A *et al* (2007) The carnegie protein trap library: a versatile tool for drosophila developmental studies. *Genetics* 175: 1505 – 1531

Chai J, Yan N, Huh JR, Wu JW, Li W, Hay BA, Shi Y (2003) Molecular mechanism of reaper-grim-hid-mediated suppression of DIAP1-dependent Dronc ubiquitination. *Nat Struct Biol* 10: 892 – 898

Chang YC, Jang AC, Lin CH, Montell DJ (2013) Castor is required for Hedgehog-dependent cell-fate specification and follicle stem cell maintenance in drosophila oogenesis. *Proc Natl Acad Sci USA* 110: E1734 – E1742

Dai W, Peterson A, Kenney T, Burrous H, Montell DJ (2017) Quantitative microscopy of the *Drosophila* ovary shows multiple niche signals specify progenitor cell fate. *Nat Commun* 8: 1244

Dehkordi MH, Tashakor A, O'Connell E, Fearnhead HO (2020) Apoptosome-dependent myotube formation involves activation of caspase-3 in differentiating myoblasts. *Cell Death Dis* 11: 308

Dick SA, Megeney LA (2013) Cell death proteins: an evolutionary role in cellular adaptation before the advent of apoptosis. *Bioessays* 35: 974 – 983

Ding AX, Sun G, Argaw YG, Wong JO, Easwaran S, Montell DJ (2016) CasExpress reveals widespread and diverse patterns of cell survival of caspase-3 activation during development in vivo. *Elife* 5: e10936

Fadiga J, Nystul TG (2019) The follicle epithelium in the drosophila ovary is maintained by a small number of stem cells. *Elife* 8: e49050

Godwin AK, Meister A, O'Dwyer PJ, Huang CS, Hamilton TC, Anderson ME (1992) High resistance to cisplatin in human ovarian cancer cell lines is associated with marked increase of glutathione synthesis. *Proc Natl Acad Sci USA* 89: 3070 – 3074

Hartman TR, Strochlic TI, Ji Y, Zinshteyn D, O'Reilly AM (2013) Diet controls drosophila follicle stem cell proliferation via Hedgehog sequestration and release. *J Cell Biol* 201: 741 – 757

Hartman TR, Ventresca EM, Hopkins A, Zinshteyn D, Singh T, O'Brien JA, Neubert BC, Hartman MG, Schofield HK, Stavrides KP *et al* (2015) Novel tools for genetic manipulation of follicle stem cells in the drosophila ovary reveal an integrin-dependent transition from quiescence to proliferation. *Genetics* 199: 935 – 957

Hay BA, Wolff T, Rubin GM (1994) Expression of baculovirus P35 prevents cell death in *Drosophila*. *Development* 120: 2121 – 2129

Hayashi Y, Yoshinari Y, Kobayashi S, Niwa R (2020) The regulation of drosophila ovarian stem cell niches by signaling crosstalk. *Curr Opin Insect Sci* 37: 23 – 29

Hsia EY, Gui Y, Zheng X (2015) Regulation of Hedgehog signaling by ubiquitination. *Front Biol (Beijing)* 10: 203 – 220

Huang J, Kalderon D (2014) Coupling of Hedgehog and hippo pathways promotes stem cell maintenance by stimulating proliferation. *J Cell Biol* 205: 325 – 338

Jimenez-Sanchez M, Menzies FM, Chang YY, Simecek N, Neufeld TP, Rubinsztein DC (2012) The Hedgehog signalling pathway regulates autophagy. *Nat Commun* 3: 1200

Johnson RL, Milenkovic L, Scott MP (2000) In vivo functions of the patched protein: requirement of the C terminus for target gene inactivation but not Hedgehog sequestration. *Mol Cell* 6: 467 – 478

Laws KM, Drummond-Barbosa D (2015) Genetic mosaic analysis of stem cell lineages in the drosophila ovary. *Methods Mol Biol* 1328: 57 – 72

Lee RE, Brunette S, Puente LG, Megeney LA (2010) Metacaspase Yca1 is required for clearance of insoluble protein aggregates. *Proc Natl Acad Sci USA* 107: 13348 – 13353

Leulier F, Ribeiro PS, Palmer E, Tenev T, Takahashi K, Robertson D, Zachariou A, Pichaud F, Ueda R, Meier P (2006) Systematic in vivo RNAi analysis of putative components of the drosophila cell death machinery. *Cell Death Differ* 13: 1663 – 1674

Li Y, Wang S, Ni HM, Huang H, Ding WX (2014) Autophagy in alcohol-induced multiorgan injury: mechanisms and potential therapeutic targets. *Biomed Res Int* 2014: 498491

Losick VP, Morris LX, Fox DT, Spradling A (2011) *Drosophila* stem cell niches: a decade of discovery suggests a unified view of stem cell regulation. *Dev Cell* 21: 159 – 171

Lu X, Liu S, Kornberg TB (2006) The C-terminal tail of the Hedgehog receptor patched regulates both localization and turnover. *Genes Dev* 20: 2539 – 2551

Luo S, Rubinsztein DC (2010) Apoptosis blocks Beclin 1-dependent autophagosome synthesis: an effect rescued by Bcl-xL. *Cell Death Differ* 17: 268 – 277

Mauvezin C, Neufeld TP (2015) Bafilomycin A1 disrupts autophagic flux by inhibiting both V-ATPase-dependent acidification and Ca-P60A/SERCA-dependent autophagosome-lysosome fusion. *Autophagy* 11: 1437 – 1438

Melamed D, Choi A, Reilein A, Kalderon D (2022) Spatial regulation of drosophila ovarian follicle stem cell division rates and cell cycle transitions. *bioRxiv* https://doi.org/10.1101/2022.06.22.497017 [PREPRINT]

Motzny CK, Holmgren R (1995) The Drosophila cubitus interruptus protein and its role in the wingless and Hedgehog signal transduction pathways. *Mech Dev* 52: 137 – 150

Muro I, Monser K, Clem RJ (2004) Mechanism of Dronc activation in drosophila cells. *J Cell Sci* 117: 5035 – 5041

Nezis IP, Simonsen A, Sagona AP, Finley K, Gaumer S, Contamine D, Rusten TE, Stenmark H, Brech A (2008) Ref(2)P, the *Drosophila melanogaster* homologue of mammalian p62, is required for the formation of protein aggregates in adult brain. *J Cell Biol* 180: 1065 – 1071

Nezis IP, Shravage BV, Sagona AP, Lamark T, Bjorkoy G, Johansen T, Rusten TE, Brech A, Baehrecke EH, Stenmark H (2010) Autophagic degradation of dBruce controls DNA fragmentation in nurse cells during late *Drosophila melanogaster* oogenesis. *J Cell Biol* 190: 523 – 531

Nikoletopoulou V, Markaki M, Palikaras K, Tavernarakis N (2013) Crosstalk between apoptosis, necrosis and autophagy. *Biochim Biophys Acta* 1833: 3448 – 3459

Nystul T, Spradling A (2010) Regulation of epithelial stem cell replacement and follicle formation in the *Drosophila* ovary. *Genetics* 184: 503 – 515

Obata F, Kuranaga E, Tomioka K, Ming M, Takeishi A, Chen CH, Soga T, Miura M (2014) Necrosis-driven systemic immune response alters SAM metabolism through the FOXO-GNMT axis. *Cell Rep* 7: 821 – 833

Perez-Garijo A, Martin FA, Morata G (2004) Caspase inhibition during apoptosis causes abnormal signalling and developmental aberrations in *Drosophila*. *Development* 131: 5591 – 5598

© 2023 The Authors

Ray A, Meng E, Reed E, Shevde LA, Rocconi RP (2011) Hedgehog signaling pathway regulates the growth of ovarian cancer spheroid forming cells. *Int J Oncol* 39: 797 – 804

Reilein A, Melamed D, Park KS, Berg A, Cimetta E, Tandon N, Vunjak-Novakovic G, Finkelstein S, Kalderon D (2017) Alternative direct stem cell derivatives defined by stem cell location and graded Wnt signalling. *Nat Cell Biol* 19: 433 – 444

Rojas-Rios P, Guerrero I, Gonzalez-Reyes A (2012) Cytoneme-mediated delivery of Hedgehog regulates the expression of bone morphogenetic proteins to maintain germline stem cells in *Drosophila*. *PLoS Biol* 10: e1001298

Rosales-Nieves AE, Gonzalez-Reyes A (2014) Genetics and mechanisms of ovarian cancer: parallels between *Drosophila* and humans. *Semin Cell Dev Biol* 28: 104 – 109

Sahai-Hernandez P, Nystul TG (2013) A dynamic population of stromal cells contributes to the follicle stem cell niche in the *Drosophila* ovary. *Development* 140: 4490 – 4498

Sahai-Hernandez P, Castanieto A, Nystul TG (2012) *Drosophila* models of epithelial stem cells and their niches. *Wiley Interdiscip Rev Dev Biol* 1: 447 – 457

Shi J, Jin Z, Yu Y, Zhang Y, Yang F, Huang H, Cai T, Xi R (2021) A progressive somatic cell niche regulates germline cyst differentiation in the *Drosophila* ovary. *Curr Biol* 31: 840 – 852

Shinoda N, Hanawa N, Chihara T, Koto A, Miura M (2019) Dronc-independent basal executioner caspase activity sustains Drosophila imaginal tissue growth. *Proc Natl Acad Sci USA* 116: 20539 – 20544

Shyamala BV, Bhat KM (2002) A positive role for patched-smoothened signaling in promoting cell proliferation during normal head development in *Drosophila*. *Development* 129: 1839 – 1847

Siegrist SE, Haque NS, Chen CH, Hay BA, Hariharan IK (2010) Inactivation of both Foxo and reaper promotes long-term adult neurogenesis in *Drosophila*. *Curr Biol* 20: 643 – 648

Singh T, Lee EH, Hartman TR, Ruiz-Whalen DM, O'Reilly AM (2018) Opposing action of Hedgehog and insulin signaling balances proliferation and autophagy to determine follicle stem cell lifespan. *Dev Cell* 46: 720 – 734

Slobodkin MR, Elazar Z (2013) The Atg8 family: multifunctional ubiquitin-like key regulators of autophagy. *Essays Biochem* 55: 51 – 64

Sun G, Guzman E, Balasanyan V, Conner CM, Wong K, Zhou HR, Kosik KS, Montell DJ (2017) A molecular signature for anastasis, recovery from the brink of apoptotic cell death. *J Cell Biol* 216: 3355 – 3368

Szkandera J, Kiesslich T, Haybaeck J, Gerger A, Pichler M (2013) Hedgehog signaling pathway in ovarian cancer. *Int J Mol Sci* 14: 1179 – 1196

Tang HL, Tang HM, Fung MC, Hardwick JM (2015) In vivo CaspaseTracker biosensor system for detecting anastasis and non-apoptotic caspase activity. *Sci Rep* 5: 9015

Vied C, Kalderon D (2009) Hedgehog-stimulated stem cells depend on non-canonical activity of the notch co-activator mastermind. *Development* 136: 2177 – 2186

Xu D, Wang Y, Willecke R, Chen Z, Ding T, Bergmann A (2006) The effector caspases drICE and dcp-1 have partially overlapping functions in the apoptotic pathway in *Drosophila*. *Cell Death Differ* 13: 1697 – 1706

Yang X, Jin N, Wang Y, Yao Y, Wang Y, Li T, Liu C, Yu T, Yin H, Zhang Z *et al* (2021) Macroautophagy supports sonic Hedgehog signaling by promoting Patched1 degradation. *Biochim Biophys Acta Mol Cell Res* 1868: 119124

Zeng X, Ju D (2018) Hedgehog signaling pathway and autophagy in cancer. *Int J Mol Sci* 19: 2279

Zeng C, Chen T, Zhang Y, Chen Q (2017) Hedgehog signaling pathway regulates ovarian cancer invasion and migration via adhesion molecule CD24. *J Cancer* 8: 786 – 792

Zhang Y, Kalderon D (2001) Hedgehog acts as a somatic stem cell factor in the *Drosophila* ovary. *Nature* 410: 599 – 604

Zielke N, Korzelius J, van Straaten M, Bender K, Schuhknecht GFP, Dutta D, Xiang J, Edgar BA (2014) Fly-FUCCI: a versatile tool for studying cell proliferation in complex tissues. *Cell Rep* 7: 588 – 598

License: This is an open access article under the terms of the Creative Commons Attribution License, which permits use, distribution and reproduction in any medium, provided the original work is properly cited.

 © 2023 The Authors

Expanded View Figures

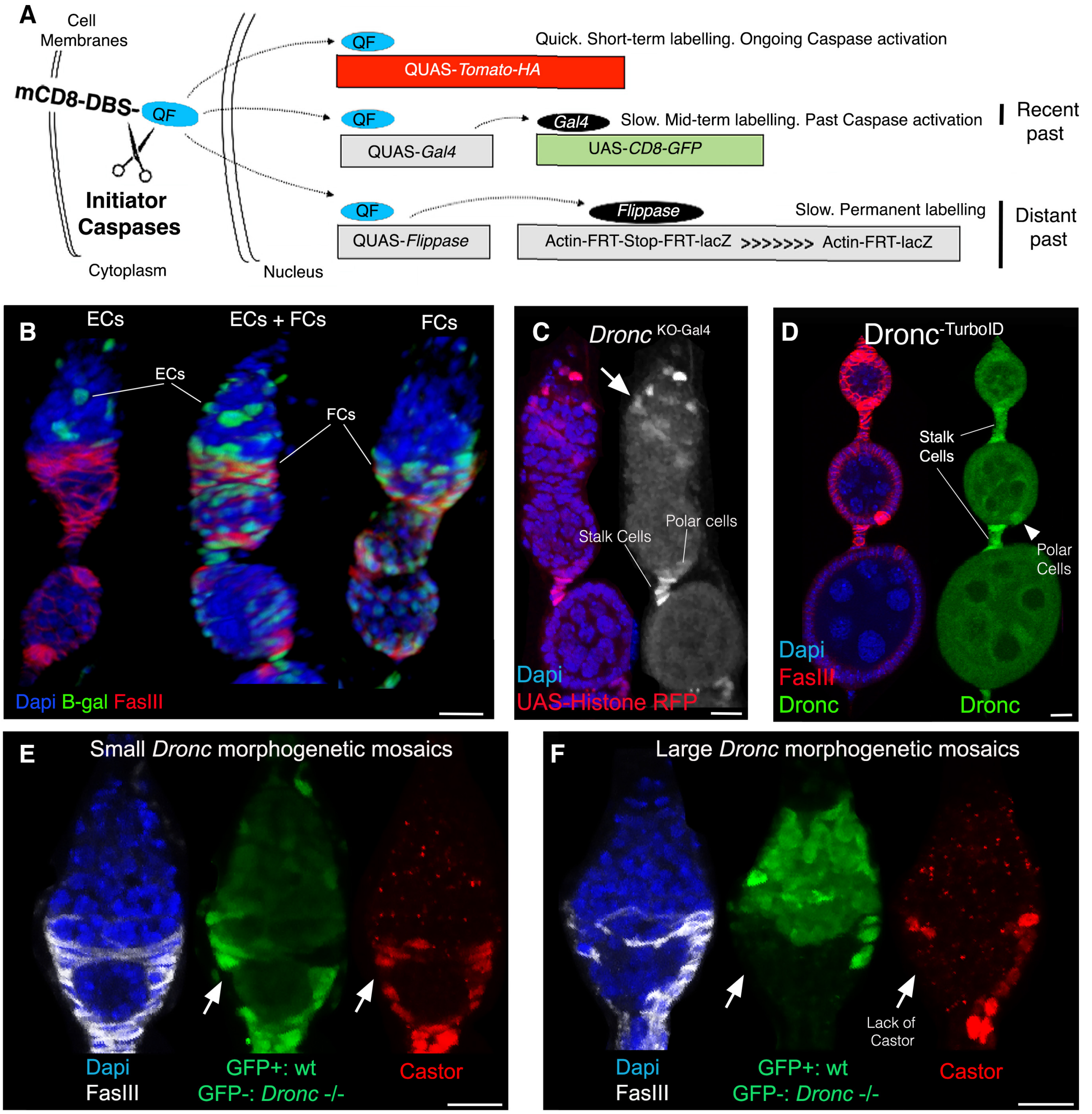

Figure EV1.

◀ **Figure EV1. Non-apoptotic caspase activation patterns detected in the germarium are connected to Dronc.**

A Schematic diagram illustrating the temporal caspase activation profile obtained using Drice-based sensor (DBS-S-QF). Left: schematic representation of the membrane attached mCD8-DBS-QF sensor. Right: different labelling systems used in combination with mCD8-DBS-QF to visualise the temporal patterns of initiator caspase activation. The early and ongoing view of initiator caspase activation is obtained through the expression of QUAS-*tomato-HA*. In parallel to the expression of tomato-HA, the QF translocation into the nucleus upon caspase-mediated cleavage can also activate the expression of the Gal4 transcription factor (QUAS-*Gal4*) and the recombinase known as Flippase (QUAS-*flippase*). Gal4 production can subsequently promote the transcription of a second cellular marker (UAS-*CD8-GFP*). Notice that the appearance of the GFP is temporarily delayed with respect to the tomato-HA signal since it demands a second transcriptional step. However, the GFP signal lasts for longer than the tomato-HA upon the caspase-mediated release of QF ceases due to the second transcriptional amplification loop obtained with the Gal/UAS system. Permanent labelling of caspase-activating cells (lineage tracing) can be achieved with the production of the Flippase recombinase. This enzyme facilitates the genomic excision of an FRT-stop cassette that prevents the expression of a nuclear β-galactosidase (β-gal) from the *lacZ* gene under the control of the actin promoter. Upon cassette excision, the production of β-gal stays forever in cells alive (permanent labelling).

B Representative 3D projections of confocal images showing the permanent labelling of different regions in the germarium (green, β-gal) obtained with DBS-S-QF. Nuclei are labelled with DAPI staining (blue). FasIII immunostaining labels the somatic follicular cells (red). Genotype: Actin *DBS-S-QF*, UAS-*mCD8-GFP*, QUAS-*tomato-HA*/+; QUAS-*flippase* (BL30126)/+; Actin5C FRT-*stop*-FRT *lacZ*-nls/+ (BL6355).

C Representative 2D projection of confocal images showing the expression pattern of *Dronc*^{KO}-*Gal4* at 29°C 10 days after adult eclosion from the pupae. UAS-*Histone-RFP* reports on Gal4 transcription (red and grey). DAPI stains the nuclei (blue). Notice the expression of *Dronc* in somatic cells of the germarium (arrows) as well as germarium the stalk and polar cells. Genotype: w;; *Dronc*$^{KO\text{-}Gal4}$/UAS-*Histone-RFP* (BL56555).

D Biotinylation signal (green) generated in the germarium by a *Dronc*-TurboID allele; notice the signal enrichment in stalk cells (white arrows) and polar cells (white arrowhead). FasIII (red) and DAPI (blue) stainings label the somatic cells and the nuclei, respectively. Experimental flies were kept after eclosion from the pupae for 10 days at 29°C prior to dissection. Genotype: *Dronc*::V5::TurboID/+ (a gift from Masayuki Miura).

E, F Expression of Castor (red) and FasIII (grey) in mutant morphogenetic mosaics for *Dronc*^{I29} in the germarium (GFP negative cells). Notice the downregulation of Castor (red, white arrows). Genotype: *yw hs-flippase*$^{1.22}$/+; FRT80 *Dronc*^{I29}/FRT80 Ubi*GFP*.

Data information: Scale bars represent 10 μm in the entire figure. Full description of genotypes for all of the EV Figures can be found in Appendix Table S2.

 © 2023 The Authors

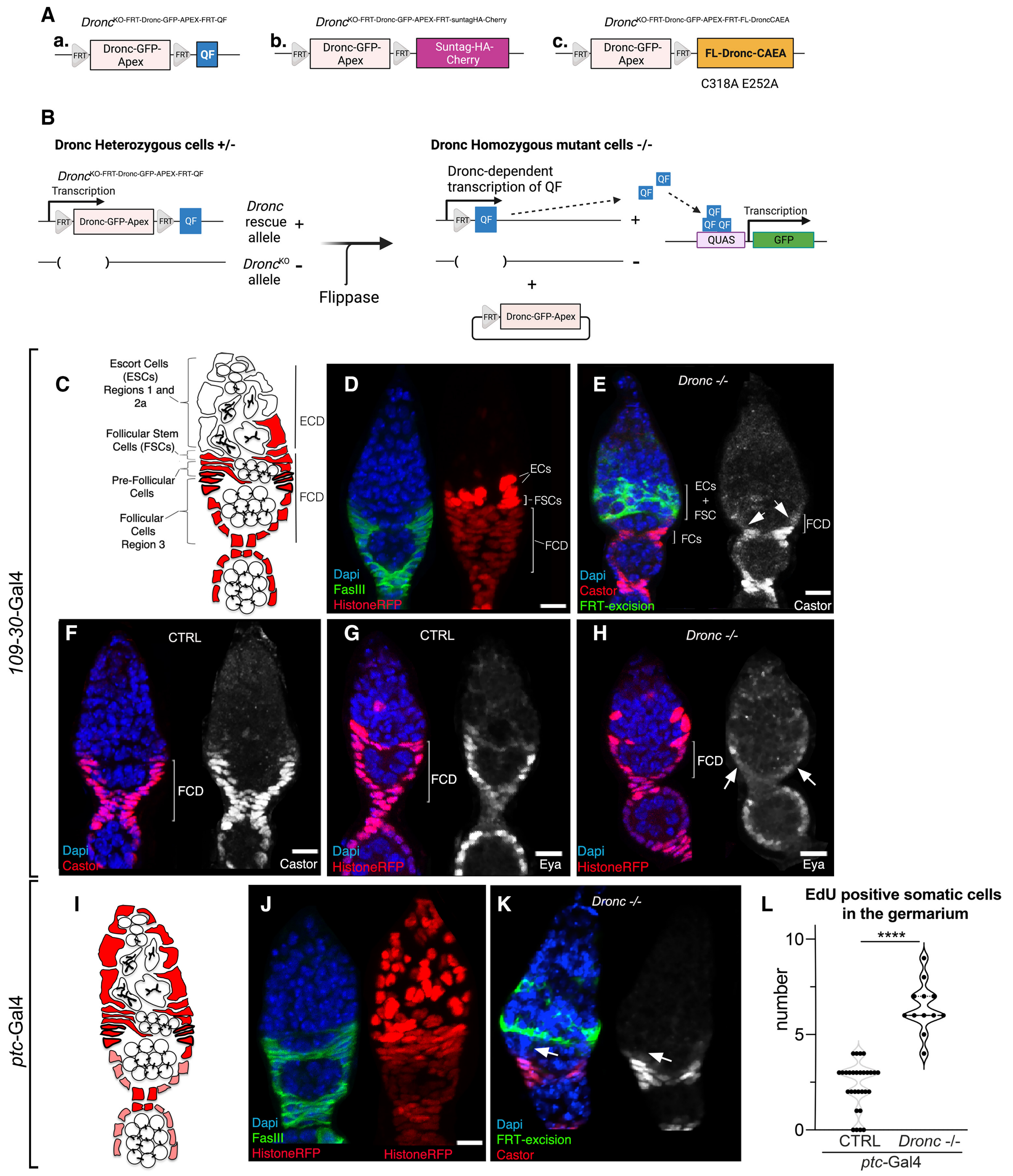
A
a. Dronc KO-FRT-Dronc-GFP-APEX-FRT-QF
FRT Dronc-GFP-Apex FRT QF
b. Dronc KO-FRT-Dronc-GFP-APEX-FRT-suntagHA-Cherry
FRT Dronc-GFP-Apex FRT Suntag-HA-Cherry
c. Dronc KO-FRT-Dronc-GFP-APEX-FRT-FL-DroncCAEA
FRT Dronc-GFP-Apex FRT FL-Dronc-CAEA
C318A E252A
B
Dronc Heterozygous cells +/-
Dronc Homozygous mutant cells -/-
Transcription
Dronc rescue allele +
Dronc KO allele -
Flippase
Dronc-dependent transcription of QF
QUAS
GFP
C
Escort Cells (ESCs) Regions 1 and 2a
Follicular Stem Cells (FSCs)
Pre-Follicular Cells
Follicular Cells Region 3
ECD
FCD
D
ECs
FSCs
Dapi
FasIII
HistoneRFP
E
Dronc -/-
ECs + FSC
FCs
Castor
FRT-excision
F
CTRL
G
Eya
H
109-30-Gal4
ptc-Gal4
I
J
K
L
EdU positive somatic cells in the germarium
number
****
CTRL
Dronc -/-
ptc-Gal4

Figure EV2.

Figure EV2. Expression pattern of relevant Gal4 drivers used in the manuscript.

A Schematics depicting the conditional *Dronc* alleles used throughout the manuscript. (a) the *Dronc*$^{KO\text{-}FRT\text{-}Dronc\text{-}GFP\text{-}ApexFRT\text{-}QF}$ allele expresses the transcriptional activator QF under the physiological regulation of *Dronc* upon FRT-rescue cassette excision; (b) the *Dronc*$^{KO\text{-}FRT\text{-}Dronc\text{-}GFP\text{-}ApexFRT\text{-}suntagHA\text{-}Cherry}$ allele expresses a chimeric Suntag-HA-Cherry polypeptide upon FRT-rescue cassette excision; (c) the *Dronc*$^{KO\text{-}FRT\text{-}Dronc\text{-}GFP\text{-}ApexFRT\text{-}FL\text{-}DroncCAEA}$ allele can express a full-length version of Dronc that has mutated the cysteine in the catalytic pocket C318A and the glutamic acid required for Dronc activation E352A. Diagram generated with BioRender.

B Diagram illustrating the allele conversion of Dronc heterozygous cells into homozygous mutants using the allele *Dronc*$^{KO\text{-}FRT\text{-}Dronc\text{-}GFP\text{-}ApexFRT\text{-}QF}$. Notice the Dronc-QF-dependent induction of a QUAS-CD8-GFP transgene upon Flippase-mediated excision of the FRT-rescue cassette. Diagram generated with BioRender.

C Schematic depicting the lineage tracing of *109-30*-Gal4 expressing cells (coloured in red) in the germarium at 29°C. Follicular Stem Cells (FSCs), pre-follicular cells (Pre-FCs) and adjacent Escort cells (ECs) are indicated.

D Representative 2D projection of confocal images showing a germarium expressing UAS-*Histone-RFP* (red) under the regulation of *109-30*-Gal4 driver during 14 days at 29°C; Castor (green) and DAPI (blue) label the follicular cells and the nuclei, respectively. Genotype: *109-30*-Gal4 (BL7023)/+; UAS-*Histone-RFP* (BL56555) Tub-*G80*^{ts} (BL7019)/+.

E Castor expression (red, grey and white arrows) in follicular cells without Dronc (green). Genotype 109-30-Gal4/QUAS-CD8-GFP; *Dronc*^{KO} Tub-G80^{ts}/UAS-*flippase* *Dronc*$^{KO\text{-}FRT\text{-}Dronc\text{-}GFP\text{-}APEX\text{-}FRT\text{-}QF}$. *Dronc*-expressing cells excising the rescue cassette are labelled with GFP (green); notice the reduction in number of Castor-expressing cells and size of the FCD (white arrows). Genotype: *Dronc−/−*: *109-30*-Gal4 (BL7023)/QUAS-*CD8-GFP* (BL 30002); *Dronc*^{KO} Tub-*G80*^{ts} (BL7019)/UAS-*flipasse* (BL8209) *Dronc*$^{KO\text{-}FRT\text{-}Dronc\text{-}GFP\text{-}APEX\text{-}FRT\text{-}QF}$.

F Wild-type expression of the follicular marker Castor (red and/or grey) in a representative germarium of the following genotype: CTRL: *109-30*-Gal4 (BL7023)/QUAS-*CD8-GFP* (BL 30002); *Dronc*^{KO} Tub-G80^{ts} (BL7019)/TM6b.

G Wild-type expression of the follicular marker Eyes absent (Eya) (red and/or grey) in a representative germarium of the following genotype: CTRL: *109-30*-Gal4 (BL7023)/QUAS-*CD8-GFP* (BL 30002); *Dronc*^{KO} Tub-G80^{ts} (BL7019)/TM6b.

H Eya expression (red, grey and white arrows) in follicular cells without Dronc. Notice the reduced expression of Eya (white arrows). Genotype: *Dronc−/−*: *109-30*-Gal4 (BL7023)/QUAS-*CD8-GFP* (BL 30002); *Dronc*^{KO} Tub-*G80*^{ts} (BL7019)/UAS-*flipasse* (BL8209) *Dronc*$^{KO\text{-}FRT\text{-}Dronc\text{-}GFP\text{-}APEX\text{-}FRT\text{-}QF}$.

I Schematic depicting the lineage tracing of *ptc-Gal4* expressing cells (coloured in red) in the germarium at 29°C.

J Representative 2D projection of confocal images showing a germarium expressing UAS-*Histone-RFP* (red) under the regulation of *ptc-Gal4* driver at 29°C. FasIII (green) and DAPI (blue) label the follicular cells and the nuclei of the germarium, respectively. Genotype: *ptc-Gal4* (BL2017)/+; UAS-*Histone-RFP* (BL56555) Tub-*G80*^{ts} (BL7019)/+.

K Castor expression (red, grey and white arrows) in a representative *Dronc* mutant germarium of the following genotype *ptc-Gal4* (BL2017)/QUAS-*CD8-GFP* (BL 30002); *Dronc*^{KO} Tub-*G80*^{ts} (BL7019)/UAS-*Flipasse* (BL8209) *Dronc*$^{KO\text{-}FRT\text{-}Dronc\text{-}GFP\text{-}APEX\text{-}FRT\text{-}QF}$. *Dronc*-expressing cells excising the rescue cassette are labelled with GFP (green); notice the reduction in number of Castor-expressing cells in the FCD as well as the size (white arrows).

L Quantification of somatic cells in S-phase labelled by EdU incorporation in control (CTRL: *ptc-Gal4* (BL2017)/+; Tub-*G80*^{ts} (BL7019)/+; $n = 30$) versus *Dronc* mutant germaria (*Dronc−/−*: *ptc-Gal4* (BL2017)/+; *Dronc*^{KO} Tub-*G80*^{ts} (BL7019)/UAS-*flippase* (BL8209) *Dronc*$^{KO\text{-}FRT\text{-}Dronc\text{-}GFP\text{-}APEX\text{-}FRT\text{-}QF}$; $n = 23$). Statistical significance was determined using an unpaired parametric Welch's *t*-test (****$P \leq 0.0001$).

Data information: Scale bars represent 10 μm in the entire figure. Experimental flies were kept after eclosion from the pupae for 14 days at 29°C prior to dissection. All the experimental data shown have been obtained from $N \geq 2$ biological replicates. The median and quartiles are indicated in the violin plots. All the quantifications were made in germaria containing one single group of germline cells wrapped by follicular cells.
Source data are available online for this figure.

 © 2023 The Authors

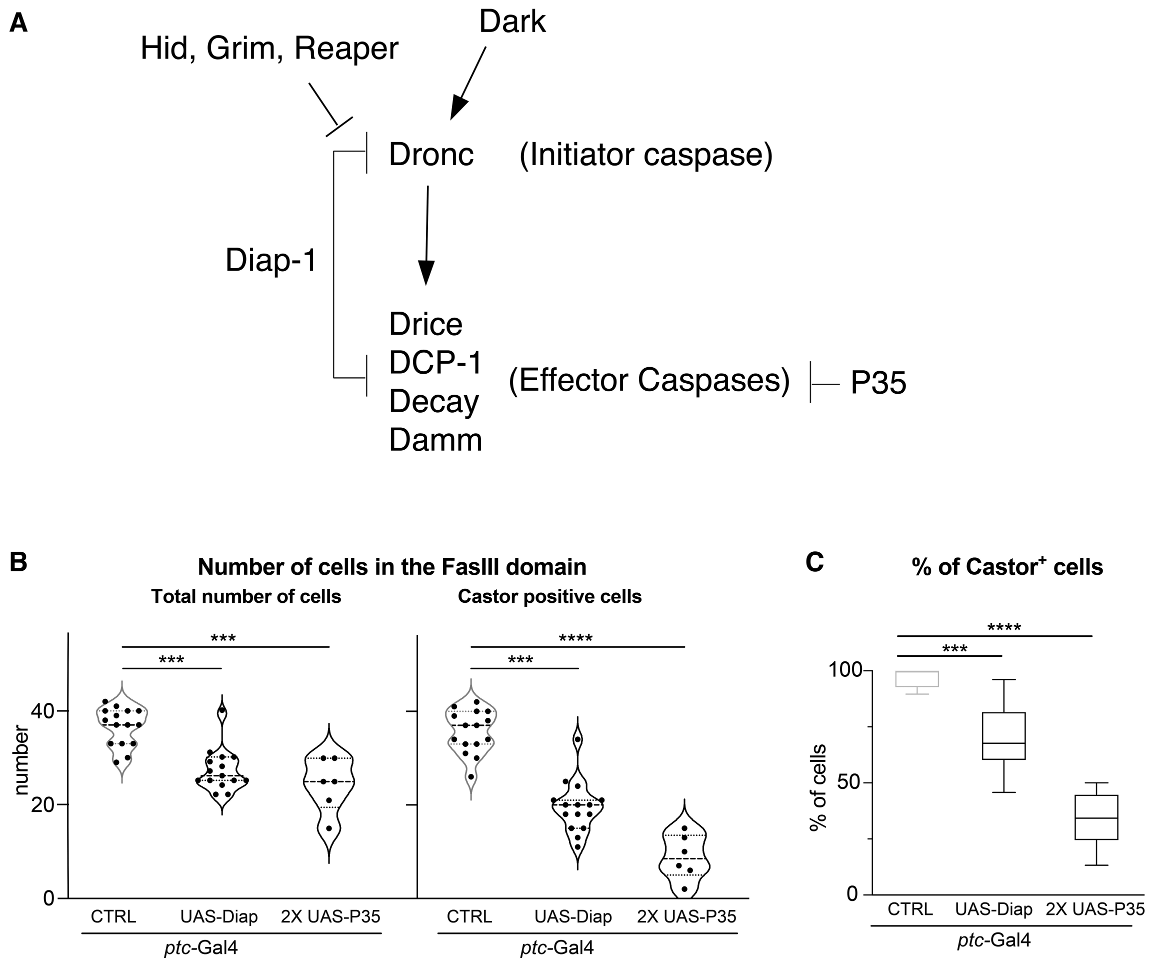
A
Hid, Grim, Reaper
Dark
Dronc
(Initiator caspase)
Diap-1
Drice
DCP-1
Decay
Damm
(Effector Caspases)
P35
B
Number of cells in the FasIII domain
Total number of cells
Castor positive cells
***
***
****
***
number
40
20
0
CTRL
UAS-Diap
2X UAS-P35
ptc-Gal4
C
% of Castor+ cells
****
***
100
50
0
% of cells
CTRL
UAS-Diap
2X UAS-P35
ptc-Gal4

Figure EV3.

Figure EV3. Caspase inhibition alters the cellular properties of ovarian somatic cells.

A Schematic depicting the caspase pathway, upstream regulators of *Dronc* and specific inhibitors of caspase activation such as Diap-1 and P35.

B Quantification of total number of follicular cells (left) or Castor-expressing cells (right) within the FasIII cellular domain in the following genotypes from left to right: *ptc-Gal4* (BL2017) ($N = 2$; $n = 15$). *ptc-Gal4* (BL2017)/UAS-*Diap1* (BL63819) ($N = 2$; $n = 15$) *ptc-Gal4* (BL2017)/UAS-P35 (BL5072); UAS-P35 (BL5073)/+ ($N = 1$; $n = 6$). Statistical significance was established by using Kruskal–Wallis test and Dunn's multiple comparison post-test (***$P \leq 0.001$; ****$P \leq 0.0001$).

C Percentage of Castor-expressing cells versus the total number of Follicular cells (FasIII$^+$ cells) in germaria of the genotypes indicated in (B). *n* numbers are shown in (B). A Kruskal–Wallis test and Dunn's multiple comparison post-test were used to determine statistical significance (***$P \leq 0.001$; ****$P \leq 0.0001$).

Data information: Experimental flies were kept after eclosion from the pupae for 14 days at 29°C prior to dissection. The violin plots show the mean and standard deviations in the entire figure. The box plot shows the median, first quartile and third quartile of the dataset. The whiskers illustrate the range between the maximum and minimum values of the dataset. All the quantifications were made in germaria containing one single group of germline cells wrapped by follicular cells.
Source data are available online for this figure.

 © 2023 The Authors

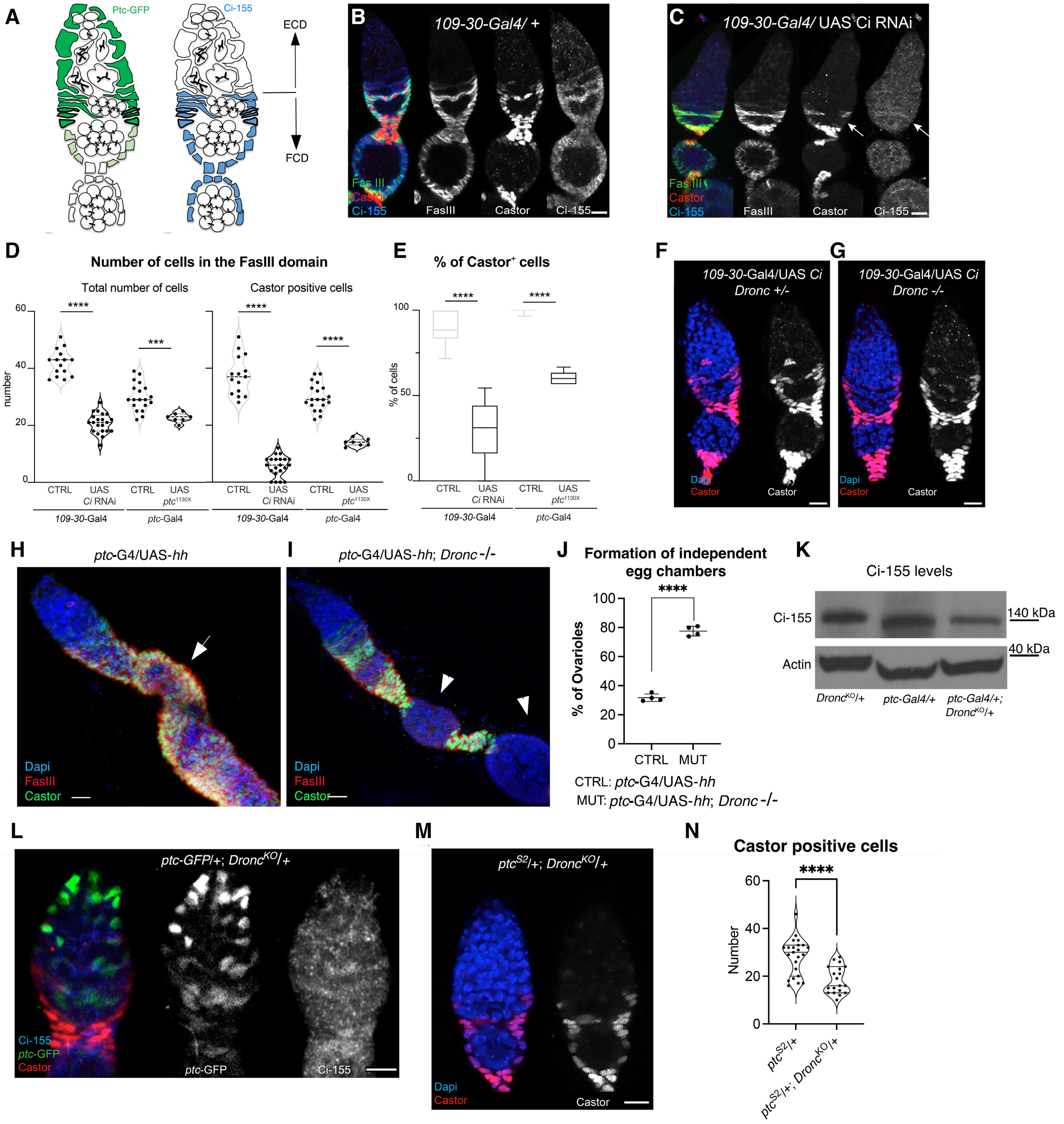
A
Ptc-GFP
Ci-155
ECD
FCD
B
109-30-Gal4/ +
Fas III
Castor
Ci-155
FasIII
Castor
Ci-155
C
109-30-Gal4/ UAS Ci RNAi
Fas III
Castor
Ci-155
FasIII
Castor
Ci-155
D
Number of cells in the FasIII domain
Total number of cells
Castor positive cells
number
CTRL
UAS Ci RNAi
CTRL
UAS ptc1130X
109-30-Gal4
ptc-Gal4
E
% of Castor+ cells
% of cells
F
109-30-Gal4/UAS Ci Dronc +/-
Dapi
Castor
Castor
G
109-30-Gal4/UAS Ci Dronc -/-
Dapi
Castor
Castor
H
ptc-G4/UAS-hh
Dapi
FasIII
Castor
I
ptc-G4/UAS-hh; Dronc -/-
Dapi
FasIII
Castor
J
Formation of independent egg chambers
% of Ovarioles
CTRL
MUT
CTRL: ptc-G4/UAS-hh
MUT: ptc-G4/UAS-hh; Dronc -/-
K
Ci-155 levels
Ci-155
Actin
140 kDa
40 kDa
DroncKO/+
ptc-Gal4/+
ptc-Gal4/+; DroncKO/+
L
ptc-GFP/+; DroncKO/+
Ci-155
ptc-GFP
Castor
ptc-GFP
Ci-155
M
ptcS2/+; DroncKO/+
Dapi
Castor
Castor
N
Castor positive cells
Number
ptcS2/+
ptcS2/+; DroncKO/+

Figure EV4.

Figure EV4. *Dronc* deficiency limits Hh-signalling.

A Schematics depicting the pattern of expression of ptc-GFP (green, left) and Ci-155 (blue, right) in the germarium. Notice the high levels of Ptc-GFP in Escort cells (ECs) and Follicular Stem Cells (FSCs) and the low levels in some follicular cells. High levels of Ci-155 are observed in follicular cells and the escort cells adjacent to the FSCs.

B Representative 2D projection of confocal images showing FasIII (green and/or grey), Castor (red and/or grey) and Ci-155 (blue and/or grey) expression in a control germarium (*109-30*-Gal4 (BL7023)/+).

C Representative 2D projection of confocal images showing FasIII (green and/or grey), Castor (red and/or grey) and Ci-155 (blue and/or grey) expression in a representative control germarium (*109-30*-Gal4 (BL7023)/+; UAS-*Ci*-RNAi (BL28984)/+). Notice the gaps in Castor expression within the Follicular region (white arrows).

D Quantification of total number of follicular cells (left) or Castor-expressing cells (right) within the FasIII cellular domain in the following genotypes from left to right: CTRL = *109-30*-Gal4 (BL7023)/+ (n = 15). *Dronc*−/− = *109-30*-Gal4 (BL7023)/+; UAS-*Ci*-RNAi (BL28984)/+ (n = 21). CTRL = *ptc-Gal4* (BL2017)/+; Tub-$G80^{ts}$ (BL7019)/+ (n = 19). UAS-ptc^{1130X}YFP = *ptc-Gal4* (BL2017)/UAS-ptc^{1130X}YFP (BL52215); Tub-$G80^{ts}$ (BL7019)/+ (n = 8). An unpaired parametric Welch's t-test was used to determine statistical significance (***$P \leq 0.001$; ****$P \leq 0.0001$).

E Percentage of Castor-expressing cells versus the total number of Follicular cells ($FasIII^{+}$ cells) in germaria of the genotypes indicated in (D). n numbers are shown in (D). A nonparametric Mann–Whitney t-tests were used to determine statistical significance (****$P \leq 0.0001$).

F, G Representative 2D projection of confocal images showing Castor expression (red and/or grey) and DAPI (blue) in mutant germarium of the following genotypes: *109-30*-Gal4 (BL7023)/UAS-*Ci* (BL32571); $Dronc^{KO}$ Tub-$G80^{ts}$ (BL7019)/+ (F). *109–30*Gal4 (BL7023)/UAS-*Ci* (BL32571); $Dronc^{KO}$ Tub-$G80^{ts}$ (BL7019)/UAS-*Flipasse* (BL8209) $Dronc^{KO\text{-}FRT\text{-}Dronc\text{-}GFP\text{-}}APEX^{\text{-}FRT\text{-}QF}$ (G).

H, I Representative 3D projections of confocal images showing Castor expression (green), FasIII (red) and DAPI (blue) in mutant germarium of the following genotypes: *ptc-Gal4* (BL2017)/UAS-*hh-EGFP.H* (BL81024); Tub-$G80^{ts}$ (BL7019)/+ (H). *ptc-Gal4* (BL2017)/UAS-*hh-EGFP.H* (BL81024); $Dronc^{KO}$ Tub-$G80^{ts}$ (BL7019)/UAS-*Flipasse* (BL8209) $Dronc^{KO\text{-}FRT\text{-}Dronc\text{-}GFP\text{-}APEX\text{-}FRT\text{-}suntag\text{-}HA\text{-}Cherry}$ (I). Whereas Castor expression is continuous and the egg chambers are normally fused in the genetic background of (H), caspase deficiency in (I) facilitates the formation of independent egg chambers and discontinuity in Castor expression (arrowheads).

J Frequency of ovarioles showing independent egg chambers and castor discontinuity in the genotypes shown in (H and I). *ptc-Gal4* (BL2017)/UAS-*hh-EGFP.H* (BL81024); Tub-$G80^{ts}$ (BL7019)/+. (N = 4, n = 445). *ptc-Gal4* (BL2017)/UAS-*hh-EGFP.H* (BL81024); $Dronc^{KO}$ Tub-$G80^{ts}$ (BL7019)/UAS-*Flipasse* (BL8209) $Dronc^{KO\text{-}FRT\text{-}Dronc\text{-}GFP\text{-}APEX\text{-}FRT\text{-}suntag\text{-}HA\text{-}Cherry}$. ($N$ = 4, n = 335). An unpaired parametric Welch's t-test was used to determine statistical significance (****$P \leq 0.0001$).

K Western blot showing Ci-155 expression (upper lane) and actin (bottom lane, loading control) in the genotypes indicated in the picture. Notice the downregulation of Ci-155 72 h after siRNA treatment.

L Representative 2D projection of confocal images showing the expression of Ci-155 (blue and grey channels), *ptc*-GFP (green and grey) and Castor (red and grey) in *ptc*-$GFP^{CB02030}$ (a gift from Isabel Guerrero)/+; $Dronc^{KO}$Tub-$G80^{ts}$ (BL7019)/+ germaria. Notice the downregulation of *ptc*-GFP and Ci-155 (compare with Fig 4A).

M Representative 2D projection of confocal images showing the expression of Castor (blue and grey) and DAPI (blue) in a ptc^{S2}*(BL6332)*/+; $Dronc^{KO}$/+ germarium.

N Quantification of Castor expression within the FasIII domain in the following genotypes: ptc^{S2}*(BL6332)*/+ (N = 2; n = 23). ptc^{S2}*(BL6332)*/+; $Dronc^{KO}$/+ (N = 2; n = 19). An unpaired parametric Welch's t-test was used to determine statistical significance (****$P \leq 0.0001$).

Data information: Scale bars represent 10 μm in all of the confocal images of the figure. Experimental flies were kept after eclosion from the pupae for 14 days at 29°C prior to dissection. The median and quartiles are indicated in the violin plots. The box plots show the median, first quartile and third quartile of datasets. The whiskers illustrate the range between the maximum and minimum values of datasets. All the quantifications were made in germaria containing one single group of germline cells wrapped by follicular cells.

Source data are available online for this figure.

 © 2023 The Authors

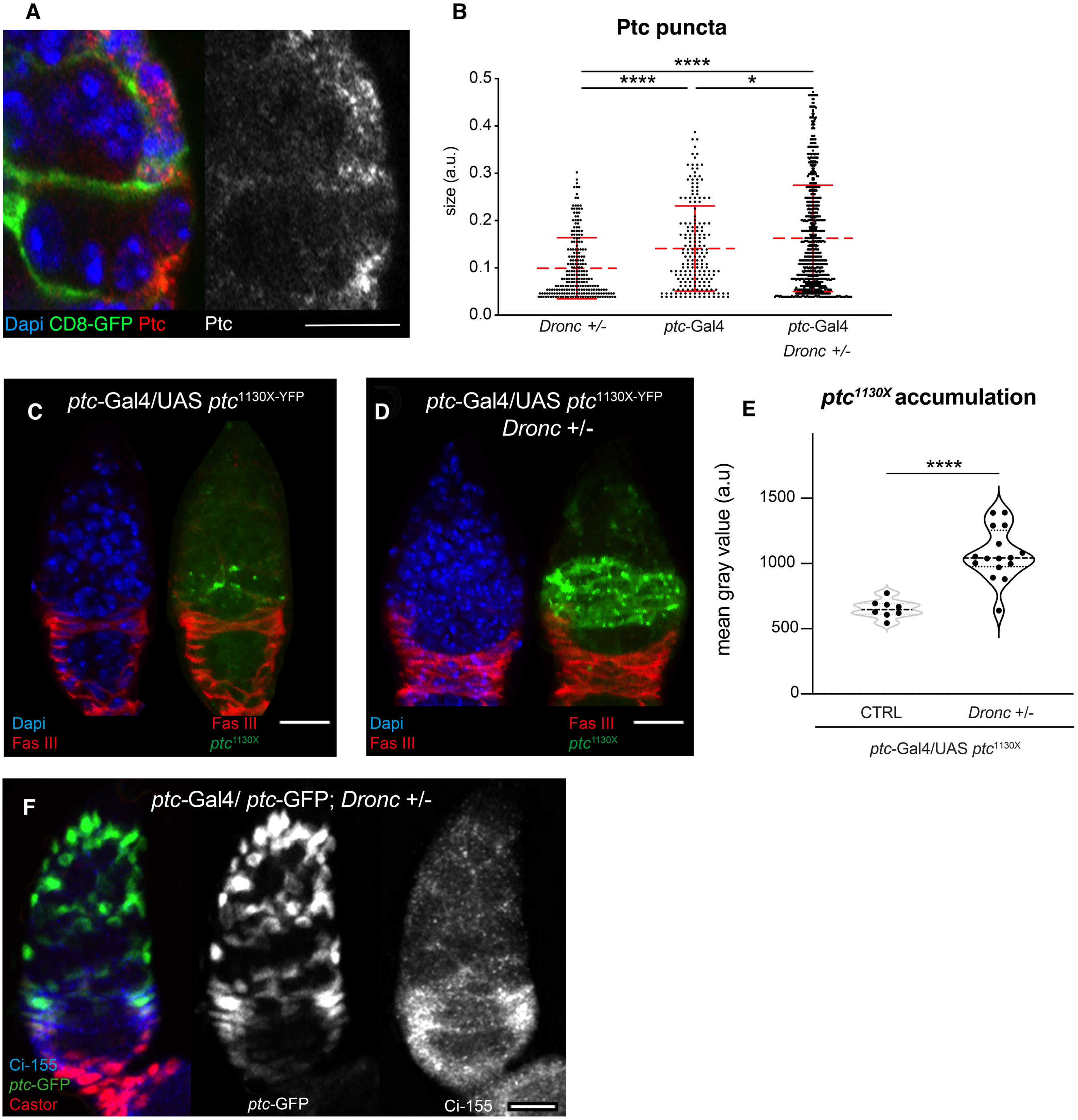
A
Dapi CD8-GFP Ptc
Ptc
B
Ptc puncta
size (a.u.)
0.0
0.1
0.2
0.3
0.4
0.5
****
****
*
Dronc +/-
ptc-Gal4
ptc-Gal4 Dronc +/-
C
ptc-Gal4/UAS ptc1130X-YFP
Dapi
Fas III
Fas III
ptc1130X
D
ptc-Gal4/UAS ptc1130X-YFP Dronc +/-
Dapi
Fas III
Fas III
ptc1130X
E
ptc1130X accumulation
mean gray value (a.u)
0
500
1000
1500
****
CTRL
Dronc +/-
ptc-Gal4/UAS ptc1130X
F
ptc-Gal4/ ptc-GFP; Dronc +/-
Ci-155
ptc-GFP
Castor
ptc-GFP
Ci-155

Figure EV5.

Figure EV5. ***Dronc* deficiency facilitates the intracellular accumulation of Ptc.**

A Representative 2D projection of confocal images showing the expression of Ptc (red and grey), mCD8-GFP (green) and DAPI (blue) in a *ptc-Gal4* (BL2017)/UAS-*mCD8-GFP (BL108068)*; *Dronc*^{KO} Tub-*G80*^{ts} (BL7019)/+ germarium. Notice the intracellular.

B Estimation of Ptc-positive puncta size in germaria of the following genotypes: *Dronc*^{KO}/+ (n = 10). *ptc-Gal4* (BL2017)/+ (n = 9). *ptc-Gal4* (BL2017)/+; *Dronc*^{KO} Tub-*G80*^{ts} (BL7019)/+ (n = 10). The statistical significance between groups was established using one-way ANOVA Tukey's multiple comparisons test (****$P \leq 0.0001$, ***$P \leq 0.001$, *$P \leq 0.05$). Notice the enlargement of Ptc-positive puncta in a double heterozygous germaria (*ptc-Gal4*/+; *Dronc*+/−). The mean and the standard deviation are indicated in the graph.

C, D Expression of the *ptc*^{1130X}YFP (green) in representative germaria of the following genotypes: *ptc-Gal4* (BL2017)/UAS-*ptc*^{1130X}YFP (BL52215); Tub-*G80*^{ts} (BL7019) (C) *ptc-Gal4* (BL2017)/UAS-*ptc*^{1130X}YFP (BL52215); *Dronc*^{KO} Tub-*G80*^{ts} (BL7019)/+ (D). Dapi (blue) and FasIII (red) stainings label the nuclei and follicular cells, respectively. Notice the preferential accumulation of GFP signal within the ECs next to the boundary of FasIII expression in (D).

E Quantification of *ptc*^{1130X}-YFP expression levels in germaria of the following genotypes: CTRL = *ptc-Gal4* (BL2017)/ UAS-*ptc*^{1130X} YFP (BL52215); Tub-*G80*^{ts} (BL7019) (n = 8). *Dronc*+/− = *ptc-Gal4* (BL2017)/UAS-*ptc*^{1130X}YFP (BL52215); *Dronc*^{KO} Tub-*G80*^{ts} (BL7019)/+ (n = 16). An unpaired parametric Welch's t-test was used to determine statistical significance (****$P \leq 0.0001$). Median and quartiles are shown in the violin plot.

F Representative confocal image showing the expression of Ci-155 (blue and/or grey), *ptc*-GFP (green and/or grey) and Castor (red) in germaria of the following genotype *ptc-Gal4* (BL2017)/*ptc*-GFP^{CB02030}; *Dronc*^{KO} Tub-*G80*^{ts} (BL7019)/+. Notice that the expression levels of Ci, *ptc*-GFP and Castor are largely restored.

Data information: Scale bars represent 10 μm in all of the confocal images of the figure. All the experimental data shown have been obtained from $N \geq 2$ biological replicates. Experimental flies were kept after eclosion from the pupae for 14 days at 29°C prior to dissection. All the quantifications were made in germaria containing one single group of germline cells wrapped by follicular cells.
Source data are available online for this figure.

 © 2023 The Authors

Table of contents

Appendix Figure S1. Figure showing that *Dronc* deficiency promotes the Ptc-dependent induction of autophagy that ultimately alters the cellular properties ovarian somatic cells. Pages 2-3.

Appendix Table S1. Description of fly lines and genotypes used in the experiments of Main Figures. Pages 4-7

Appendix Table S2. Description of fly lines and genotypes used in the experiments of Expanded View and Appendix Figures. Pages 8-9

Appendix Figure S2. Figure describing the counting methodology used for follicular cells throughout the manuscript. Pages 10-11

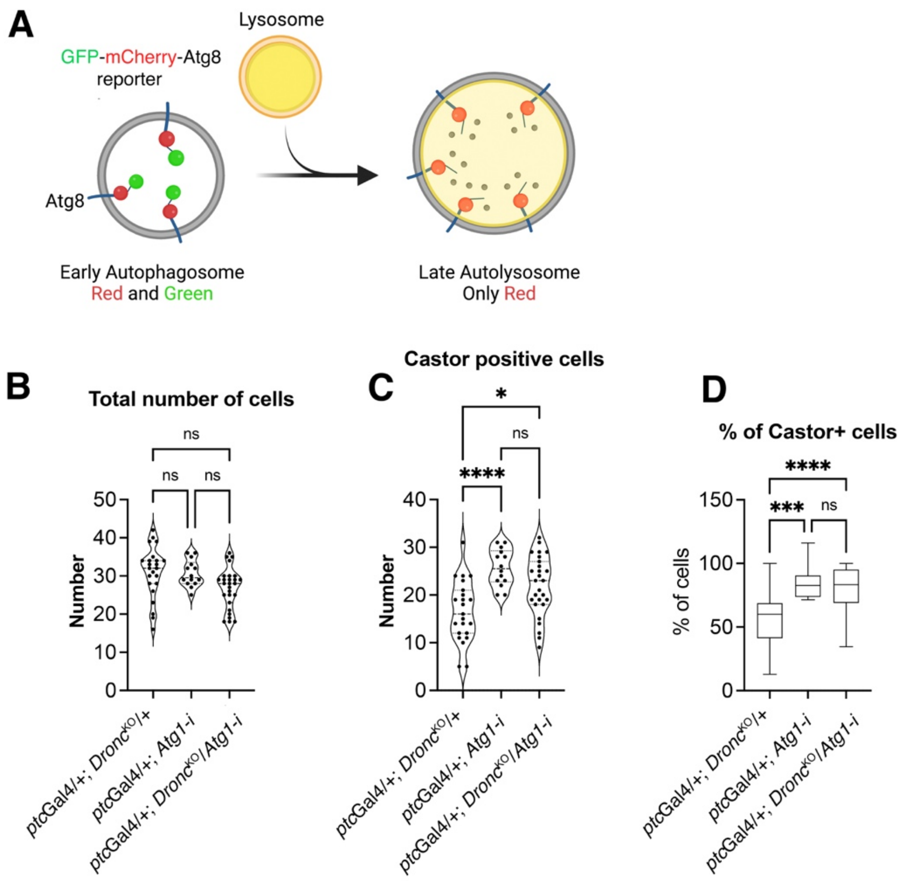

Appendix Figure S1.

A. Schematic depicting the labelling of early autophagosomes (green and red) and late autophagolysosomes (only red) using GFP-Cherry-Atg8. Diagram generated with BioRender.

B. Quantification of total number of follicular cells within the FasIII cellular domain in the following genotypes:

ptc-Gal4 (BL2017)/+; *Dronc*^{KO}Tub-*G80*^{ts} (BL7019)/+. (n=23)

ptc-Gal4 (BL2017)/+; Tub-*G80*^{ts} (BL7019)/UAS-*Atg1*-RNAi (BL35177). (n=14)

ptc-Gal4 (BL2017)/+; *Dronc*^{KO}Tub-*G80*^{ts} (BL7019)/UAS-*Atg1*-RNAi (BL35177). (n=27)

A Kruskal-Wallis test and Dunn's multiple comparison post-test were used to determine statistical significance (n.s.= not significant).

C. Quantification of the total number of Castor-expressing cells within the FasIII cellular domain in the genotypes described in B. n number is indicated also in B. An ordinary one-way ANOVA and Dunnett's multiple comparison post-test were used to determine statistical significance (n.s.= not significant; * $p \leq 0.05$; **** $p \leq 0.0001$).

D. Percentage of Castor-expressing cells versus the total number of Follicular cells ($FasIII^+$ cells) in germaria of the genotypes indicated in B. n numbers are shown in B. Data are expressed as box-and-whiskers plots, with min to max range as whiskers. A Kruskal-Wallis test and Dunn's multiple comparison post-test were used to determine statistical significance (n.s.= not significant; ***p≤0.001; ****p≤0.0001).

Data information. Experimental flies were kept after eclosion from the pupae for 14 days at 29°C prior to dissection. All the experimental data shown have been obtained from N ≥ 2 biological replicates. The median and quartiles are indicated in the violin plots. The box plot shows the median, first quartile, and third quartile of the dataset. The whiskers illustrate the range between the maximum and minimum values of the dataset. All the quantifications were made in germaria containing one single group of germline cells wrapped by follicular cells.

Genotypes and fly lines information	
**Figure**	**Genotype**
**1B**	Actin *DBS-S-QF*, UAS-*mCD8-GFP*, QUAS-*tomato-HA*/+; ;QUAS-*Gal4 /*+ (BL83123)
**1C**	Actin *DBS-S-QF*, UAS-*mCD8-GFP*, QUAS-*tomato-HA*/+; QUAS-*flippase* (BL30126)/+; Actin5C FRT-*stop*-FRT *lacZ*-nls/+ (BL6355)
**1D-F**	Actin *DBS-S-QF*, UAS-*mCD8-GFP*, QUAS-*tomato-HA*/+; QUAS-*flippase* (BL30126)/+; Actin5C FRT-*stop*-FRT *lacZ*-nls/+ (BL6355)
**1G**	**Control:**<br>Actin *DBS-S-QF*, UAS-*mCD8-GFP*, QUAS-*tomato-HA*/+; *ptc-Gal4* (BL2017)/+<br><br>**Experimental:**<br>Actin *DBS-S-QF*, UAS-*mCD8-GFP*, QUAS-*tomato-HA*/+; *ptc-Gal4* (BL2017)/UAS-*Dronc*RNAi (a gift from Pascal Meier)
**1I**	w;; *Dronc*$^{KO\text{-}Gal4}$ / UAS-*Histone-RFP* (BL56555)
**1J**	w;; *Dronc*^{TurboID} (a gift from Masayuki Miura)/ Tm3, Sb
**2A, 2B**	**Control:**<br>*109-30-Gal4* (BL7023)/+; *Dronc*^{KO} UAS-*Histone-RFP* (BL56555) Tub-*G80*^{ts} (BL7019)/+<br><br>**Experimental without Dronc expression:**<br>*109-30-Gal4* (BL7023)/+; *Dronc*^{KO} UAS-*Histone-RFP* (BL56555) Tub-*G80*^{ts} (BL7019)/ UAS-*flippase* (BL8209) *Dronc*$^{KO\text{-}FRT\text{-}Dronc\text{-}GFP\text{-}APEX\text{-}FRT\text{-}QF}$
**2C, 2D**	**Control:**<br>*109-30-Gal4* (BL7023)/+; *Dronc*^{KO} UAS-*Histone-RFP* (BL56555) Tub-*G80*^{ts} (BL7019)/+;<br>**Experimental without Dronc expression:**<br>*109-30-Gal4* (BL7023)/+; *Dronc*^{KO} UAS-*Histone-RFP* (BL56555) Tub-*G80*^{ts} (BL7019)/ UAS-*flippase* (BL8209) *Dronc*$^{KO\text{-}FRT\text{-}Dronc\text{-}GFP\text{-}APEX\text{-}FRT\text{-}QF}$.<br><br>**Control:**<br>*ptc-Gal4* (BL2017)/+; UAS-*Histone-RFP* (BL56555) Tub-*G80*^{ts} (BL7019)/+<br>**Experimental without Dronc expression:**<br>*ptc-Gal4* (BL2017)/+; *Dronc*^{KO} UAS-*Histone-RFP* (BL56555) Tub-*G80*^{ts} (BL7019)/ UAS-*flippase* (BL8209) *Dronc*$^{KO\text{-}FRT\text{-}Dronc\text{-}GFP\text{-}APEX\text{-}FRT\text{-}QF}$.
**2E**	**White triangle** = *ptc-Gal4* (BL2017)/+; +/+<br><br>**Yellow circle** = *ptc-Gal4* (BL2017)/+; *Dronc*^{KO} UAS-*Histone-RFP* (BL56555) Tub-*G80*^{ts} (BL7019)/ +<br><br>**Red square** = *ptc-Gal4* (BL2017)/+; *Dronc*^{KO} UAS-*Histone-RFP* (BL56555) Tub-*G80*^{ts} (BL7019)/ UAS-*flippase* (BL8209) *Dronc*$^{KO\text{-}FRT\text{-}Dronc\text{-}GFP\text{-}APEX\text{-}FRT\text{-}suntag\text{-}HA}$<br><br>**Blue circle** = *ptc-Gal4* (BL2017)/UAS-*Dronc* (BL56198); *Dronc*^{KO} UAS-*Histone-RFP* (BL56555) Tub-*G80*^{ts} (BL7019)/+
**2F**	*ptc-Gal4* (BL2017)/+; *Dronc*^{KO} UAS-*Histone-RFP* (BL56555) Tub-*G80*^{ts} (BL7019)/ +
**2G**	*ptc-Gal4* (BL2017)/+; *Dronc*^{KO} UAS-*Histone-RFP* (BL56555) Tub-*G80*^{ts} (BL7019)/UAS-*Dronc* (BL56198)

**2I-K**	**Control:**<br>*109-30-Gal4* (BL7023)/UAS-FUCCI(BL55100); *Dronc*^{KO} Tub-*G80*^{ts} (BL7019)/ +)<br><br>**Experimental without Dronc expression:**<br>*109-30-Gal4* (BL7023)/UAS-FUCCI(BL55100); *Dronc*^{KO} Tub-*G80*^{ts} (BL7019) / UAS-*flippase* (BL8209) *Dronc*$^{\text{KO-FRT-Dronc-GFP-APEX-FRT-suntagHA}}$)
**3A-C**	**Control:**<br>*109-30-Gal4* (BL7023)/+<br><br>**Experimental without Dronc expression:**<br>*109-30-Gal4* (BL7023)/+; *Dronc*^{KO} Tub-*G80*^{ts} (BL7019) / UAS-*flippase* (BL8209) *Dronc*$^{\text{KO-FRT Dronc-GFP-Apex FRT-Suntag-HA-Cherry}}$<br><br>**Experimental expressing a catalytically inactive form of Dronc:**<br>*109-30-Gal4* (BL7023)/+; *Dronc*^{KO} Tub-*G80*^{ts} (BL7019) / UAS-*flippase* (BL8209) *Dronc*$^{\text{KO-FRT Dronc-GFP-Apex FRT-Dronc FL-CAEA-Suntag-HA-Cherry}}$
**3D-F**	**Control:**<br>*109-30-Gal4*/+; Tub-*G80*^{ts} (BL7019)/+<br><br>**Experimental:**<br>*109-30-Gal4* (BL7023)/UAS-*Drice*RNAi UAS-*Decay*RNAi (a gift from Pascal Meier); UAS-*Damm*RNAi, UAS-*Dcp1*RNAi (a gift from Pascal Meier)<br><br>*109-30-Gal4* (BL7023)/+; Tub-*G80*^{ts} (BL7019)/ UAS-*Dcp1*RNAi (BL28909)<br><br>*109-30-Gal4* (BL7023)/+; Tub-*G80*^{ts} (BL7019)/ UAS-*Drice*RNAi (BL32403)<br><br>*109-30-Gal4* (BL7023)/UAS-*Dark-sh*; Tub-*G80*^{ts} (BL7019)/+ (a gift from M. Miura)<br><br>*109-30-Gal4* (BL7023)/UAS-microRNA-RHG; Tub-*G80*^{ts} (BL7019)/+ (a gift from Istwar Hariharan)
**4A-C**	**Control:**<br>*109-30-Gal4* (BL7023)*/ptc*-GFP$^{\text{CB02030}}$ (a gift from Isabel Guerrero)<br><br>**Experimental:**<br>*109-30-Gal4* (BL7023)*/ptc*-GFP$^{\text{CB02030}}$*;* *Dronc*^{KO}Tub-*G80*^{ts} (BL7019)/ UAS-*flippase* *Dronc*$^{\text{KO-FRT-Dronc-GFP-APEX-FRT-QF}}$
**4F-G**	**Control:**<br>*109-30-Gal4* (BL7023)/UAS-*smo*^{Act} (BL44621); *Dronc*^{KO}Tub-*G80*^{ts} (BL7019)/+<br><br>**Experimental:**<br>*109-30-Gal4* (BL7023)/UAS-*smo*^{Act} (BL44621); *Dronc*^{KO}Tub-*G80*^{ts} (BL7019)/ UAS-*flippase* (BL8209) *Dronc*$^{\text{KO-FRT-Dronc-GFP-APEX-FRT-QF}}$

**4H, 4I**	*Dronc -/- = CTRL= 109-30-Gal4* (BL7023)/; *Dronc*^{KO}Tub-*G80*^{ts} (BL7019)/ UAS-*flippase* (BL8209) *Dronc*$^{KO\text{-}FRT\text{-}Dronc\text{-}GFP\text{-}APEX\text{-}FRT\text{-}QF}$<br><br>*CTRL= 109-30-Gal4* (BL7023)/UAS-*smo*^{Act} (BL44621); *Dronc*^{KO}Tub-*G80*^{ts} (BL7019)/+<br><br>*Dronc -/- = 109-30-Gal4* (BL7023)/UAS-*smo*^{Act} (BL44621); *Dronc*^{KO}Tub-*G80*^{ts} (BL7019)/ UAS-*flippase* (BL8209) *Dronc*$^{KO\text{-}FRT\text{-}Dronc\text{-}GFP\text{-}APEX\text{-}FRT\text{-}QF}$<br><br>*CTRL= 109-30-Gal4* (BL7023)/UAS-*Ci* (BL28984); *Dronc*^{KO}Tub-*G80*^{ts} (BL7019)/+<br><br>*Dronc -/- = 109-30-Gal4* (BL7023)/UAS- UAS-*Ci* (BL28984); *Dronc*^{KO}Tub-*G80*^{ts} (BL7019)/ UAS-*flippase* (BL8209) *Dronc*$^{KO\text{-}FRT\text{-}Dronc\text{-}GFP\text{-}APEX\text{-}FRT\text{-}QF}$
**4J, 4K**	*Dronc +/+ = ptc-Gal4* (BL2017)/+; Tub-*G80*^{ts} (BL7019)<br><br>*Dronc +/- = ptc-Gal4* (BL2017)/+; *Dronc*^{KO}Tub-*G80*^{ts} (BL7019)/+<br><br>*Dronc +/- UAS-Dronc = ptc-Gal4* (BL2017)/+; *Dronc*^{KO}Tub-*G80*^{ts} (BL7019)/UAS-*Dronc* (BL56198)
**5A-C**	*Dronc*^{KO}Tub-*G80*^{ts} (BL7019)/+<br><br>*ptc-Gal4* (BL2017)/+; Tub-*G80*^{ts} (BL7019)/+<br><br>*ptc-Gal4* (BL2017)/+; *Dronc*^{KO}Tub-*G80*^{ts} (BL7019)/+
**5D-G**	**Control:**<br>*ptc-Gal4* (BL2017)/UAS-*GFP-mCherry-Atg8* (BL37749)<br><br>**Experimental:**<br>*ptc-Gal4* (BL2017)/UAS-*GFP-mCherry-Atg8* (BL37749); *Dronc*^{KO}Tub-*G80*^{ts} (BL7019)/+
**5H-I**	*CTRL = 109-30*-Gal4 (BL7023)/+; *Dronc*^{KO}Tub-*G80*^{ts} (BL7019)/+<br><br>*Dronc -/- = 109-30Gal4* (BL7023)/+; *Dronc*^{KO}Tub-*G80*^{ts} (BL7019)/ UAS-*flippase* (BL8209) *Dronc*$^{KO\text{-}FRT\text{-}Dronc\text{-}GFP\text{-}APEX\text{-}FRT\text{-}QF}$<br><br>*Dronc -/-* UAS-*ptc*-RNAi = *109-30Gal4* (BL7023)/UAS-*ptc*-RNAi (BL55686); *Dronc*^{KO}Tub-*G80*^{ts} (BL7019)/ UAS-*flippase* (BL8209) *Dronc*$^{KO\text{-}FRT\text{-}Dronc\text{-}GFP\text{-}APEX\text{-}FRT\text{-}QF}$
**6A-D**	**Control:**<br>*ptc-Gal4* (BL2017)/ UAS-*GFP-mCherry-Atg8* (BL37749)<br><br>**Experimental:**<br>*ptc-Gal4* (BL2017)/ UAS-*GFP-mCherry-Atg8* (BL37749); *Dronc*^{KO}Tub-*G80*^{ts} (BL7019)/+

**6E-G**	*Dronc*^{KO}Tub-*G80*^{ts} (BL7019)/+<br><br>*ptc-Gal4* (BL2017)/+; Tub-*G80*^{ts} (BL7019)/+<br><br>*ptc-Gal4* (BL2017)/+; *Dronc*^{KO}Tub-*G80*^{ts} (BL7019)/+
**6J-L**	*ptc-Gal4* /+ = *ptc-Gal4* (BL2017)/+; Tub-*G80*^{ts} (BL7019)/+<br><br>*ptc-Gal4* /*Atg8*i = *ptc-Gal4* (BL2017)/UAS-*Atg8*RNAi (VDRC 109654); Tub-*G80*^{ts} (BL7019)/+<br><br>*ptc-Gal4* /*Atg8*i *Dronc*^{KO}/*Dronc*$^{KO\text{-}suntag\text{-}HA\text{-}Cherry}$= *ptc-Gal4* (BL2017)/UAS-*Atg8*RNAi (VDRC 109654); *Dronc*^{KO}Tub-*G80*^{ts} (BL7019)/ *Dronc*$^{KO\text{-}FRT\text{-}Dronc\text{-}APEX\text{-}GFP\text{-}FRT\text{-}suntag\text{-}HA\text{-}Cherry}$

Appendix Table S1. Description of fly lines and genotypes used in the experiments of the main Figures.

Genotypes and fly lines information	
**EV Figure**	**Genotype**
**EV 1B**	Actin *DBS-S-QF*, UAS-*mCD8-GFP*, QUAS-*tomato-HA*/+; QUAS-*flippase* (BL30126)/+; Actin5C FRT-*stop*-FRT *lacZ*-nls/+ (BL6355)
**EV 1C**	w;; *Dronc*$^{KO\text{-}Gal4}$ / UAS-*Histone-RFP* (BL56555)
**EV 1D**	*Dronc::*V5::TurboID/+ (a gift from Masayuki Miura)
**EV 1E-F**	*yw hs-flippase*$^{1.22}$/+; FRT80 *Dronc*^{I29}/ FRT80 Ubi*GFP*
**EV 2D**	*109-30*-Gal4 (BL7023)/+; UAS-*Histone-RFP* (BL56555) Tub-*G80*^{ts} (BL7019)/+
**EV 2E**	*109-30*-Gal4 (BL7023)/QUAS-*CD8-GFP* (BL 30002); *Dronc*^{KO} Tub-*G80*^{ts} (BL7019) / UAS-*flipasse* (BL8209) *Dronc*$^{KO\text{-}FRT\text{-}Dronc\text{-}GFP\text{-}APEX\text{-}FRT\text{-}QF}$
**EV 2F, 2G**	*109-30*-Gal4 (BL7023)/QUAS-*CD8-GFP* (BL 30002); *Dronc*^{KO} Tub-G80^{ts} (BL7019) / TM6b
**EV 2H**	*109-30*-Gal4 (BL7023)/QUAS-*CD8-GFP* (BL 30002); *Dronc*^{KO} Tub-*G80*^{ts} (BL7019) / UAS-*flipasse* (BL8209) *Dronc*$^{KO\text{-}FRT\text{-}Dronc\text{-}GFP\text{-}APEX\text{-}FRT\text{-}QF}$
**EV 2J**	*ptc-Gal4* (BL2017)/+; UAS-*Histone-RFP* (BL56555) Tub-*G80*^{ts} (BL7019)/+
**EV 2K**	*ptc-Gal4* (BL2017)/QUAS-*CD8-GFP* (BL 30002); *Dronc*^{KO} Tub-*G80*^{ts} (BL7019) / UAS-*Flipasse* (BL8209) *Dronc*$^{KO\text{-}FRT\text{-}Dronc\text{-}GFP\text{-}APEX\text{-}FRT\text{-}QF}$
**EV 2L**	**Control:**<br>*ptc-Gal4* (BL2017)/+; Tub-*G80*^{ts} (BL7019)/+<br><br>**Experimental:**<br>*ptc-Gal4* (BL2017)/+; *Dronc*^{KO} Tub-*G80*^{ts} (BL7019) / UAS-*flippase* (BL8209) *Dronc*$^{KO\text{-}FRT\text{-}Dronc\text{-}GFP\text{-}APEX\text{-}FRT\text{-}QF}$
**EV 3B, 3C**	*ptc-Gal4* (BL2017)<br>*ptc-Gal4* (BL2017)/UAS-*Diap1* (BL63819)<br>*ptc-Gal4* (BL2017)/UAS-P35 (BL5072); UAS-P35 (BL5073)/+
**EV 4B**	*109-30*-Gal4 (BL7023)/+
**EV 4C**	*109-30*-Gal4 (BL7023)/+; UAS-*Ci*-RNAi (BL28984)/+
**EV 4D, 4E**	CTRL= *109-30*-Gal4 (BL7023)/+<br><br>*Dronc -/-* =*109-30*-Gal4 (BL7023)/+; UAS-*Ci*-RNAi (BL28984)/+<br><br>CTRL = *ptc-Gal4* (BL2017)/ + ; Tub-*G80*^{ts} (BL7019)/+<br><br>UAS-*ptc*^{1130X}YFP = *ptc-Gal4* (BL2017)/ UAS-*ptc*^{1130X}YFP (BL52215); Tub-*G80*^{ts} (BL7019)/+
**EV 4F**	*109-30*-Gal4 (BL7023)/UAS-*Ci* (BL32571); *Dronc*^{KO} Tub-*G80*^{ts} (BL7019) / +
**EV 4G**	*109-30*Gal4 (BL7023)/UAS-*Ci* (BL32571); *Dronc*^{KO} Tub-*G80*^{ts} (BL7019)/ UAS-*Flipasse* (BL8209) *Dronc*$^{KO\text{-}FRT\text{-}Dronc\text{-}GFP\text{-}APEX\text{-}FRT\text{-}QF}$
**EV 4H**	*ptc-Gal4* (BL2017)/UAS-*hh-EGFP.H* (BL81024); Tub-*G80*^{ts} (BL7019) / +

**EV 4I**	*ptc-Gal4* (BL2017)/ UAS-*hh-EGFP.H* (BL81024); *Dronc*^{KO} Tub-*G80*^{ts} (BL7019)/ UAS-*Flipasse* (BL8209) *Dronc*$^{KO-FRT-Dronc-GFP-APEX-FRT-suntag-HA-Cherry}$
**EV 4J**	**Control:**<br>*ptc-Gal4* (BL2017)/UAS-*hh-EGFP.H* (BL81024); Tub-*G80*^{ts} (BL7019) / +<br><br>**Experimental:**<br>*ptc-Gal4* (BL2017)/ UAS-*hh-EGFP.H* (BL81024); *Dronc*^{KO} Tub-*G80*^{ts} (BL7019)/ UAS-*Flipasse* (BL8209) *Dronc*$^{KO-FRT-Dronc-GFP-APEX-FRT-suntag-HA-Cherry}$.
**EV 4L**	*ptc*-GFP^{CB02030} (a gift from Isabel Guerrero) /+; *Dronc*^{KO}Tub-*G80*^{ts} (BL7019)/+
**EV 4M**	*ptc*^{S2}*(BL6332)/+; Dronc*^{KO}/+
**EV 4N**	**Control:**<br>*ptc*^{S2}*(BL6332)/+*<br>**Experimental:**<br>*ptc*^{S2}*(BL6332)/+; Dronc*^{KO}/+
**EV 5A**	*ptc-Gal4* (BL2017)/UAS-*mCD8-GFP (BL108068)*; *Dronc*^{KO} Tub-*G80*^{ts} (BL7019)/+
**EV 5B**	*Dronc*^{KO}/+<br>*ptc-Gal4* (BL2017)/+<br>*ptc-Gal4* (BL2017)/+; *Dronc*^{KO} Tub-*G80*^{ts} (BL7019) /+
**EV 5C**	*ptc-Gal4* (BL2017)/ UAS-*ptc*^{1130X}YFP (BL52215); Tub-*G80*^{ts} (BL7019)
**EV 5D**	*ptc-Gal4* (BL2017)/ UAS-*ptc*^{1130X}YFP (BL52215); *Dronc*^{KO} Tub-*G80*^{ts} (BL7019) /+
**EV 5E**	**Control:**<br>CTRL = *ptc-Gal4* (BL2017)/ UAS-*ptc*^{1130X} YFP (BL52215); Tub-*G80*^{ts} (BL7019). (n=8)<br>**Experimental:**<br>*Dronc +/- = ptc-Gal4* (BL2017)/ UAS-*ptc*^{1130X}YFP (BL52215); *Dronc*^{KO} Tub-*G80*^{ts} (BL7019) /+
**EV 5F**	*ptc-Gal4* (BL2017)/ *ptc*-GFP^{CB02030}; *Dronc*^{KO} Tub-*G80*^{ts} (BL7019) / +
**Appendix Fig S4B-D**	*ptc-Gal4* (BL2017)/+; *Dronc*^{KO}Tub-*G80*^{ts} (BL7019)/+<br>*ptc-Gal4* (BL2017)/+; Tub-*G80*^{ts} (BL7019)/UAS-*Atg1*-RNAi (BL35177)<br>*ptc-Gal4* (BL2017)/+; *Dronc*^{KO}Tub-*G80*^{ts} (BL7019)/UAS-*Atg1*-RNAi (BL35177)

Appendix Table S2. Description of fly lines and genotypes used in the experiments of Expanded View and Appendix Figures.

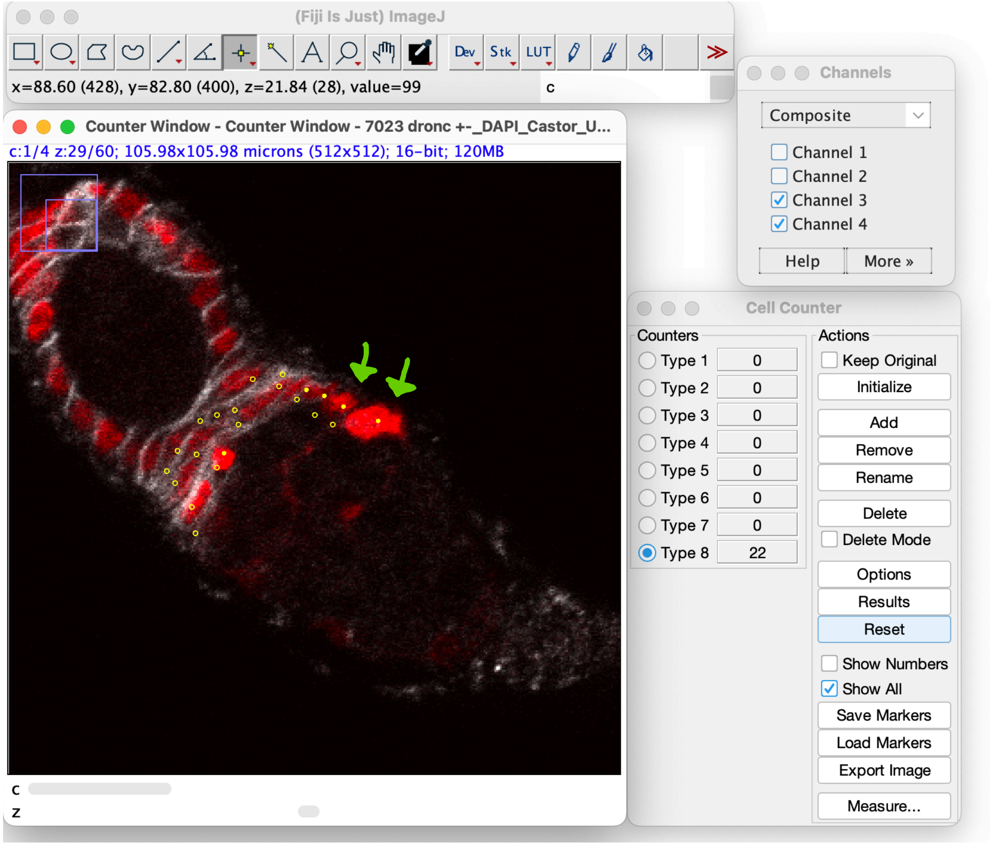

Focal plane 29

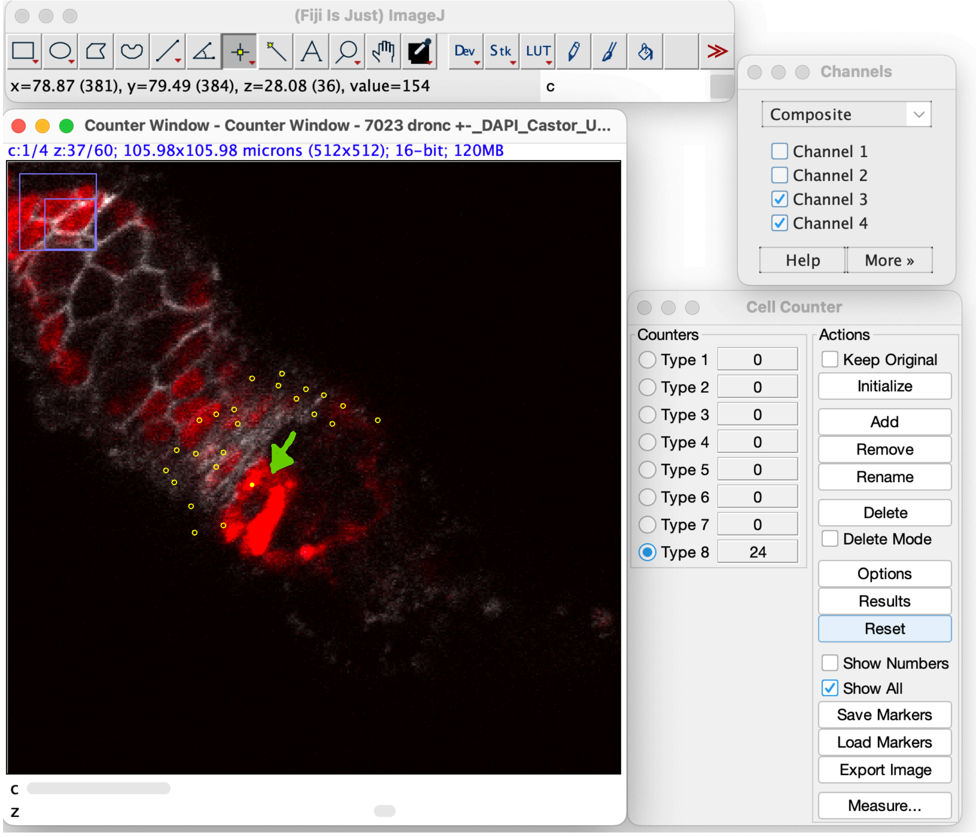

Focal plane 37

Appendix Figure S2. Figure describing the counting methodology used for follicular cells throughout the manuscript. To permanently label cells expressing the nuclear marker Histone-RFP (this is specific of the sample shown but we also used other markers to recognise follicular cells), we employ the Cell Counter plug-in in the Fiji software. Specifically, cells identified in a given focal plane, such as focal plane 29, are manually marked with a solid dot (some examples are indicated by the green arrowhead) while the counter simultaneously displays the number of dots generated. This process is repeated for each confocal plane in the Z-stack. Notably, in subsequent focal planes, such as focal plane 37, the position of previously labelled cells is displayed with an empty circle while new cells are marked with solid dots. This approach ensures accurate cell counting of follicular cells throughout the entire Z-stack.
