# Supplementary material for: Non‐apoptotic caspase activation ensures the homeostasis of ovarian somatic stem cells
Source: EMBO Rep. 2023 Apr 11;24(6):e51716. doi: 10.15252/embr.202051716 (PMC10240206; doi:10.15252/embr.202051716)
Supplement: Supplementary file 11 [file EMBR-24-e51716-s006.pdf]

EMBO reports

Non-apoptotic caspase activation ensures the homeostasis of ovarian somatic stem cells

Alessia Galasso, Derek Xu, Claire Hill, Daria Iakovleva, Irina Stefana, and Luis Alberto Baena Lopez
DOI: 10.15252/embr.202051716

Corresponding author(s): Luis Alberto Baena Lopez (alberto.baenalopez@path.ox.ac.uk)

Review Timeline:

Submission Date:	28th Sep 20
Editorial Decision:	4th Nov 20
Revision Received:	20th Dec 22
Editorial Decision:	16th Feb 23
Revision Received:	22nd Feb 23
Accepted:	14th Mar 23

Editor: Martina Rembold

Transaction Report:

(Note: With the exception of the correction of typographical or spelling errors that could be a source of ambiguity, letters and reports are not edited. Depending on transfer agreements, referee reports obtained elsewhere may or may not be included in this compilation. Referee reports are anonymous unless the Referee chooses to sign their reports.)

1st Revision - Editorial Decision 4th Nov 2020

Dear Alberto,

Thank you for the submission of your research manuscript to our journal. We have now received the full set of referee reports that is copied below.

As you will see, all referees acknowledge that the findings are potentially interesting but they also have a number of - largely overlapping - suggestions how the data could be strengthened that need to be addressed. I think the most important points have been nicely summarized by referee 3: it will be essential to clarify the genetic relationship between Patched and Dronc and the relationship between Ptc and autophagy. Another important point relates to the fact that the current description of events is limited to mild stress conditions. Two of the referees point out that it will be important to define whether 29 degree Celcius induce indeed only mild stress and to analyse what happens at standard temperature. Referee 4 is an expert in Drosophila ovary development and raises several concerns regarding the analysis of follicular phenotypes that need to be resolved. Referee 1 suggests testing which of the effector caspases are required in the ovary. I agree that this is an interesting question that will add value to the paper but it is not mandatory form our side to experimentally test this and can be included if straightforward to test. Finally, the writing and the description of the experiments should be revised, also to make the study more accessible to scientists not familiar with Drosophila genetics.

Given these constructive comments, we would like to invite you to revise your manuscript with the understanding that the referee concerns (as detailed above and in their reports) must be fully addressed and their suggestions taken on board. Please address all referee concerns in a complete point-by-point response. Acceptance of the manuscript will depend on a positive outcome of a second round of review. It is EMBO reports policy to allow a single round of revision only and acceptance or rejection of the manuscript will therefore depend on the completeness of your responses included in the next, final version of the manuscript.

We invite you to submit your manuscript within three months of a request for revision. This would be February 4th, 2021 in your case. However, we are aware of the fact that many laboratories are not fully functional due to COVID-19 related shutdowns and we have therefore extended the revision time for all research manuscripts under our scooping protection to allow for the extra time required to address essential experimental issues. Please contact us to discuss the time needed and the revisions further.

IMPORTANT NOTE: we perform an initial quality control of all revised manuscripts before re-review. Your manuscript will FAIL this control and the handling will be DELAYED if the following APPLIES:

1) A data availability section is missing.
2) Your manuscript contains error bars based on n=2. Please use scatter blots showing the individual datapoints in these cases. The use of statistical tests needs to be justified.

When submitting your revised manuscript, please carefully review the instructions that follow below. Failure to include requested items will delay the evaluation of your revision.

When submitting your revised manuscript, we will require:

1) a .docx formatted version of the manuscript text (including legends for main figures, EV figures and tables). Please make sure that the changes are highlighted to be clearly visible.

2) individual production quality figure files as .eps, .tif, .jpg (one file per figure).
Please download our Figure Preparation Guidelines (figure preparation pdf) from our Author Guidelines pages https://www.embopress.org/page/journal/14693178/authorguide for more info on how to prepare your figures.

3) a .docx formatted letter INCLUDING the reviewers' reports and your detailed point-by-point responses to their comments. As part of the EMBO Press transparent editorial process, the point-by-point response is part of the Review Process File (RPF), which will be published alongside your paper.

4) a complete author checklist, which you can download from our author guidelines (). Please insert information in the checklist that is also reflected in the manuscript. The completed author checklist will also be part of the RPF.

5) Please note that all corresponding authors are required to supply an ORCID ID for their name upon submission of a revised manuscript (). Please find instructions on how to link your ORCID ID to your account in our manuscript tracking system in our Author guidelines
()

6) We replaced Supplementary Information with Expanded View (EV) Figures and Tables that are collapsible/expandable online.

A maximum of 5 EV Figures can be typeset. EV Figures should be cited as 'Figure EV1, Figure EV2" etc... in the text and their respective legends should be included in the main text after the legends of regular figures.

- For the figures that you do NOT wish to display as Expanded View figures, they should be bundled together with their legends in a single PDF file called *Appendix*, which should start with a short Table of Content. Appendix figures should be referred to in the main text as: "Appendix Figure S1, Appendix Figure S2" etc. See detailed instructions regarding expanded view here:

- Additional Tables/Datasets should be labeled and referred to as Table EV1, Dataset EV1, etc. Legends have to be provided in a separate tab in case of .xls files. Alternatively, the legend can be supplied as a separate text file (README) and zipped together with the Table/Dataset file.

7) Please note that a Data Availability section at the end of Materials and Methods is now mandatory. In case you have no data that requires deposition in a public database, please state so instead of refereeing to the database.
See also < https://www.embopress.org/page/journal/14693178/authorguide#dataavailability>). Please note that the Data Availability Section is restricted to new primary data that are part of this study.

8) We would also encourage you to include the source data for figure panels that show essential data. Numerical data should be provided as individual .xls or .csv files (including a tab describing the data). For blots or microscopy, uncropped images should be submitted (using a zip archive if multiple images need to be supplied for one panel). Additional information on source data and instruction on how to label the files are available .

9) Our journal encourages inclusion of *data citations in the reference list* to directly cite datasets that were re-used and obtained from public databases. Data citations in the article text are distinct from normal bibliographical citations and should directly link to the database records from which the data can be accessed. In the main text, data citations are formatted as follows: "Data ref: Smith et al, 2001" or "Data ref: NCBI Sequence Read Archive PRJNA342805, 2017". In the Reference list, data citations must be labeled with "[DATASET]". A data reference must provide the database name, accession number/identifiers and a resolvable link to the landing page from which the data can be accessed at the end of the reference. Further instructions are available at .

10) Regarding data quantification
The following points must be specified in each figure legend:
- the name of the statistical test used to generate error bars and P values,
- the number (n) of independent experiments (please specify technical or biological replicates) underlying each data point,
- the nature of the bars and error bars (s.d., s.e.m.)
Discussion of statistical methodology can be reported in the materials and methods section, but figure legends should contain a basic description of n, P and the test applied.
- Please also include scale bars in all microscopy images.

11) As part of the EMBO publication's Transparent Editorial Process, EMBO reports publishes online a Review Process File to accompany accepted manuscripts. This File will be published in conjunction with your paper and will include the referee reports, your point-by-point response and all pertinent correspondence relating to the manuscript.

You are able to opt out of this by letting the editorial office know (emboreports@embo.org). If you do opt out, the Review Process File link will point to the following statement: "No Review Process File is available with this article, as the authors have chosen not to make the review process public in this case."

We would also welcome the submission of cover suggestions, or motifs to be used by our Graphics Illustrator in designing a cover.

I look forward to seeing a revised version of your manuscript when it is ready. Please let me know if you have questions or comments regarding the revision.

Kind regards,
Martina

Martina Rembold, PhD
Senior Editor
EMBO reports

****

Referee #1:

The paper by Baena-Lopez and colleagues describes a pro-survival function of the apoptotic caspases in the drosophila ovary. Whereas the molecular mechanisms underlying caspase activation and functions during apoptosis have been thoroughly worked out, much less is known about the regulation, function, and the extent of caspase-dependent non-lethal cellular processes (also called CDPs). Drosophila has proven to be the major model organism in which CDPs are discovered and characterized. The Baena-Lopez group has recently generated multiple elegant genetic tools designed to discover and characterize new CDPs in Drosophila. Using this toolkit, they now provide evidence that under moderate stress, the Drosophila ovarian follicular stem cells activate the caspase pathway in a nonapoptotic manner. This activation is required for the fine-tuning of Hh signaling and autophagy through the regulation of the Hh receptor, Patched. They further show that caspase activation sustains the proliferation and differentiation of the ovarian somatic cells under these conditions. Finally, they present preliminary data indicating some degree of conservation of these findings in ovarian somatic cells from humans.
Overall, the authors present several intriguing and original observations which expand our understanding of the role of caspases in CDPs. Nevertheless, while I find this paper highly interesting and informative, the flow of reading and the logic behind the design of the experiments was not always trivial for me to understand. In particular that the paper includes a new set of complicated genetic reporters and mutant drivers. Furthermore, the experiments demonstrating genetic interactions between dronc and the hh signaling are also confusion. Better explanations of the expected outcomes and the conclusions from the actual results would be of great help. In general, the Results section shall be edited such that it would be clear to read and understand for non-Drosophila professionados. A schematic model summarizing the main conclusions would also help grasping the main take home messages.
The initial discovery in this paper leans on a reporter of Dronc activity. However, the results demonstrate that it is the activity of the effector caspases that is required (i.e. that Dronc non-apoptotic function in this system is to activate the effector caspases, which in turn perform the non-apoptotic functions of proliferation and differentiation in the ovary). This shall be better emphasized throughout the paper, as the non-apoptotic functions of caspases in Drosophila can be divided to Dronc-dependent, effector caspase-independent functions (e.g. AiP, border cell migration), and effector-caspase dependent functions (e.g. spermatid individualization, ICM, epithelial thorax closure), to which the functions in the ovary belong.
Furthermore, among the effector caspases, the apoptotic ones, Drice and Dcp-1, are divided to major and minor, respectively, based on their 'killing' efficiency in apoptosis. The authors' experiments do not differentiate between the caspases, and which caspase is required in the ovary. Specific mutant and RNAi lines are available and could provide highly informative insights into the roles of the specific effector caspases (Drice? Dcp-1? Both? Neither one of them but instead the non-apoptotic caspases Damm or Decay?). The value of these experiments goes beyond the identification of a single protein, as in case Drice turns out to be the functional caspase in this system, it would suggest special tight regulation since it is a potent killer enzyme, whereas Dcp-1 or the other caspases might require more moderate regulation.
Minor points:
Fig. 2E-G, Expend in the text about how the FUCCI assay works.
Add explanations about castor in text, as it already appears in fig 1. Also, in some of the figure legends, castor is described as "Maker" instead of Marker.

Referee #2:

In this manuscript, the authors investigate a potentially novel non-apoptotic function of the initiator caspase Dronc under moderate stress in ovarian somatic stem cells in adult Drosophila females. First, they report non-apoptotic caspase activation in proliferative stem cell precursors under moderate stress. Because the caspase reporter was designed to detect Dronc activity, the authors examined the expression and a functional requirement of Dronc in these cells. Dronc is expressed in these cells and using a number of elegant mutant constructs of Dronc, the authors showed that Dronc is required for proliferation and differentiation of the follicular region in the germarium under moderate stress. This requirement appears to be dependent on Dronc catalytic activity and also on other components of the apoptotic pathway such as the effector caspases and to some extent on the upstream reaper,hid,grim genes. From here, the authors move on to identify the signaling pathway which is controlled by caspases and focus on the Hh pathway, because the down-regulation of Ci generated a phenotype very similar to the Dronc mutant phenotype. Loss of Dronc reduced the expression of Ci and the Hh receptor Ptc. Consistently, overexpression of Ci or Smo can rescue the Dronc mutant phenotype. However, from here on, things get a little bit murky when they started to look at a genetic interaction between Dronc and ptc. Ptc is a negative regulator of Hh signaling, but the genetic interaction with Dronc reveals the same phenotype as Dronc or Hh pathway mutants. Nevertheless, it appears that Dronc regulates the stability of Ptc because in Dronc mutants, the protein levels of Ptc increased. Finally, the authors also examined another pathway regulated by Ptc, namely autophagy. They used Ref2P as a marker for reduced autophagy and found increased Ref2P levels under ptc-Gal4/+ conditions. These increased Ref2P levels were reduced back to normal in a double heterozygous ptc-Gal4/+;Dronc/+ background which suggests that the differentiation defects caused by Dronc deficiency are linked to Ptc-induced autophagy. The authors also show that human Caspase-9 might have a similar regulatory role on Hh signaling and autophagy in human cells.

This is a potentially interesting paper, but a few problems need to be addressed. First, I found the writing very difficult to follow. Experiments are not well explained, figures are hard to figure out. Often, the authors mention markers such as Castor and don't explain what they label (or much later in the paper). What is the FasIII expression domain? The authors need to explain better.

After all, they want to address a wide audience, not just ovarian specialists.

Specific points:
1. The authors make the point that what they observe in this paper, occurs under moderate stress. By moderate stress, I assume they mean 29C, but that was never specifically mentioned. More important, what do they see under stress-free conditions? Is there caspase activation? Are there any phenotypes? It is a big omission to not even mention this.

2. The authors spent a lot of effort in Figure 1E-G to look at the expression of Dronc. However, the obtained expression pattern look somewhat different, especially 1E and 1F. What do they see with an antibody? Also, related to point #1, are these pattern stress induced?

3. The Fly-Fucci data in Fig 2 are not very well explained. As the authors mentioned, there are more cells in S phase, but are these data statistically significant? There seems to be a big discrepancy between the S-phase data in 2G and 2H. Also, the authors mention cells in G0 in the text, but the figure doesn't show any cells in G0. Do they mean G1?

4. In Fig 3, the authors found that other components of the apoptosis pathway such as effector caspases have a similar phenotype when inactivated as does Dronc and they conclude that "...these experiments confirmed the non-apoptotic and pro-survival role of caspase activation..." (line 230). I disagree with the term "confirmed" here. Maybe the authors can use "more evidence" in this context, but confirmed is a little far fetched.

5. I like the data in Fig 4F-I (the rescue of the Dronc-/- phenotype by expression of SmoACT or Ci). Did the authors attempt the opposite experiment, rescuing the smo-/- or ci-/- phenotype by Dronc overexpression?

6. The ptc data in Fig 4J,k are a little unclear and are inconsistent with the authors model. Maybe that is because the authors used double heterozygous ptc/Dronc conditions which I think can be a little tricky for epistasis experiments. What actually is the homozygous ptc-/- phenotype in these cells? Maybe that helps to resolve the counterintuitive result of the double heterozygous ptc/dronc interaction.

7. Fig 6: In addition to Ref2P, did the authors test other autophagy markers such as Atg8-GFP or some autophagic flux markers? That would strengthen the results about the role of caspases for autophagy.

8. Do Dronc mutants have an autophagic phenotype in this tissue?

9. I am a little confused by the result in EV Fig 2K. I am surprised that there are TUNEL-positive cells in Dronc-/- germaria. Aren't Dronc mutants completely lacking apoptosis? Can you actually show these data?

Referee #3:

In this manuscript the authors examine a non-apoptotic role for caspases in survival and differentiation of somatic tissues in stressed Drosophila ovaries. This is an important process that is potentially conserved between flies and humans. The researchers use state of the art approaches, and the data are in general of high quality. The results are interpreted quite aggressively so there are some concerns about particular conclusions. In addition, this is a very dense paper that while accessible to Drosophila researchers may not be to a general audience.

The authors use a clever reporter system to identify cells in which the Drosophila caspase Drice is active, was recently active, or active in the past. They show that in animals that have been stressed at 29C, Drice is activated in ovarian escort cells, but that not all of these cells undergo apoptosis and can give rise to pre-follicular cells. Using another clever construct, they go on to show that Dronc, an activator caspase of Drice, is expressed in escort and pre-follicular cells of heat stressed ovaries. Elimination of Dronc from follicular stem cells in heat stressed ovaries leads to a significant decrease in the number of pre-follicular cells and the number of these cells expressing the differentiation marker Castor. While the cell cycle distribution was not dramatically altered in Dronc mutants, there was a significant increase in the number of EdU positive cells. They also show that the enzymatic activity of Dronc was necessary for proliferation and differentiation.

Using a combination of UAS-RNAi constructs, the authors disrupted the initiators of programmed cell death Hid, Grim and Reaper. This had a significant effect on survival but not differentiation. The authors argue that this shows that the regulation of these processes is distinct, but it could just reflect one process being less sensitive to knock down of Hid, Grim and Reaper.

As previous work had demonstrated a role for Hedgehog signaling in the survival and differentiation of the somatic tissues of the ovary, the authors examined the relationship between caspase activation in stressed ovaries and the Hh pathway. They show that expression of activated Smo or over expression of Ci could rescue both the survival and differentiation defects in Dronc mutant cells. The also show a decrease in cells expressing the differentiation marker Castor in animals heterozygous for ptc-GAL4 and Dronc[-]. The authors point out both that ptc-GAL4 is a hypomorphic mutation of ptc and that the sign of this genetic

interaction doesn't make sense. Ptc is a negative regulator of the pathway; given the results with activated Smo and Ci, one would expect that lower Ptc levels would improve Dronc heterozygotes. With this surprising relationship, it is important to demonstrate that the interaction with Dronc is actually due to the GAL4 insertion in ptc. The GAL4 insertion in ptc is a P-element vector and therefore it can be excised using transposase. The authors should excise the P-element and determine whether the genetic interaction with Dronc is eliminated. Given that P-element activation can initiate recombination even in males, this excision should be done over a balancer.

In characterizing the ptc-GAL4 Dronc[-] heterozygotes the authors noted elevated Ptc protein puncta. The accumulation of Ptc was important not only for the Castor differentiation phenotype but also the survival phenotype as RNAi to ptc rescued both of these defects in Dronc mutant cells. The accumulation of Ptc puncta could correlate with the ability of Ptc to induce autophagy. In ptc-GAL4 Dronc heterozygotes Ref2P puncta (which accumulate when autophagy is blocked) decrease suggesting increased autophagy in this genetic combination. That increased autophagy was required for the decreased expression of the differentiation marker Castor, was tested by blocking autophagy in the ptc-GAL4 Dronc heterozygous background using RNAi to Atg1. In this combination expression of Castor was restored.

The authors make the point that autophagy is involved in differentiation and not survival, but they only do the experiments in the ptc-GAL4 Dronc heterozygous background. It would also be worthwhile to include knockdown Atg1 in Dronc-/- cells (which show a defect in cell survival) to demonstrate that Atg1 doesn't have a cell survival effect. When doing these experiments, it would be good to look at Ptc protein accumulation in Dronc-/- cells vs. Dronc-/- cells with RNAi to Atg1. These experiments might sort out the relationship between Ptc accumulation and induction of autophagy.

This is potentially a very interesting paper. The two major concerns are resolving the genetic relationship between ptc-GAL4 and Dronc- and the relationship between Ptc and autophagy.

Referee #4:

In this study, the authors investigate the non-apoptotic roles of caspases in the follicle epithelium in the Drosophila ovary. They find that, in flies maintained at 29C which they consider a moderate stress, caspases are activated but the cells do not undergo apoptosis. Loss of the caspase, dronc, causes a decrease in proliferation relative to wildtype cells and loss of expression of cas, which promotes differentiation in this lineage. These observations contribute to the emerging understanding that caspases have functions other than promoting apoptosis. The authors then provide evidence that dronc exerts these effects through an interaction with the hedgehog pathway. Specifically, dronc promotes hedgehog signaling by preventing the accumulation of ptc. The authors also show that some of the effects they see in the fly ovary are conserved in a human ovarian somatic cell line. Taken together, the observations in this study are provocative, and I think would be of value to the community. I found the images to be convincing for the most part and appreciated the careful quantification of the phenotypes throughout. However, there are several issues that should be addressed before publication.

1. The study is framed as an investigation of the role that caspases play in proliferation and differentiation during moderate stress, but it is not clear that 29C is a moderate stress, and evidence is not provided to show that these effects are specific to the 29C condition. The authors should test whether the activation of the caspase reporter, the requirements for dronc in proliferation and cas expression, and the effects on hedgehog signaling are also observed at a standard temperature (e.g. 25C). In addition, they should provide other evidence (or describe published data) indicating that 29C is, in fact, a stressful condition.

2. The effect of dronc knockout on the number of cells in the FasIII domain expressing cas (e.g. Fig. 2C and 3C) is not well described. FasIII expression extends well beyond the germarium, but the images provided and the number of cells counted in controls suggest that the analysis was limited to the germarium. The authors should provide more information about how the posterior boundary of the region to be analyzed was determined. In Fig. 2b, the lower number of follicle cells may be a secondary effect caused by a defect in budding from the germarium, in which case considering only the FasIII follicle cells in the germarium is somewhat arbitrary.

3. On a related note, it is not clear what effect the loss of cas expression has on the follicle epithelium . Are there defects in specification of the polar, stalk, and/or main body cell fates? Is eya expression affected? What is the significance of this effect for the tissue function?

4. What is the rationale for switching from 190-30-Gal4 in Fig. 3A-B to ptc-Gal4 in Fig. 3C-D? These drivers do not have the same expression pattern in the germarium and, in subsequent figures, the authors show that heterozygosity for ptc-Gal4 causes phenotypes. Thus, it is difficult to compare the results with 109-30-Gal4 vs. ptc-Gal4, as the authors attempt to do on p. 6.

5. The cas phenotype in ptc/+, dronc/+ (EV Fig. 4D) is not very convincing. The pattern of expression looks similar to wildtype. This phenotype should be quantified in the same way that it was in other parts of the paper (e.g. Fig. 2 and 3).

6. The labels for FSCs in Fig. 1G are not correct. FSCs are located to the anterior of Region 2b, but the lines are indicating positions that are within Region 2b, well downstream of the anterior face of the Region 2b cyst.

7. The ptc-Gal4, dronc +/- germarium in Fig. 5A seems much bigger than the other germaria in the other two images. Are there more escort cells in this genotype? The number of ptc puncta should be normalized to EC number or evidence should be provided that EC number is not significantly different between these genotypes.

Authors’ general response to the points raised by reviewers:
We first want to thank the reviewers for acknowledging the value and novelty of our results, as well as for their constructive and positive criticisms. They have helped us to considerably strengthen our manuscript. Despite the experimental difficulties we have faced during some phases of the pandemic, the reviewers can find below our detailed responses to their concerns and descriptions of the new experimental data (in black font). Some of our answers also include relevant extracts from the manuscript within quotation marks. The logic flow of the manuscript has been improved by including explanatory diagrams, a simplified text, and new explanations of the experimental rationale. We hope all of these changes will make the manuscript more accessible for a broader audience, including non-specialists in the field. Of course, we also hope that our enthusiasm regarding the improvements made in the manuscript will be shared by the reviewers.

Referee #1:

The paper by Baena-Lopez and colleagues describes a pro-survival function of the apoptotic caspases in the drosophila ovary. Whereas the molecular mechanisms underlying caspase activation and functions during apoptosis have been thoroughly worked out, much less is known about the regulation, function, and the extent of caspase-dependent non-lethal cellular processes (also called CDPs). Drosophila has proven to be the major model organism in which CDPs are discovered and characterized. The Baena-Lopez group has recently generated multiple elegant genetic tools designed to discover and characterize new CDPs in Drosophila. Using this toolkit, they now provide evidence that under moderate stress, the Drosophila ovarian follicular stem cells activate the caspase pathway in a nonapoptotic manner. This activation is required for the fine-tuning of Hh signaling and autophagy through the regulation of the Hh receptor, Patched. They further show that caspase activation sustains the proliferation and differentiation of the ovarian somatic cells under these conditions. Finally, they present preliminary data indicating some degree of conservation of these findings in ovarian somatic cells from humans.
Overall, the authors present several intriguing and original observations which expand our understanding of the role of caspases in CDPs. Nevertheless, while I find this paper highly interesting and informative, the flow of reading and the logic behind the design of the experiments was not always trivial for me to understand. In particular that the paper includes a new set of complicated genetic reporters and mutant drivers. Furthermore, the experiments demonstrating genetic interactions between dronc and the hh signaling are also confusion. Better explanations of the expected outcomes and the conclusions from the actual results would be of great help. In general, the Results section shall be edited such that it would be clear to read and understand for non-Drosophila professionados. A schematic model summarizing the main conclusions would also help grasping the main take home messages.

The logic flow of the manuscript has been improved by including explanatory diagrams, a simplified version of the text, and new explanations of the experimental rationale. We hope all of these changes will make the manuscript more accessible for a broader audience, including non-specialists in the field.

The initial discovery in this paper leans on a reporter of Dronc activity. However, the results demonstrate that it is the activity of the effector caspases that is required (i.e. that Dronc non-apoptotic function in this system is to activate the effector caspases, which in turn perform the non-apoptotic functions of proliferation and differentiation in the ovary). This shall be better emphasized throughout the paper, as the non-apoptotic functions of caspases in Drosophila can be divided to Dronc-dependent, effector caspase-independent functions (e.g. AiP, border cell migration), and effector-caspase dependent functions (e.g. spermatid individualization, ICM, epithelial thorax closure), to which the functions in the ovary belong.

The point raised by the reviewer has been emphasised in the results and discussion section of the revised version of the manuscript.

Furthermore, among the effector caspases, the apoptotic ones, Drice and Dcp-1, are divided to major and minor, respectively, based on their 'killing' efficiency in apoptosis. The authors' experiments do not differentiate between the caspases, and which caspase is required in the ovary. Specific mutant and RNAi lines are available and could provide highly informative insights into the roles of the specific effector caspases (Drice? Dcp-1? Both? Neither one of them but instead the non-apoptotic caspases Damm or Decay?). The value of these experiments goes beyond the identification of a single protein, as in case Drice turns out to be the functional caspase in this system, it would suggest special tight regulation since it is a potent killer enzyme, whereas Dcp-1 or the other caspases might require more moderate regulation.

We appreciate this suggestion of the referee since our new experimental data has been useful to determine the combinatorial activity of effector caspases is required in or biological context to sustain the homeostasis of ovarian somatic cells (new Figure 3D-F).
Beyond the requested experiment by the reviewer, we have explored the upstream regulatory mechanisms of Dronc in our cellular scenario reaching the conclusion that to some extent relies on Dark and pro-apoptotic factors (new figure 3D-F). Together these findings suggest that the entire caspase pathway can be engaged for non-lethal purposes in ovarian somatic cells under moderate thermal stress.

"Next, we evaluated whether the implementation of Dronc functions could be mediated by the effector caspases; main Dronc substrates during apoptosis. To that end, we overexpressed in the follicular stem cells validated RNAi constructs able to simultaneously reduce their expression (Drice, DCP-1, Decay and Damm) (Leulier et al., 2006) (EV Fig 3A). This genetic manipulation caused proliferation and differentiation phenotypes reminiscent of Dronc deficiency (Figs 3D-F). Since effector caspases can be functionally redundant (Xu et al., 2006), we separately assessed the contribution of DCP-1 and Drice to these phenotypes. These experiments also caused proliferation defects but milder differentiation phenotypes (Figs 3D-F; notice the proportion of Castor-expressing cells versus the total number of follicular cells was reduced to a lesser extent), thus suggesting a likely implementation of Dronc functions mediated by the combinatorial activity of effector caspases. Supporting this hypothesis, the overexpression of either Diap-1 or P35 (a viral pan-effector caspase inhibitor (Hay et al., 1994)) also limited the proliferation and differentiation of ovarian somatic cells (EV Figs 3A-C). In these experiments, we used ptc-Gal4 to consistently reduce the activity of effector caspases in the entire ECD and the follicular stem cells. In a different set of experiments, we evaluated the potential mechanisms of Dronc activation by ectopically expressing either a short-hairpin RNA against Dark (Drosophila orthologue of Apaf-1) (Obata et al., 2014) or a microRNA against the main pro-apoptotic factors (Hid, Reaper and Grim; RHG) (Siegrist et al., 2010), respectively (EV Fig 3A). Intriguingly, these experiments reduced the total number of follicular and Castor-expressing cells (Fig 3D, 3E); however, the proportion of Castor-expressing cells versus the total was not affected (Fig 3F). These findings indicated that differentiation and proliferation phenotypes can be uncoupled. More importantly, our data provided evidence that the entire caspase pathway can be activated for non-apoptotic purposes in somatic cells of the ovary."

Minor points:
Fig. 2E-G, Expend in the text about how the FUCCI assay works.

This information has been included in the new version of the text (see new Figure 2H). We have also repeated the entire Fucci analysis using a UAS-Fucci strain exclusively expressed in somatic cells (new figure 2I-K). This has helped us to improve the resolution of our quantifications by avoiding the Fucci signal from the germline cells. The new data suggest that Dronc deficiency delays the progression of cell cycle during S-phase (new figure 2I-K).

Add explanations about castor in text, as it already appears in fig 1. Also, in some of the figure legends, castor is described as "Maker" instead of Marker.

This has been corrected.

Referee #2:

In this manuscript, the authors investigate a potentially novel non-apoptotic function of the initiator caspase Dronc under moderate stress in ovarian somatic stem cells in adult Drosophila females. First, they report non-apoptotic caspase activation in proliferative stem cell precursors under moderate stress. Because the caspase reporter was designed to detect Dronc activity, the authors examined the expression and a functional requirement of Dronc in these cells. Dronc is expressed in these cells and using a number of elegant mutant constructs of Dronc, the authors showed that Dronc is required for proliferation and differentiation of the follicular region in the germarium under moderate stress. This requirement appears to be dependent on Dronc catalytic activity and also on other components of the apoptotic pathway such as the effector caspases and to some extent on the upstream reaper,hid,grim genes. From here, the authors move on to identify the signaling pathway which is controlled by caspases and focus on the Hh pathway, because the down-regulation of Ci generated a phenotype very similar to the Dronc mutant phenotype. Loss of Dronc reduced the expression of Ci and the Hh receptor Ptc. Consistently, overexpression of Ci or Smo can rescue the Dronc mutant phenotype. However, from here on, things get a little bit murky when they started to look at a genetic interaction between Dronc and ptc. Ptc is a negative regulator of Hh signaling, but the genetic interaction with Dronc reveals the same phenotype as Dronc or Hh pathway mutants. Nevertheless, it appears that Dronc regulates the stability of Ptc because in Dronc mutants, the protein levels of Ptc increased. Finally, the authors also examined another pathway regulated by Ptc, namely autophagy. They used Ref2P as a marker for reduced autophagy and found increased Ref2P levels under ptc-Gal4/+ conditions. These increased Ref2P levels were reduced back to normal in a double heterozygous ptc-Gal4/+;Dronc/+ background which suggests that the differentiation defects caused by Dronc deficiency are linked to Ptc-induced autophagy. The authors also show that human Caspase-9 might have a similar regulatory role on Hh signaling and autophagy in human cells.

This is a potentially interesting paper, but a few problems need to be addressed. First, I found the writing very difficult to follow. Experiments are not well explained, figures are hard to figure out. Often, the authors mention markers such as Castor and don't explain what they label (or much later in the paper). What is the FasIII expression domain? The authors need to explain better. After all, they want to address a wide audience, not just ovarian specialists.

The logic flow of the manuscript has been improved by including explanatory diagrams, a simplified version of the text, and new explanations of the rationale underlying all of the experiments. We hope all of these changes will make the manuscript more accessible for a

broader audience, including non-specialists in the field.

Specific points:
1. The authors make the point that what they observe in this paper, occurs under moderate stress. By moderate stress, I assume they mean 29C, but that was never specifically mentioned. More important, what do they see under stress-free conditions? Is there caspase activation? Are there any phenotypes? It is a big omission to not even mention this.

We thank the reviewer for raising this point since it has helped us to better understand the dynamics of caspase activation in our system. The new version of the manuscript describes in detail the caspase activation at 25°C and at 29°C in different types of somatic cells. These experiments suggest that non-apoptotic caspase activation is potently boosted under moderate thermal stress (29°C) (new Figure 1D-F). We have also confirmed that such caspase activation relies on Dronc since deficiency of this factor prevents the labelling of somatic cells with our caspase sensor (new Figure 1G). Importantly, we now provide evidence that the lack of apoptosis caused by Dronc deficiency is not responsible of the phenotypes (new Figure 2E-G). The text has been amended to incorporate the new information as follows:

"*Whereas the sensor-dependent induction of tomato-HA expression and GFP report on either the ongoing or recent past caspase activation, the presence of β-gal labelling without any of the other signals is the unambiguous demonstration of past caspase activation without apoptosis (Baena-Lopez et al., 2018) (EV Fig 1A). Intrigued by the previously described non-apoptotic activation patterns of effector caspases in response to thermal (cold shock) or metabolic stress (starvation) in the Drosophila ovary (Tang et al., 2015), we used DBS-S-QF to investigate whether such activation was linked to initiator caspases. Interestingly, experiments conducted with our sensor under moderate thermal stress (29°C) consistently labelled putative somatic cells in the germarium (Fig 1B). Furthermore, some DBS-S-QF positive cells did not show the fluorescent marker linked to ongoing caspase activation (tomato-HA), yet they expressed GFP (recent past caspase activation) without showing signs of apoptosis (TUNEL labelling; Fig 1B). These observations suggested the presence of transient and non-apoptotic bursts of caspase activation in the germarium. Supporting this hypothesis, we also noticed the presence of β-gal immunoreactivity in large populations of seemingly proliferative and therefore healthy ECs and FCs (Fig 1C; hereafter, raw data used for the quantitative analyses in the main Figures can be found in Appendix Table 1). Furthermore, the proportion of germaria permanently labelled with β-gal significantly increased over time in flies kept at 29°C (Fig 1D). Co-immunostaining with FasIII and β-gal also revealed that the permanent labelling increase was not linked to germaria only showing β-gal in the ECD (Fig 1E) but to germaria with combined labelling in the ECD and FCD (Fig 1F; EV Fig 1B). These results specifically correlated the increased DBS-S-QF labelling with the proliferative activity of FSCs.*"

2. The authors spent a lot of effort in Figure 1E-G to look at the expression of Dronc. However, the obtained expression pattern look somewhat different, especially 1E and 1F. What do they see with an antibody? Also, related to point #1, are these pattern stress induced?

These patterns are different because they showed either the transcriptional dynamics of Dronc (old figure 1E and 1F) or the stability of Dronc as a protein (old figure 1G). We attempted to simplify this information and explain the differences in the new version of the text as follows:

"Since our sensor was designed to detect the activation of initiator caspases, we next evaluated the dependency of the DBS-S-QF labelling with Dronc; main initiator Drosophila

caspase linked to non-apoptotic functions and ortholog of Caspase-2/9. The overexpression of a Dronc-RNAi transgene, under the control of ptc-Gal4, in all of the escort and follicular stem cells of the germarium reduced to residual levels the labelling with DBS-S-QF sensor (Fig 1G). Using a fly line expressing Gal4 under the physiological regulation of Dronc (Dronc$^{KO\text{-}Gal4}$) (Arthurton et al., 2019), we correlated our previous findings with a consistent transcriptional activation of Dronc in both follicular stem cells and their progeny at 29°C (ECs and FCs) (Figs 1H, 1I, and EV Fig 1C). Also, the so-called polar and stalk cells in more mature follicle chambers showed robust transcriptional activation of Dronc (Fig 1I, EV Fig 1C). Furthermore, taking advantage of a biotin ligase TurboID fused to Dronc (Shinoda et al., 2019), we detected an enrichment of Dronc in follicular (Fig 1J), polar, and stalk cells (EV Fig 1D). Collectively, these results strongly suggested that the ovarian follicular stem cells and their progeny (escort and follicular cells) can transiently activate Dronc in a non-apoptotic manner under moderate thermal stress (29°C)."

More importantly, we now show the dependency of caspase activation with Dronc (new Fig 1G).

3. The Fly-Fucci data in Fig 2 are not very well explained. As the authors mentioned, there are more cells in S phase, but are these data statistically significant? There seems to be a big discrepancy between the S-phase data in 2G and 2H. Also, the authors mention cells in G0 in the text, but the figure doesn't show any cells in G0. Do they mean G1?

We have included a new explanatory diagram describing the meaning of the Fly Fucci analyses (see new Figure H). We have also repeated the Fucci analysis using a UAS-Fucci construct only expressed in somatic cells. This has helped us to improve the resolution of our quantifications by avoiding the Fucci signal from the germline cells since we found this factor was polluting our initial analyses. The new data suggest that Dronc deficiency delays the progression of cell cycle during S-phase (new Figure 2I-K), thus slowing down the completion of the cell cycle. This provides an explanation for the reduced number of somatic cells observed upon limiting caspase activation in somatic cells. The text has been amended as follows to include all the new information:

"The Fly-Fucci construct reports on the cell cycle progression by labelling the different stages with distinguishable fluorescent markers (Zielke et al., 2014) (Fig 2H). This tool revealed that follicular cells with limited Dronc expression were accumulated in the S-phase at the expense of other cell cycle stages. (Figs 2E-G). The accumulation of cells in S-phase was also correlated with a significant increase in EdU labelling (S-phase marker; EV Fig 2L, hereafter, raw data used for the quantitative analyses in EV Figures can be found in Appendix Table 2). Since the excess of cells in the S-phase in these experiments did not increase the total number of FCs but caused a reduction (Fig 2C), we concluded that Dronc deficiency slows down the transition through the S-phase of FCs."

4. In Fig 3, the authors found that other components of the apoptosis pathway such as effector caspases have a similar phenotype when inactivated as does Dronc and they conclude that "...these experiments confirmed the non-apoptotic and pro-survival role of caspase activation..." (line 230). I disagree with the term "confirmed" here. Maybe the authors can use "more evidence" in this context, but confirmed is a little far fetched.

We have performed new experiments related to this section. Therefore, the suggestion of the reviewer has also been incorporated. The section of the manuscript is as follows:

"Next, we evaluated whether the implementation of Dronc functions could be mediated by the effector caspases; main Dronc substrates during apoptosis. To that end, we co-

overexpressed in the FSCs validated RNAi constructs able to reduce their expression (Drice, DCP-1, Decay and Damm) (Leulier et al., 2006) (EV Fig 3A). This genetic manipulation caused proliferation and differentiation phenotypes reminiscent of Dronc deficiency (Figs 3D-F). Since effector caspases can be functionally redundant (Xu et al., 2006), we separately assessed the contribution of DCP-1 and Drice to these phenotypes. Although these experiments also caused proliferation defects, the differentiation phenotypes were weaker (Figs 3D-F; notice the proportion of Castor-expressing cells versus the total number of follicular cells was reduced to a lesser extent), thus suggesting a likely implementation of Dronc functions mediated by the combinatorial activity of effector caspases. Supporting this hypothesis, the overexpression of either Diap-1 or P35 (a viral pan-effector caspase inhibitor (Hay et al., 1994)) also limited the proliferation and differentiation of ovarian somatic cells (EV Figs 3A-C). In these experiments, we used ptc-Gal4 to consistently reduce the activity of effector caspases in the entire ECD and the FSCs. In a different set of experiments, we evaluated the potential mechanisms of Dronc activation by ectopically expressing either a short-hairpin RNA against Dark (Drosophila orthologue of Apaf-1) (Obata et al., 2014) or a microRNA against the main pro-apoptotic factors (Hid, Reaper and Grim; RHG) (Siegrist et al., 2010), respectively (EV Fig 3A). Intriguingly, these experiments reduced the total number of follicular and Castor-positive cells (Fig 3D, 3E); however, the proportion of Castor-expressing cells versus the total was not affected (Fig 3F). These findings indicated that differentiation and proliferation phenotypes can be uncoupled. More importantly, our data provided evidence that the entire caspase pathway can be activated for non-apoptotic purposes in somatic cells of the ovary."

5. I like the data in Fig 4F-I (the rescue of the Dronc-/- phenotype by expression of SmoACT or Ci). Did the authors attempt the opposite experiment, rescuing the smo-/- or ci-/- phenotype by Dronc overexpression?

In the new version of the manuscript, we show that Dronc deficiency limits the ability of Hh ligand to signal (new EV Figure 4H-K). This new piece of information is correlated with an increase of Ptc within (Atg8+) autophagosomes (new Figure 5D-G) and induction of autophagy (new Figure 6A-I). Taking into consideration that Dronc deficiency phenotypes can be largely rescued by reducing the excess of autophagy in Dronc deficient cells (new Figure 6J-L), we conclude that *"the excess of autophagy is likely the primary defect caused by caspase deficiency, and this subsequently limits the efficient transduction of the Hh-pathway (Fig 6M). In this scenario, it is conceivable that the excess of autophagy in ovarian somatic cells without caspase activity could promote the degradation of Hh-pathway transduction components, thus limiting its signalling capacity (Fig 6M). Supporting this model, we have observed that the lack of autophagy is also sufficient to revert the proliferation and differentiation defects of FCs without caspase activation (Fig 6M)."*

In light of these new results, we decided to concentrate our experimental efforts in addressing other key questions raised by the reviewers instead of performing the suggested experiment.

6. The ptc data in Fig 4J,k are a little unclear and are inconsistent with the authors model. Maybe that is because the authors used double heterozygous ptc/Dronc conditions which I think can be a little tricky for epistasis experiments. What actually is the homozygous ptc-/- phenotype in these cells? Maybe that helps to resolve the counterintuitive result of the double heterozygous ptc/dronc interaction.

The new version of the manuscript shows how a reduction of Ptc in follicular cells deficient for Dronc is sufficient to restore their physiological properties (new Fig 5H-I). This is a strong indication suggesting that the intracellular accumulation of Ptc in autophagosomes results determinantal in ovarian somatic cells without Dronc (new Fig 5D-G). Consistently, a strong transcriptional downregulation of ptc also appears to prevent the excess of autophagy and

Hh-downregulation (EV Fig 5F). We agree with the reviewer about the counterintuitive nature of the genetic interaction between Ptc and Dronc but the new experimental data of the manuscript has helped to define it better.

"Intriguingly, recent reports have revealed a function of Ptc as a regulator of autophagy in the Drosophila ovary independent of Hh pathway (Jimenez-Sanchez et al., 2012, Singh et al., 2018). In mammalian cells, it has also been reported the autophagy-dependent degradation of Ptch1 (Yang et al., 2021). These findings prompted us to explore whether the Ptc aggregates observed in ptc-Gal4:Dronc germaria could be localised in autophagosomes. To this end, we performed colocalization experiments between Ptc and the autophagy regulator Atg8 (Slobodkin and Elazar, 2013). These experiments showed a significantly increased colocalization between Ptc and Atg8 in ptc-Gal4:Dronc germaria (Figs 5D, 5E), a higher proportion of Ptc in Atg8+ autophagosomes (Fig 5F), and more Atg8+ autophagosomes loaded with Ptc (Fig 5G). To assess the biological significance of Ptc accumulation in autophagosomes, we reduced Ptc protein levels without affecting its molecular features by overexpressing a Ptc RNAi construct in FSCs. These experiments rescued the proliferation and differentiation defects shown by FCs without Dronc (Figs 5H, 5I). A compatible phenotypic rescue was obtained by using a heteroallelic combination for ptc that reduced the transcription efficiency of the gene without affecting the molecular structure of the protein (EV Fig 5F). Collectively, our experiments revealed a previously unrecognised ability of Dronc to prevent the accumulation of Ptc in autophagosomes. They also underscored the relevance of limiting the intracellular accumulation of Ptc to maintain the homeostasis of ovarian somatic cells."

7. Fig 6: In addition to Ref2P, did the authors test other autophagy markers such as Atg8-GFP or some autophagic flux markers? That would strengthen the results about the role of caspases for autophagy.

We are really thankful for this valuable suggestion of the reviewer. In line with the reviewer's suggestion, we have evaluated the potential colocalisation of Ptc with Atg8. The new data suggests that Ptc is prominently accumulated in Atg8+ autophagosomes in Dronc deficient conditions (Figure 5D-G). Furthermore, Atg8 autophagosomes accumulating Ptc are also lager and more abundant (see new Figure 6A-D). These experiments strongly suggest that Ptc is diverted into Atg8 autophagosomes in ovarian somatic cells without normal levels of Dronc. New functional experiments have also shown that a reduced Atg8 expression is sufficient to rescue the proliferation and differentiation defects caused by Dronc deficiency in follicular cells (new Figure 6J-L). Together these results provide more evidence suggesting that Dronc activation prevents autophagy excess that ultimately sustains Hh-signalling. Please see discussion of the manuscript.

8. Do Dronc mutants have an autophagic phenotype in this tissue?

Yes, that seems to be case. The lack of Dronc promotes an excess of early autophagosomes (new Fig 6A-D) and a reduction of Ref2P (6E-I). Also, the increase number of autophagosomes is correlated with an accumulation of Ptc inside (new Fig 5D-G). All this information is compatible with an excess of autophagy upon limiting Dronc expression in ovarian somatic cells under thermal stress.

9. I am a little confused by the result in EV Fig 2K. I am surprised that there are TUNEL-positive cells in Dronc-/- germaria. Aren't Dronc mutants completely lacking apoptosis? Can you actually show these data?

We really appreciate this comment from the reviewer and we have to apologize for the way the information was initially obtained since it did not distinguish the apoptotic effects occurring in the germline and the somatic cells. The new version of the manuscript includes a completely new experiment with suitable genetic markers to distinguish the effects in different cellular domains and a vast collection of data to ensure the biological significance of the results. Raw data of these quantifications can be found in the corresponding Appendix table. As shown in the new Figure 2E-G, Dronc deficiency reduces the TUNEL labelling in a dose dependent manner in somatic cells, while the overexpression induces cell death (new Figure 2E). Intriguingly, both experiments also induced non-autonomous cell death in the germline. Three main conclusions can be extracted from these results. 1) Despite reducing apoptosis, the lack of Dronc in somatic cells limits the number of follicular somatic cells, thus supporting its pro-proliferative role. 2) This function of Dronc is likely true only under a moderate threshold of activation since the excess also causes cell death. 3) The manipulation of Dronc expression in somatic cells can cause apoptosis in the germline by possibly interfering with the molecular mechanisms coordinating the activity between somatic and germline cells (Shi et al., 2021, Sahai-Hernandez et al., 2012). The latter would be to some extent expected since Hh-signalling is key for this intercellular coordination of functions (e.g. 10.1016/j.stemcr.2018.07.008). All the new data have been incorporated in the new version of the manuscript as follows.

"To better determine the origin of the reduction in number of follicular cells, we analysed the rate of apoptosis and the cell cycle profile in our mutant conditions. Predictably, low levels of Dronc in somatic cells reduced that rate of apoptosis in the germarium while the overexpression induced cell death (reduction in TUNEL staining, Fig 2E). These genetic manipulations also induced an intriguing non-autonomous excess of apoptosis in the germline (notice the TUNEL staining in groups of cells negative for somatic markers such as FasIII and Histone-RFP; arrows; Fig 2F, 2G) that could be indicative of cell communication defects between somatic cells and the germline (Shi et al., 2021, Sahai-Hernandez et al., 2012). These results dissociated the growth and differentiation phenotypes induced by caspase deficiency from the process of apoptosis itself since one would expect an excess of follicular cells upon inhibiting apoptosis, as opposed to a reduction."

Representative images illustrating the results of the graph has been incorporated in the text (new Figure 2E-G).

Referee #3:

In this manuscript the authors examine a non-apoptotic role for caspases in survival and differentiation of somatic tissues in stressed Drosophila ovaries. This is an important process that is potentially conserved between flies and humans. The researchers use state of the art approaches, and the data are in general of high quality. The results are interpreted quite aggressively so there are some concerns about particular conclusions. In addition, this is a very dense paper that while accessible to Drosophila researchers may not be to a general audience.

The authors use a clever reporter system to identify cells in which the Drosophila caspase Drice is active, was recently active, or active in the past. They show that in animals that have been stressed at 29C, Drice is activated in ovarian escort cells, but that not all of these cells undergo apoptosis and can give rise to pre-follicular cells.

This section of the manuscript has been refurbished to incorporate new experimental data as follows:

"*Whereas the sensor-dependent induction of tomato-HA expression and GFP report on either the ongoing or recent past caspase activation, the presence of β-gal labelling without*

any of the other signals is the unambiguous demonstration of past caspase activation without apoptosis (Baena-Lopez et al., 2018) (EV Fig 1A). Intrigued by the previously described non-apoptotic activation patterns of effector caspases in response to thermal (cold shock) or metabolic stress (starvation) in the Drosophila ovary (Tang et al., 2015), we used DBS-S-QF to investigate whether such activation was linked to initiator caspases. Interestingly, experiments conducted with our sensor under moderate thermal stress (29°C) consistently labelled putative somatic cells in the germarium (Fig 1B). Furthermore, some DBS-S-QF positive cells did not show the fluorescent marker linked to ongoing caspase activation (tomato-HA), yet they expressed GFP (recent past caspase activation) without showing signs of apoptosis (TUNEL labelling; Fig 1B). These observations suggested the presence of transient and non-apoptotic bursts of caspase activation in the germarium. Supporting this hypothesis, we also noticed the presence of β-gal immunoreactivity in large populations of seemingly proliferative and therefore healthy ECs and FCs (Fig 1C; hereafter, raw data used for the quantitative analyses in the main Figures can be found in Appendix Table 1). Furthermore, the proportion of germaria permanently labelled with β-gal significantly increased over time in flies kept at 29°C (Fig 1D). Co-immunostaining with FasIII and β-gal also revealed that the permanent labelling increase was not linked to germaria only showing β-gal in the ECD (Fig 1E) but to germaria with combined labelling in the ECD and FCD (Fig 1F; EV Fig 1B). These results specifically correlated the increased DBS-S-QF labelling with the proliferative activity of FSCs."

Using another clever construct, they go on to show that Dronc, an activator caspase of Drice, is expressed in escort and pre-follicular cells of heat stressed ovaries. Elimination of Dronc from follicular stem cells in heat stressed ovaries leads to a significant decrease in the number of pre-follicular cells and the number of these cells expressing the differentiation marker Castor. While the cell cycle distribution was not dramatically altered in Dronc mutants, there was a significant increase in the number of EdU positive cells. They also show that the enzymatic activity of Dronc was necessary for proliferation and differentiation.

We have included a new explanatory diagram describing the meaning of the Fly Fucci analyses (see new Figure H). We have also repeated the Fucci analysis using a UAS-Fucci construct only expressed in somatic cells. This has helped us to improve the resolution of our quantifications by avoiding the Fucci signal from the germline cells since we found this factor was polluting our initial analyses. The new data suggest that Dronc deficiency delays the progression of cell cycle during S-phase (new Figure 2I-K), thus slowing down the completion of the cell cycle. This provides an explanation for the reduced number of somatic cells observed upon limiting caspase activation in somatic cells. The text has been amended as follows in order to include the new information:

"The Fly-Fucci construct reports on the cell cycle progression by labelling the different stages with distinguishable fluorescent markers (Zielke et al., 2014) (Fig 2H). This tool revealed that follicular cells with limited Dronc expression were accumulated in the S-phase at the expense of other cell cycle stages. (Figs 2E-G). The accumulation of cells in S-phase was also correlated with a significant increase in EdU labelling (S-phase marker; EV Fig 2L, hereafter, raw data used for the quantitative analyses in EV Figures can be found in Appendix Table 2). Since the excess of cells in the S-phase in these experiments did not increase the total number of FCs but caused a reduction (Fig 2C), we concluded that Dronc deficiency slows down the transition through the S-phase of FCs."

Using a combination of UAS-RNAi constructs, the authors disrupted the initiators of programmed cell death Hid, Grim and Reaper. This had a significant effect on survival but not differentiation. The authors argue that this shows that the regulation of these processes is distinct, but it could just reflect one process being less sensitive to knock down of Hid, Grim and Reaper.

The reviewer is totally correct on this point and we have toned down our statement. The test has been amended as follows:

"Since effector caspases can be functionally redundant (Xu et al., 2006), we separately assessed the contribution of DCP-1 and Drice to these phenotypes. Although these experiments also caused proliferation defects, the differentiation phenotypes were weaker (Figs 3D-F; notice the proportion of Castor-expressing cells versus the total number of follicular cells was reduced to a lesser extent), thus suggesting a likely implementation of Dronc functions mediated by the combinatorial activity of effector caspases. Supporting this hypothesis, the overexpression of either Diap-1 or P35 (a viral pan-effector caspase inhibitor (Hay et al., 1994)) also limited the proliferation and differentiation of ovarian somatic cells (EV Figs 3A-C). In these experiments, we used ptc-Gal4 to consistently reduce the activity of effector caspases in the entire ECD and the FSCs. In a different set of experiments, we evaluated the potential mechanisms of Dronc activation by ectopically expressing either a short-hairpin RNA against Dark (Drosophila orthologue of Apaf-1) (Obata et al., 2014) or a microRNA against the main pro-apoptotic factors (Hid, Reaper and Grim; RHG) (Siegrist et al., 2010), respectively (EV Fig 3A). Intriguingly, these experiments reduced the total number of follicular and Castor-positive cells (Fig 3D, 3E); however, the proportion of Castor-expressing cells versus the total was not affected (Fig 3F). These findings indicated that differentiation and proliferation phenotypes can be uncoupled. More importantly, our data provided evidence that the entire caspase pathway can be activated for non-apoptotic purposes in somatic cells of the ovary."

As previous work had demonstrated a role for Hedgehog signaling in the survival and differentiation of the somatic tissues of the ovary, the authors examined the relationship between caspase activation in stressed ovaries and the Hh pathway. They show that expression of activated Smo or over expression of Ci could rescue both the survival and differentiation defects in Dronc mutant cells. The also show a decrease in cells expressing the differentiation marker Castor in animals heterozygous for ptc-GAL4 and Dronc[-]. The authors point out both that ptc-GAL4 is a hypomorphic mutation of ptc and that the sign of this genetic interaction doesn't make sense. Ptc is a negative regulator of the pathway; given the results with activated Smo and Ci, one would expect that lower Ptc levels would improve Dronc heterozygotes. With this surprising relationship, it is important to demonstrate that the interaction with Dronc is actually due to the GAL4 insertion in ptc. The GAL4 insertion in ptc is a P-element vector and therefore it can be excised using transposase. The authors should excise the P-element and determine whether the genetic interaction with Dronc is eliminated. Given that P-element activation can initiate recombination even in males, this excision should be done over a balancer.

We consider unnecessary the experiment suggested by the reviewer since similar effects have been observed using a different allele of *ptc* (*ptc*^{S2}) (new EV Figure 4M-N). Also the overexpression of Dronc is sufficient to rescue to defects observed in *ptc*-Gal4: *Dronc*^{KO} germaria (Fig 4J-K). Moreover, a reduction of Ptc protein levels is sufficient to rescue the phenotypes caused by Dronc deficiency (5H-I). The text has been amended in different sections to better convey the information related to the counterintuitive interaction between Ptc and Dronc.

In characterizing the ptc-GAL4 Dronc[-] heterozygotes the authors noted elevated Ptc protein puncta. The accumulation of Ptc was important not only for the Castor differentiation phenotype but also the survival phenotype as RNAi to ptc rescued both of these defects in Dronc mutant cells. The accumulation of Ptc puncta could correlate with the ability of Ptc to induce autophagy. In ptc-GAL4 Dronc heterozygotes Ref2P puncta (which accumulate when autophagy is blocked) decrease suggesting increased autophagy in this genetic combination. That increased autophagy was required for the decreased expression of the

differentiation marker Castor, was tested by blocking autophagy in the ptc-GAL4 Dronc heterozygous background using RNAi to Atg1. In this combination expression of Castor was restored.

The authors make the point that autophagy is involved in differentiation and not survival, but they only do the experiments in the ptc-GAL4 Dronc heterozygous background. It would also be worthwhile to include knockdown Atg1 in Dronc-/- cells (which show a defect in cell survival) to demonstrate that Atg1 doesn't have a cell survival effect. When doing these experiments, it would be good to look at Ptc protein accumulation in Dronc-/- cells vs. Dronc-/- cells with RNAi to Atg1. These experiments might sort out the relationship between Ptc accumulation and induction of autophagy.
This is potentially a very interesting paper. The two major concerns are resolving the genetic relationship between ptc-GAL4 and Dronc- and the relationship between Ptc and autophagy.

We are really thankful for this valuable suggestion of the reviewer. In line with the reviewer's suggestion, we have evaluated the potential colocalisation of Ptc with Atg8. The new data suggests that Ptc is prominently accumulated in Atg8+ autophagosomes in Dronc deficient conditions (Figure 5D-G). Furthermore, Atg8 autophagosomes accumulating Ptc are also larger and more abundant (see new Figure 6A-D). In parallel, we have observed a reduction of Ref2P (6E-I). All this information is compatible with a Ptc-dependent excess of autophagy upon limiting Dronc expression in ovarian somatic cells under thermal stress.
New functional experiments have also shown that a reduced Atg8 expression is sufficient to rescue the proliferation and differentiation defects caused by Dronc deficiency in follicular cells (new Figure 6J-L). Together these results provide more evidence suggesting that Dronc activation prevents autophagy excess that ultimately sustains Hh-signalling. Please see discussion of the manuscript.

Referee #4:

In this study, the authors investigate the non-apoptotic roles of caspases in the follicle epithelium in the Drosophila ovary. They find that, in flies maintained at 29C which they consider a moderate stress, caspases are activated but the cells do not undergo apoptosis. Loss of the caspase, dronc, causes a decrease in proliferation relative to wildtype cells and loss of expression of cas, which promotes differentiation in this lineage. These observations contribute to the emerging understanding that caspases have functions other than promoting apoptosis. The authors then provide evidence that dronc exerts these effects through an interaction with the hedgehog pathway. Specifically, dronc promotes hedgehog signaling by preventing the accumulation of ptc. The authors also show that some of the effects they see in the fly ovary are conserved in a human ovarian somatic cell line. Taken together, the observations in this study are provocative, and I think would be of value to the community. I found the images to be convincing for the most part and appreciated the careful quantification of the phenotypes throughout. However, there are several issues that should be addressed before publication.

1. The study is framed as an investigation of the role that caspases play in proliferation and differentiation during moderate stress, but it is not clear that 29C is a moderate stress, and evidence is not provided to show that these effects are specific to the 29C condition. The authors should test whether the activation of the caspase reporter, the requirements for dronc in proliferation and cas expression, and the effects on hedgehog signaling are also observed at a standard temperature (e.g. 25C). In addition, they should provide other evidence (or describe published data) indicating that 29C is, in fact, a stressful condition.

We thank the reviewer for raising this point since it has helped us to better understand the dynamics of caspase activation in our system. The new version of the manuscript describes in detail the caspase activation at 25°C and at 29°C in different types of somatic cells. These experiments suggest that non-apoptotic caspase activation is potently boosted under moderate thermal stress (29°C) (new Figure 1D-F). We have also confirmed that such caspase activation relies on Dronc since deficiency of this factor prevents the labelling of somatic cells with our caspase sensor (new Figure 1G). Importantly, we now provide evidence that the lack of apoptosis caused by Dronc deficiency is not responsible of the phenotypes (new Figure 2E-G). The text has been amended to incorporate the new information as follows:

"*Whereas the sensor-dependent induction of tomato-HA expression and GFP report on either the ongoing or recent past caspase activation, the presence of β-gal labelling without any of the other signals is the unambiguous demonstration of past caspase activation without apoptosis (Baena-Lopez et al., 2018) (EV Fig 1A). Intrigued by the previously described non-apoptotic activation patterns of effector caspases in response to thermal (cold shock) or metabolic stress (starvation) in the Drosophila ovary (Tang et al., 2015), we used DBS-S-QF to investigate whether such activation was linked to initiator caspases. Interestingly, experiments conducted with our sensor under moderate thermal stress (29°C) consistently labelled putative somatic cells in the germarium (Fig 1B). Furthermore, some DBS-S-QF positive cells did not show the fluorescent marker linked to ongoing caspase activation (tomato-HA), yet they expressed GFP (recent past caspase activation) without showing signs of apoptosis (TUNEL labelling; Fig 1B). These observations suggested the presence of transient and non-apoptotic bursts of caspase activation in the germarium. Supporting this hypothesis, we also noticed the presence of β-gal immunoreactivity in large populations of seemingly proliferative and therefore healthy ECs and FCs (Fig 1C; hereafter, raw data used for the quantitative analyses in the main Figures can be found in Appendix Table 1). Furthermore, the proportion of germaria permanently labelled with β-gal significantly increased over time in flies kept at 29°C (Fig 1D). Co-immunostaining with FasIII and β-gal also revealed that the permanent labelling increase was not linked to germaria only showing β-gal in the ECD (Fig 1E) but to germaria with combined labelling in the ECD and FCD (Fig 1F; EV Fig 1B). These results specifically correlated the increased DBS-S-QF labelling with the proliferative activity of FSCs.*"

Beyond our results, a recent detailed characterisation of the effect of temperature shows that at *29°C* there is a reduced rate of egg deposition, vitellogenesis and cysts survival, thus indicating this temperature is suboptimal or stressful(https://doi.org/10.1242/dev.200149).

2. The effect of dronc knockout on the number of cells in the FasIII domain expressing cas (e.g. Fig. 2C and 3C) is not well described. FasIII expression extends well beyond the germarium, but the images provided and the number of cells counted in controls suggest that the analysis was limited to the germarium. The authors should provide more information about how the posterior boundary of the region to be analyzed was determined. In Fig. 2b, the lower number of follicle cells may be a secondary effect caused by a defect in budding from the germarium, in which case considering only the FasIII follicle cells in the germarium is somewhat arbitrary.

The new version of the manuscript shows explanatory diagrams, figures, and descriptions of the cells under investigation. Our quantifications have always been made in germaria without budding follicles; this particular specific information has been added to the figure legends.

3. On a related note, it is not clear what effect the loss of cas expression has on the follicle epithelium . Are there defects in specification of the polar, stalk, and/or main body cell fates? Is eya expression affected? What is the significance of this effect for the tissue function?

The data is not shown in the manuscript for simplification purposes, but the effects caused by Dronc deficiency in the germarium often lead to aberrant egg encapsulation, egg polarity defects, altered specification of polar cells, and egg degeneration in advanced stages of development (please see figure below for the referee)[Figures for referees not shown.]. However, we have not done a quantitative and detailed phenotypic analysis of these phenotypes since they are secondary to what is happening in the germarium and therefore out of the main goal of the study.

4. What is the rationale for switching from 190-30-Gal4 in Fig. 3A-B to ptc-Gal4 in Fig. 3C-D? These drivers do not have the same expression pattern in the germarium and, in subsequent figures, the authors show that heterozygosity for ptc-Gal4 causes phenotypes. Thus, it is difficult to compare the results with 109-30-Gal4 vs. ptc-Gal4, as the authors attempt to do on p. 6.

190-30-Gal4 was used to efficiently target the expression of Dronc in follicular stem cells and follicular cells, thus keeping unaffected most of the escort cells. *ptc-Gal4* was used to robustly manipulate the genetic configuration of follicular stem cells and the entire cohort of escort cells. This distinction has allowed us to better define the role of Dronc in somatic cells. Incidentally, it has also uncovered a potent genetic interaction between Dronc and Ptc. This information has been conveyed in the new version of the text as follows:

" To circumvent these technical limitations, we capitalised on several Dronc conditional alleles generated in the laboratory through genome engineering (Arthurton et al., 2019) (EV Fig 2A). These alleles enable the efficient conversion of heterozygous wild-type cells containing an FRT-Dronc-FRT rescue cassette into homozygous mutant upon exposure to the Flippase recombinase (EV Fig 2B) (Arthurton et al., 2019). The FRT-Dronc-FRT rescue cassette was followed by the QF transcriptional activator in one of these alleles, thus allowing the expression of any cDNA of interest under the physiological regulation of Dronc upon cassette excision (EV Fig 2B). Combining this allele with the 109-30-Gal4 follicular driver (EV Fig 2C, 2D) (Sahai-Hernandez and Nystul, 2013), we efficiently eliminated Dronc expression (100% of inspected germaria, n=14) in the prospective FCD and a subset of ECs (EV Fig 2E). Interestingly, these genetic manipulations caused a characteristic set of morphological and genetic defects in the germarium (compare Figs 2A and 2B; compare EV Figs 2E and 2F). Specifically, the total number of follicular cells and the total number of Castor-positive cells in the FCD were significantly reduced (Fig 2C). Furthermore, Castor

expression was downregulated since the proportion of Castor-positive cells versus the total of FCs also diminished (Fig 2D). Equivalent results were obtained by eliminating Dronc expression in all of the escort cells and follicular stem cells with ptc-Gal4 (Hartman et al., 2015) (Fig 2C, 2D and EV Figs 2I-K)."

5. The cas phenotype in ptc/+, dronc/+ (EV Fig. 4D) is not very convincing. The pattern of expression looks similar to wildtype. This phenotype should be quantified in the same way that it was in other parts of the paper (e.g. Fig. 2 and 3).

We now provide quantifications of the effects observed in different ptc/+, dronc/+ conditions in the manuscript (new EV Figure 4M-N). We also show that the overexpression of Dronc is sufficient to rescue to defects observed in *ptc*-Gal4: *Dronc*^{KO} germaria (Fig 4J-K) and inversely, a reduction of Ptc protein levels can rescue the phenotypes caused by Dronc deficiency (5H-I). Finally, we show how a reduction of Ptc protein levels or Ptc-dependent autophagy rescue the defects caused by Dronc deficiency. These experiments support the specificity of a counterintuitive interaction between Ptc and Dronc. The molecular origin such interaction appears to be linked to the ability of Dronc to prevent the diversion of Ptc into autophagosomes and the subsequent Ptc-dependent induction of autophagy (please, see new figures 5 and 6).

6. The labels for FSCs in Fig. 1G are not correct. FSCs are located to the anterior of Region 2b, but the lines are indicating positions that are within Region 2b, well downstream of the anterior face of the Region 2b cyst.

We really appreciate this comment from the reviewer since as indicated the labelling in our diagram was not correct. This imprecision has been amended in the new version of the manuscript (please see new Figure 1).

7. The ptc-Gal4, dronc +/- germarium in Fig. 5A seems much bigger than the other germaria in the other two images. Are there more escort cells in this genotype?

The difference in size is due to the fact that in our mutant conditions there is a variable accumulation over time of germline cysts, likely due to a small rate of encapsulation caused by the reduced number of FSCs progeny. These phenotypes have not been included in the manuscript for simplification purposes since they would complicate the phenotypic characterisation and they are non-cell autnomous. The effect of this germline accumulation can cause an increase of total size of the germarium. If relevant for the general argumentation of the manuscript, we can include this information in the manuscript.

The number of ptc puncta should be normalized to EC number or evidence should be provided that EC number is not significantly different between these genotypes.

The new version of the manuscript provides does not include the normalisation suggested by the reviewer, instead we provide a detailed characterisation of Ptc immunoreactive puncta and where are located within somatic cells. Our new data indicates that Ptc appears to be accumulated in Atg8+ autophagosomes in Dronc deficient conditions (Figure 5D-G). Furthermore, Atg8 autophagosomes accumulating Ptc are larger and more abundant (see new Figure 6A-D). In parallel, we have observed a reduction of Ref2P (6E-I). All this information is compatible with a Ptc-dependent excess of autophagy upon limiting Dronc expression in ovarian somatic cells under thermal stress. New functional experiments have also shown that a reduced atg8 expression is sufficient to rescue the proliferation and differentiation defects caused by Dronc deficiency in follicular cells (new Figure 6J-L). Together these results provide more evidence suggesting that Dronc activation prevents the accumulation of Ptc in autophagosomes and the Ptc-dependent excess of autophagy. These

effects ultimately appear to sustain Hh-signalling and the homeostasis of ovarian somatic cells. This information has been incorporated in different sections of the text and have made us to dismiss the normalisation method suggested by the reviewer.

2nd Revision - Editorial Decision **16th Feb 2023**

Dear Alberto,

Thank you for the submission of your revised manuscript to EMBO reports. We have now received the full set of referee reports that is copied below.

As you will see, all referees are very positive about the study and request only minor changes to clarify text and figures and they recommend providing the Image J macro.

From the editorial side, there are also a few things that we need before we can proceed with the official acceptance of your study.

- Please reduce the number of keywords to 5

- Please reduce the number of Expanded View Figures to 5, since we cannot typeset more than 5. You could either merge two figures, although they appear quite data-rich, so I am not sure merging is the best option. You could also move one figure to the Appendix, or alternatively show all 6 figures in the Appendix. For your info, the Appendix is a single PDF file called *Appendix*, which should start with a short Table of Content incl. page numbers. Appendix figures should be referred to in the main text as: "Appendix Figure S1, Appendix Figure S2" etc. See detailed instructions regarding expanded view here: <https://www.embopress.org/page/journal/14693178/authorguide#expandedview>

- The Data availability section should only refer to data deposited in public repositories. If no data has been deposited, you should state this. You currently refer to raw data shown in Appendix tables. Please note that raw data (quantifications, Western blots etc) are linked to your manuscript as 'Source data'. For this we need the source data split into one file per figure. This applies to the quantification and the Western blot data. You can put all files that belong to one figure into one folder and ZIP it. Then the source data for the main figures should be ZIPd together, and EV Figure source data should be ZIPd together.
I attach here a document 'SD-FAQs" that describes this in more detail.

- The actin blot for Figure 6H in the source data seems to have a longer exposure than the one chosen for the figure.

- Please update the 'Conflict of interest' paragraph to our new 'Disclosure and competing interests statement'. For more information see
https://www.embopress.org/page/journal/14693178/authorguide#conflictsofinterest

- Regarding the Author Contributions, we now use CRediT to specify the contributions of each author in the journal submission system. CRediT replaces the author contribution section, which therefore needs to be removed from the manuscript text. You can use the free text box in our system if you wish to provide more detailed descriptions. See also guide to authors https://www.embopress.org/page/journal/14693178/authorguide#authorshipguidelines.

- Please update the references to match the style in EMBO reports papers. The abbreviation 'et al' should be used if more than 10 authors. You can download the respective EndNote file from our Guide to Authors
https://endnote.com/style_download/embo-reports/

- Author checklist: Please complete the information in the top left corner (Author name, Journal, manuscript number).

- Biorender: Please provide the license by e-mail or upload it as related manuscript file.
- For the synopsis image you should insert in small font (approx. 7.5 at final image size of 550 pixels width) 'Created with BioRender'. I noticed that Biorender provides Patched as 7-pass TM protein, while it has actually 12 TMs - you might consider changing its graphical representation.
- For Fig. EV2A/B, EV6A and Fig. 6M, please include the statement that these were generated with BioRender in the respective Figure legends.

- The bioRxiv reference Melamed et al 2022 needs to be marked as preprint. In the text, the citation should be (preprint: NAME1 et al, YEAR). In the reference list it should look this way: Author NAME1 etc (YEAR) article title. bioRxiv doi [PREPRINT]

- Fly lines used: I recommend adding a table of the fly lines and their genotypes to the materials and methods section or to the Appendix as finding this information in the figure legends only seems a bit more complicated.

- Callouts to Figure panels 2I-K, and EV2G&H are missing.
- There are callouts to 2 Appendix tables, which should be removed after the data has been formatted/supplied as Source data.

- I attach to this email a related manuscript file with comments by our data editors. Please address all comments and upload a revised file with tracked changes with your final manuscript submission.

We look forward to seeing a final version of your manuscript as soon as possible.

With kind regards,

Martina

Martina Rembold, PhD
Senior Editor
EMBO reports

****

Referee #1:

The revised manuscript is much improved and the authors went the extra mile to address all of my original concerns. It is also reads more smoothly. The findings are novel and intriguing and I therefore fully recommend publication in EMBO reports.

Referee #2:

The authors have done an excellent job to address my comments and concerns. The manuscript is now much easier to follow than the original submission. I have no further experimental requests, but a few formal ones:

1. line 98: Explain in the text what DBS-S stands for.
2. line 192/3: I think the authors refer to Figs 2I-K
3. line 338: "live of observation". I think the work "of" needs to be deleted
4. line 419: delete the last word ("in")
line 427: delete the "d" in rescued

Referee #3:

The authors made a very detailed response to the previous review, have added appropriate experiments, and made the manuscript more accessible to a general audience. This is a very substantial paper that addresses an important question. I have no further concerns.

Referee #4:

This is a beautiful study and it has been further strengthened by the response to reviews. The authors have addressed my concerns, and I am now supportive of publication. One minor point is that the file for the custom ImageJ Cell Counter macro used in this study does not appear to have been included. It should be made available upon publication, along with a description of its use in the methods. Ideally, it would also be nice to include one or more representative images that demonstrate the efficacy of the method.

We appreciate the comments from the reviewers and their enthusiasm for our work. We have made all the editorial changes requested. In addition, we have inserted the following changes:

- We have generated two new tables that are included in the Appendix file describing the genotypes and properties of fly strains used in our experiments.

- Finally, we have decided to move to the Appendix file (new Appendix Figure S1) the previous Figure EV6 since it contains additional information to the data in the main Figure 6.

- We have to apologize because our original description regarding the quantification method was unfortunate since we did not write any script for it. Indeed, we manually quantified the number of follicular cells for each data set using the Plug-in/macro contained in ImageJ call Cell counter. The current description of methods clearly states our quantification protocol and it has been supported by an example in the Appendix file (new Appendix Figure S2). Although it is more time-consuming than having an automated script has been very effective to count individual cells in the ROI.

Thank you very much for your attention

Kind regards

Alberto

****

Referee #1:

The revised manuscript is much improved and the authors went the extra mile to address all of my original concerns. It is also reads more smoothly. The findings are novel and intriguing and I therefore fully recommend publication in EMBO reports.

Referee #2:

The authors have done an excellent job to address my comments and concerns. The manuscript is now much easier to follow than the original submission. I have no further experimental requests, but a few formal ones:

1. line 98: Explain in the text what DBS-S stands for.
2. line 192/3: I think the authors refer to Figs 2I-K
3. line 338: "live of observation". I think the work "of" needs to be deleted
4. line 419: delete the last word ("in")

line 427: delete the "d" in rescued

Referee #3:

The authors made a very detailed response to the previous review, have added appropriate experiments, and made the manuscript more accessible to a general audience. This is a very substantial paper that addresses an important question. I have no further concerns.

Referee #4:

This is a beautiful study and it has been further strengthened by the response to reviews. The authors have addressed my concerns, and I am now supportive of publication. One minor point is that the file for the custom ImageJ Cell Counter macro used in this study does not appear to have been included. It should be made available upon publication, along with a description of its use in the methods. Ideally, it would also be nice to include one or more representative images that demonstrate the efficacy of the method.
<SD_FAQs_EMBO_Press_1676561193_14.pdf><EMBOR-2020-51716V2-Data_edited_MS_file_1676561217_37.docx>

Prof. Luis Alberto Baena Lopez
University of Oxford
Sir William Dunn School of Pathology
South Parks Road
Oxford UK
Oxford, State... Ox13RE
United Kingdom

Dear Alberto,

I am very pleased to accept your manuscript for publication in the next available issue of EMBO reports. Thank you for your contribution to our journal.

At the end of this email I include important information about how to proceed. Please ensure that you take the time to read the information and complete and return the necessary forms to allow us to publish your manuscript as quickly as possible.

As part of the EMBO publication's Transparent Editorial Process, EMBO reports publishes online a Review Process File to accompany accepted manuscripts. As you are aware, this File will be published in conjunction with your paper and will include the referee reports, your point-by-point response and all pertinent correspondence relating to the manuscript.

If you do NOT want this File to be published, please inform the editorial office within 2 days, if you have not done so already, otherwise the File will be published by default [contact: emboreports@embo.org]. If you do opt out, the Review Process File link will point to the following statement: "No Review Process File is available with this article, as the authors have chosen not to make the review process public in this case."

Thank you again for your contribution to EMBO reports and congratulations on a successful publication. Please consider us again in the future for your most exciting work.

Kind regards,

Martina

Martina Rembold, PhD
Senior Editor
EMBO reports

****

THINGS TO DO NOW:

Please note that you will be contacted by Wiley Author Services to complete licensing and payment information. The required 'Page Charges Authorization Form' is available here: https://www.embopress.org/pb-assets/embo-site/er_apc.pdf - please download and complete the form and return to embopressproduction@wiley.com

EMBO Press participates in many Publish and Read agreements that allow authors to publish Open Access with reduced/no publication charges. Check your eligibility: https://authorservices.wiley.com/author-resources/Journal-Authors/open-access/affiliation-policies-payments/index.html

You will receive proofs by e-mail approximately 2-3 weeks after all relevant files have been sent to our Production Office; you should return your corrections within 2 days of receiving the proofs.

Please inform us if there is likely to be any difficulty in reaching you at the above address at that time. Failure to meet our deadlines may result in a delay of publication, or publication without your corrections.

All further communications concerning your paper should quote reference number EMBOR-2020-51716V3 and be addressed to emboreports@wiley.com.

Should you be planning a Press Release on your article, please get in contact with emboreports@wiley.com as early as possible, in order to coordinate publication and release dates.

EMBO Press Author Checklist

Corresponding Author Name: Luis Alberto Baena	
Journal Submitted to: EMBO Reports	
Manuscript Number: EMBOR-2020-51716V3	

USEFUL LINKS FOR COMPLETING THIS FORM
The EMBO Journal - Author Guidelines
EMBO Reports - Author Guidelines
Molecular Systems Biology - Author Guidelines
EMBO Molecular Medicine - Author Guidelines

Reporting Checklist for Life Science Articles (updated January 2022)
This checklist is adapted from Materials Design Analysis Reporting (MDAR) Checklist for Authors. MDAR establishes a minimum set of requirements in transparent reporting in the life sciences (see Statement of Task: 10.31222/osf.io/9sm4x). Please follow the journal's guidelines in preparing your manuscript.
Please note that a copy of this checklist will be published alongside your article.

Abridged guidelines for figures
1. Data
The data shown in figures should satisfy the following conditions:
- the data were obtained and processed according to the field's best practice and are presented to reflect the results of the experiments in an accurate and unbiased manner.
- ideally, figure panels should include only measurements that are directly comparable to each other and obtained with the same assay.
- plots include clearly labeled error bars for independent experiments and sample sizes. Unless justified, error bars should not be shown for technical replicates.
- if n<5, the individual data points from each experiment should be plotted. Any statistical test employed should be justified.
- Source Data should be included to report the data underlying figures according to the guidelines set out in the authorship guidelines on Data Presentation.

2. Captions
Each figure caption should contain the following information, for each panel where they are relevant:
- a specification of the experimental system investigated (eg cell line, species name).
- the assay(s) and method(s) used to carry out the reported observations and measurements.
- an explicit mention of the biological and chemical entity(ies) that are being measured.
- an explicit mention of the biological and chemical entity(ies) that are altered/varied/perturbed in a controlled manner.
- the exact sample size (n) for each experimental group/condition, given as a number, not a range;
- a description of the sample collection allowing the reader to understand whether the samples represent technical or biological replicates (including how many animals, litters, cultures, etc.).
- a statement of how many times the experiment shown was independently replicated in the laboratory.
- definitions of statistical methods and measures:
  - common tests, such as t-test (please specify whether paired vs. unpaired), simple χ2 tests, Wilcoxon and Mann-Whitney tests, can be unambiguously identified by name only, but more complex techniques should be described in the methods section;
  - are tests one-sided or two-sided?
  - are there adjustments for multiple comparisons?
  - exact statistical test results, e.g., P values = x but not P values < x;
  - definition of 'center values' as median or average;
  - definition of error bars as s.d. or s.e.m.

Please complete ALL of the questions below.
Select "Not Applicable" only when the requested information is not relevant for your study.

Materials

Newly Created Materials	Information included in the manuscript?	In which section is the information available? (Reagents and Tools Table, Materials and Methods, Figures, Data Availability Section)
New materials and reagents need to be available; do any restrictions apply?	Not Applicable	No

Antibodies	Information included in the manuscript?	In which section is the information available? (Reagents and Tools Table, Materials and Methods, Figures, Data Availability Section)
For **antibodies** provide the following information:<br>- Commercial antibodies: RRID (if possible) or supplier name, catalogue number and or/clone number<br>- Non-commercial: RRID or citation	Yes	Material and Methods

DNA and RNA sequences	Information included in the manuscript?	In which section is the information available? (Reagents and Tools Table, Materials and Methods, Figures, Data Availability Section)
**Short novel DNA or RNA including primers, probes**: provide the sequences.	Yes	Material and Methods

Cell materials	Information included in the manuscript?	In which section is the information available? (Reagents and Tools Table, Materials and Methods, Figures, Data Availability Section)
**Cell lines:** Provide species information, strain. Provide accession number in repository **OR** supplier name, catalog number, clone number, and/**OR** RRID.	Yes	Material and Methods
**Primary cultures:** Provide species, strain, sex of origin, genetic modification status.	Not Applicable	
Report if the cell lines were recently **authenticated** (e.g., by STR profiling) and tested for mycoplasma contamination.	Yes	Material and Methods

Experimental animals	Information included in the manuscript?	In which section is the information available? (Reagents and Tools Table, Materials and Methods, Figures, Data Availability Section)
**Laboratory animals or Model organisms:** Provide species, strain, sex, age, genetic modification status. Provide accession number in repository **OR** supplier name, catalog number, clone number, **OR** RRID.	Yes	Material and Methods
**Animal observed in or captured from the field:** Provide species, sex, and age where possible.	Not Applicable	
Please detail **housing and husbandry conditions**.	Yes	Material and Methods, Figures

Plants and microbes	Information included in the manuscript?	In which section is the information available? (Reagents and Tools Table, Materials and Methods, Figures, Data Availability Section)
**Plants:** provide species and strain, ecotype and cultivar where relevant, unique accession number if available, and source (including location for collected wild specimens).	Not Applicable	
**Microbes:** provide species and strain, unique accession number if available, and source.	Not Applicable	

Human research participants	Information included in the manuscript?	In which section is the information available? (Reagents and Tools Table, Materials and Methods, Figures, Data Availability Section)
If collected and within the bounds of privacy constraints report on age, sex and gender or ethnicity for all study participants.	Not Applicable	

Core facilities	Information included in the manuscript?	In which section is the information available? (Reagents and Tools Table, Materials and Methods, Figures, Data Availability Section)
If your work benefited from core facilities, was their service mentioned in the acknowledgments section?	Not Applicable	

Design

Study protocol	Information included in the manuscript?	In which section is the information available? (Reagents and Tools Table, Materials and Methods, Figures, Data Availability Section)
If study protocol has been **pre-registered, provide DOI in the manuscript**. For clinical trials, provide the trial registration number **OR** cite DOI.	Not Applicable	
Report the **clinical trial registration number** (at ClinicalTrials.gov or equivalent), where applicable.	Not Applicable	

Laboratory protocol	Information included in the manuscript?	In which section is the information available? (Reagents and Tools Table, Materials and Methods, Figures, Data Availability Section)
Provide DOI OR other citation details if **external detailed step-by-step protocols** are available.	Yes	Material and Methods

Experimental study design and statistics	Information included in the manuscript?	In which section is the information available? (Reagents and Tools Table, Materials and Methods, Figures, Data Availability Section)
Include a statement about **sample size** estimate even if no statistical methods were used.	Yes	Figures, Appendix tables
Were any steps taken to minimize the effects of subjective bias when allocating animals/samples to treatment (e.g. **randomization procedure**)? If yes, have they been described?	Yes	Material and Methods
Include a statement about **blinding** even if no blinding was done.	Not Applicable	
Describe **inclusion/exclusion criteria** if samples or animals were excluded from the analysis. Were the criteria pre-established?<br><br>If sample or data points were omitted from analysis, report if this was due to attrition or intentional exclusion and provide justification.	Not Applicable	
For every figure, are **statistical tests** justified as appropriate? Do the data meet the assumptions of the tests (e.g., normal distribution)? Describe any methods used to assess it. Is there an estimate of variation within each group of data? Is the variance similar between the groups that are being statistically compared?	Yes	Material and Methods

Sample definition and in-laboratory replication	Information included in the manuscript?	In which section is the information available? (Reagents and Tools Table, Materials and Methods, Figures, Data Availability Section)
In the figure legends: state number of times the experiment was **replicated** in laboratory.	Yes	Figure legends
In the figure legends: define whether data describe **technical or biological replicates**.	Yes	Figure legends

Ethics

Ethics	Information included in the manuscript?	In which section is the information available? (Reagents and Tools Table, Materials and Methods, Figures, Data Availability Section)
Studies involving **human participants**: State details of **authority granting ethics approval** (IRB or equivalent committee(s), provide reference number for approval.	Not Applicable	
Studies involving **human participants**: Include a statement confirming that **informed consent** was obtained from all subjects and that the experiments conformed to the principles set out in the WMA Declaration of Helsinki and the Department of Health and Human Services Belmont Report.	Not Applicable	
Studies involving **human participants:** For publication of **patient photos**, include a statement confirming that consent to publish was obtained.	Not Applicable	
Studies involving experimental **animals**: State details of **authority granting ethics approval** (IRB or equivalent committee(s), provide reference number for approval. Include a statement of compliance with ethical regulations.	Not Applicable	
Studies involving **specimen and field samples:** State if relevant **permits** obtained, provide details of authority approving study; if none were required, explain why.	Not Applicable	

Dual Use Research of Concern (DURC)	Information included in the manuscript?	In which section is the information available? (Reagents and Tools Table, Materials and Methods, Figures, Data Availability Section)
Could your study fall under dual use research restrictions? Please check biosecurity documents and list of **select agents and toxins** (CDC): https://www.selectagents.gov/sat/list.htm	Not Applicable	
If you used a select agent, is the security level of the lab appropriate and reported in the manuscript?	Not Applicable	
If a study is subject to dual use research of concern regulations, is the name of the **authority granting approval and reference number** for the regulatory approval provided in the manuscript?	Not Applicable	

Reporting

The MDAR framework recommends adoption of discipline-specific guidelines, established and endorsed through community initiatives. Journals have their own policy about requiring specific guidelines and recommendations to complement MDAR.

Adherence to community standards	Information included in the manuscript?	In which section is the information available? (Reagents and Tools Table, Materials and Methods, Figures, Data Availability Section)
State if relevant guidelines or checklists (e.g., **ICMJE, MIBBI, ARRIVE, PRISMA**) have been followed or provided.	Not Applicable	
For **tumor marker prognostic studies,** we recommend that you follow the **REMARK** reporting guidelines (see link list at top right). See author guidelines, under 'Reporting Guidelines'. Please confirm you have followed these guidelines.	Not Applicable	
For **phase II and III randomized controlled trials**, please refer to the **CONSORT** flow diagram (see link list at top right) and submit the CONSORT checklist (see link list at top right) with your submission. See author guidelines, under 'Reporting Guidelines'. Please confirm you have submitted this list.	Not Applicable	

Data Availability

Data availability	Information included in the manuscript?	In which section is the information available? (Reagents and Tools Table, Materials and Methods, Figures, Data Availability Section)
Have **primary datasets** been deposited according to the journal's guidelines (see 'Data Deposition' section) and the respective accession numbers provided in the Data Availability Section?	Yes	Source data files and Appendix File
Were **human clinical and genomic datasets** deposited in a public access-controlled repository in accordance to ethical obligations to the patients and to the applicable consent agreement?	Not Applicable	
Are **computational models** that are central and integral to a study available without restrictions in a machine-readable form? Were the relevant accession numbers or links provided?	Not Applicable	
If publicly available data were reused, provide the respective **data citations in the reference list**.	Not Applicable	
